# Factors influencing adherence to clinical practice guidelines in patients with suspected chronic coronary syndrome: a qualitative interview study in the ambulatory care sector in Germany

**DOI:** 10.1186/s12913-023-09587-1

**Published:** 2023-06-20

**Authors:** Marie Naumann, Simon Robin Scharfenberg, Yana Seleznova, Bastian Wein, Oliver Bruder, Stephanie Stock, Dusan Simic, Benjamin Scheckel, Dirk Müller

**Affiliations:** 1 Institute for Health Economics and Clinical Epidemiology, Faculty of Medicine and University Hospital Cologne, University of Cologne, Gleueler Straße 176-178, 50935 Cologne, Germany; 2 Cardiology – Faculty of Medicine, University of Augsburg, Stenglinstrasse 2, 86156 Augsburg, Germany; 3 Department of Cardiology and Angiology, Contilia Heart and Vascular Center, Elisabeth-Hospital Essen, Klara-Kopp-Weg 1, 45138 Essen, Germany; 4 Ruhr University Bochum, Bochum, Germany

**Keywords:** Ambulatory care, Cardiologists, Clinical practice guidelines, Coronary artery disease, Chronic coronary syndrome, General practitioners, Guideline adherence, Interviews, Qualitative content analysis, Qualitative research

## Abstract

**Background:**

Chronic coronary syndrome (CCS) is a potentially progressive clinical presentation of coronary artery disease (CAD). Clinical practice guidelines (CPGs) are available for prevention, diagnosis, and treatment. Embedded in the “ENLIGHT-KHK” healthcare project, a qualitative study was conducted to identify factors that influence guideline adherence from the perspective of general practitioners (GPs) and cardiologists (CA) in the ambulatory care sector in Germany.

**Methods:**

GPs and CAs were surveyed via telephone using an interview guide. The respondents were first asked about their individual approach to caring for patients with suspected CCS. Subsequently, the accordance of their approach with guideline recommendations was addressed. Finally, potential measures for assisting with guideline adherence were discussed. The semi-structured interviews were transcribed verbatim and analysed using a qualitative content analysis in accordance with Kuckartz and Rädiker. Factors influencing adherence to CPGs were categorised by assessing whether they (i) inhibited or facilitated guideline adherence, (ii) played a role in patients at risk of CCS or with suspected or known CCS, (iii) were mentioned in implicit or explicit thematic reference to CPGs, and (iv) were declared a practical problem.

**Results:**

Based on interviews with ten GPs and five CAs, 35 potential influencing factors were identified. These emerged at four levels: patients, healthcare providers, CPGs, and the healthcare system. The most commonly cited barrier to guideline adherence among the respondents was structural aspects at the system level, including reachability of providers and services, waiting times, reimbursement through statutory health insurance (SHI) providers, and contract offers. There was a strong emphasis on interdependencies between factors acting at different levels. For instance, poor reachability of providers and services at the system level may result in inexpedience of guideline recommendations at the CPG level. Likewise, poor reachability of providers and services at the system level may be aggravated or alleviated by factors such as diagnostic preferences at the patient level or collaborations at the provider level.

**Conclusions:**

To assist with adherence to CPGs regarding CCS, promoting measures may be needed that account for interdependencies between barriers and facilitators at various healthcare levels. Respective measures should consider medically justified deviations from guideline recommendations in individual cases.

**Trial registration:**

German Clinical Trials Register: DRKS00015638; Universal Trial Number (UTN): U1111-1227-8055.

**Supplementary Information:**

The online version contains supplementary material available at 10.1186/s12913-023-09587-1.

## Background

Coronary artery disease (CAD) at a global level has a high and, at times increasing, prevalence, incidence, and mortality [1, 2]. CAD is therefore considered to be a major public health burden [3]. CAD may, inter alia, present as chronic coronary syndrome (CCS) – also referred to as stable CAD – which poses a complex clinical issue: patients’ complaints are characterised by cardiac symptoms (e.g. chest pain or dyspnoea) and asymptomatic progression, especially in women [4]. Both of these characteristics can also be related to other diseases, which makes the prevention, diagnosis, and treatment of CCS difficult. In light of this issue, national and international clinical practice guidelines (CPGs) provide evidence-based recommendations for healthcare providers.

For Germany, there are two relevant guidelines that recommend a symptom and pre-test-based algorithmic diagnostic approach [5, 6]: first, a national CPG on stable CAD by the German Federal Medical Association (NVL-CAD) [7], which is classified as a S3-guideline by the German Association of the Scientific Medical Societies (i.e. as a guideline that meets the highest standard according to the four-class measurement of the association) [8]. Second, a well-established CPG on the diagnosis and management of CCS by the European Society of Cardiology (ESC-CCS) [9]. The 2019 ESC-CCS is endorsed by the German National Cardiac Society [10] and the 2013 version of the ESC-CCS was utilised in the development of the NVL-CAD [7, 11].

Among several similarities, both CPGs recommend invasive coronary angiography (ICA) via cardiac catheterisation for diagnostic and treatment purposes in high-risk patients. However, according to both guidelines, in patients with intermediate pre-test probabilities, initial non-invasive ischemia testing (NIT) is considered mandatory. Stress echocardiography, coronary computed tomography angiography (CCTA), cardiac stress magnetic resonance imaging (CMRI), and myocardial perfusion scintigraphy (MPS) are all considered appropriate procedures. However, rates of ICA in Germany are continuously higher than those in other European countries, and there seems to be a significant correlation between regionally available ICA capacity and the use of said procedure, especially as a treatment for CCS [12]. This may be an indication of overuse or misuse of ICA, i.e. non-adherence to relevant CPGs [12, 13].

The “ENLIGHT-KHK” healthcare project was set up to evaluate guideline adherence in patients with presumed CCS in Germany based on the NVL-CAD version from 2019 and the ESC-CCS from 2019 (i.e. the versions of the CPGs that were available at the time the project was conceptualised) [14]. Between June 2018 and June 2022, two studies that provide an exemplary trans-sectoral insight into current healthcare practice were carried out: a prospective observational study in specialist cardio-vascular hospitals and a qualitative interview study with general practitioners (GPs) and cardiologists (CAs) in the ambulatory care sector (i.e. physicians with a medical practice of their own who provide outpatient services). The observational study examined adherence to CPGs in patients with presumed obstructive stable CAD who had undergone ICA. The interview study explored factors that influence adherence to CPGs in patients with suspected CCS.

## Methods

The reporting of the study is in accordance with COREQ [15]. See Additional File 1.

### Aim

Focusing on ambulatory care in Germany, the objective of the qualitative interview study was to explore factors that influence adherence to NVL-CAD and ESC-CCS in patients with suspected CCS. GPs (i.e. primary care providers) and CAs (i.e. secondary care providers) were targeted as respondents for the interviews. They were first asked about their individual approach to caring for patients with suspected CCS. Subsequently, the accordance of their approach with guideline recommendations was addressed. Finally, potential measures for assisting with guideline adherence were discussed.

The following questions guided the research process:

- Which factors **inhibit or facilitate** guideline adherence (*normative influence*)?
- Which factors are related to **patients at risk of CCS or with suspected or known CCS** (*processual influence*)?
- Is a factor mentioned in **implicit or explicit thematic reference** to CPGs regarding healthcare for CCS (*thematic relevance*)?
- Is a factor perceived as a **practical problem** within healthcare for CCS (*practical relevance*)?

By answering these questions, the study could provide evidence on whether and to what extent measures are needed to promote adherence to CPGs regarding CCS.

### Design and setting

A recent meta-review documented that there is a large body of literature on factors influencing the implementation and uptake of CPGs [16]. Evidence with a specific focus on the adherence to CPGs regarding CCS on behalf of GPs and CAs in ambulatory care in Germany remains scarce; previous studies were often conducted in other countries where healthcare sectors may differ [17–20], or focused on the patient perspective [21]. A qualitative design was therefore chosen for this study. This allows for an in-depth exploration of unknown empirical phenomena [22]. In accordance with the notion of a dialectical relationship between context, intervention, and outcome(s) [23], the collection of primary interview data was a fundamental imperative. This enabled emphasis to be placed on the scope of application (context) for a future measure (intervention) aimed at promoting adherence to CPGs regarding CCS (outcome).

**Fig. 1:**
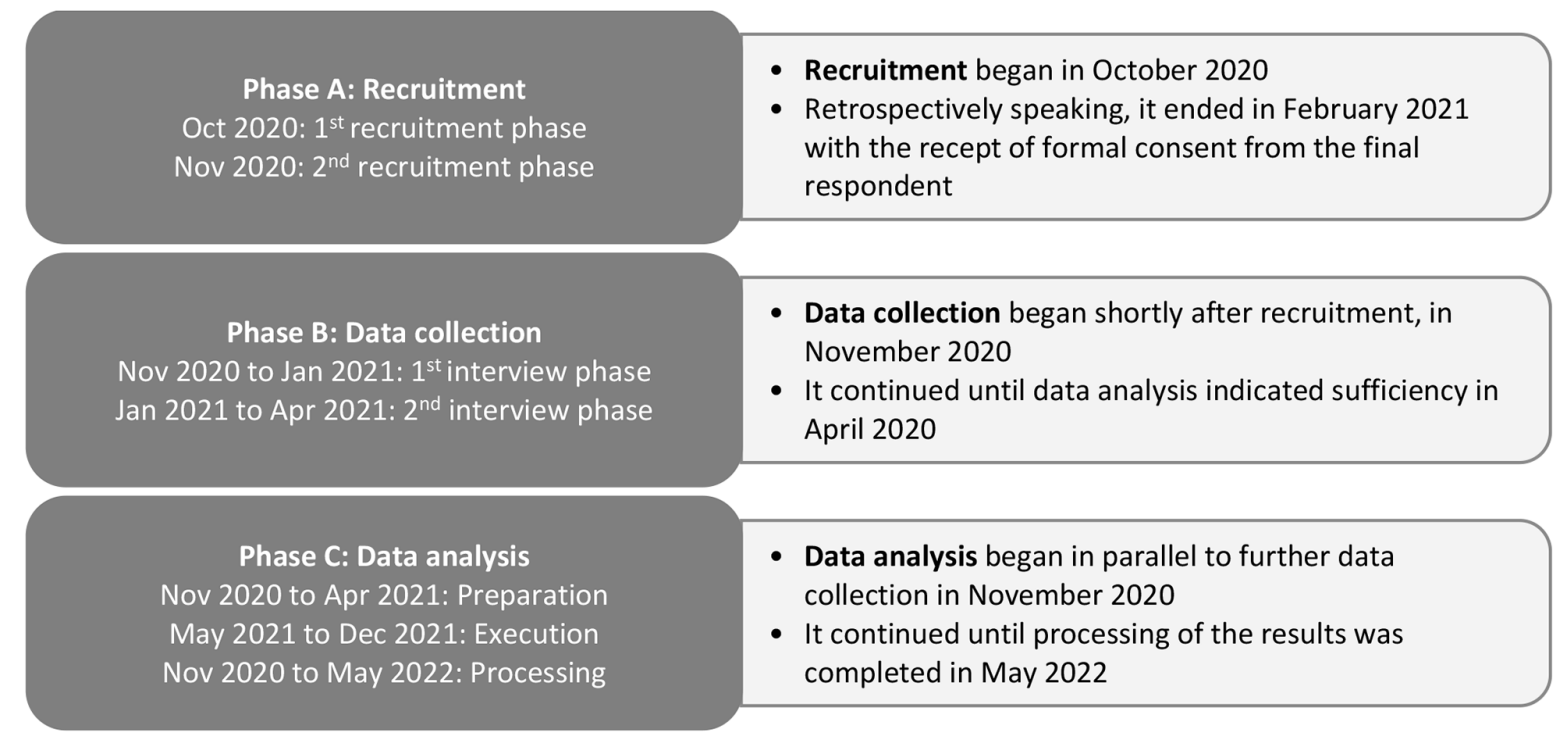
Procedure for the qualitative interview study

The interviews were conducted in German and the study lasted twenty months. The study was divided into three main phases: recruitment, data collection, and data analysis (see Fig. 1).

#### Phase A: recruitment

A maximum sample size of fifteen to twenty respondents was planned due to the physicians’ time constraints [24]. A financial compensation of 200€ per respondent was offered.

In accordance with Moser and Korstjens [25], the sampling strategy consisted of an upstream criterion approach and a downstream snowball approach. The respondents were eligible if they were in possession of a license to participate in outpatient medical care for persons insured under the statutory health insurance (SHI) scheme and practiced within the empirical scope of ENLIGHT-KHK [14]. Based on these two criteria, potential respondents were recruited via recognised physicians’ networks. These are regional groups of various health professionals that are accredited by the National Association of Statutory Health Insurance Physicians. Supplementary recruitment took place via partners at the hospitals that acted as recruitment centres for the observational study within ENLIGHT-KHK.

#### Phase B: data collection

Semi-structured interviewing was chosen as the mode for the data collection. This method allows for an exploratory collection of in-depth information on the perspectives of social actors, i.e. certain attitudes, beliefs, experiences, and/or perceptions. Furthermore, it ensures coverage of various topics that are important to a respective research aim thanks to the use of an interview guide [22, 26, 27]. Due to contact restrictions during the Covid-19 pandemic, the participants were interviewed by telephone on an individual basis. Compared to alternative modes for qualitative interviews (e.g. face-to-face-interviews or interviews via digital applications), telephone interviews are more transparent, are convenient for both the interviewer and the respondent, and may enable respondents to talk about sensitive topics more freely (e.g. adherence to CPGs as a political issue) due to greater perceived anonymity [28, 29].

There was no established relationship between the interviewer and the respondents prior to the interview. The respondents were made aware of the procedures of the qualitative interview study and the wider project setting before the interview took place. The information provided in this context concerned aspects such as the aims of the study, the roles of the participating project partners, data management, and funding. Formal consent was obtained from the respondents prior to the interview. All the interviews were documented as audio recordings, which subsequently acted as the basis for verbatim transcription. Transcription began immediately after the interview, and was guided by rules provided by Dresing and Pehl [30]. Finally, the respondents were given the opportunity to review the interview transcript prior to its entry into the data corpus.

#### Phase C: data analysis

A qualitative content analytical approach was used for the data analysis. This allows for interpretation of personal meaning, i.e. reflection of the subjective perspectives of social individuals [31]. Among various qualitative content analytical approaches [32], the work of Kuckartz and Rädiker was chosen as guidance [33, 34]. This proposes an approach based on an extensive methodological groundwork and consecutive methodical principles. At its core is a computer-assisted iterative analysis process performed with the aid of the MAXQDA software (VERBI GmbH, Berlin/Germany) [33], which allows for continuous analytical reflection throughout the qualitative research process [27, 35].

**Fig. 2:**
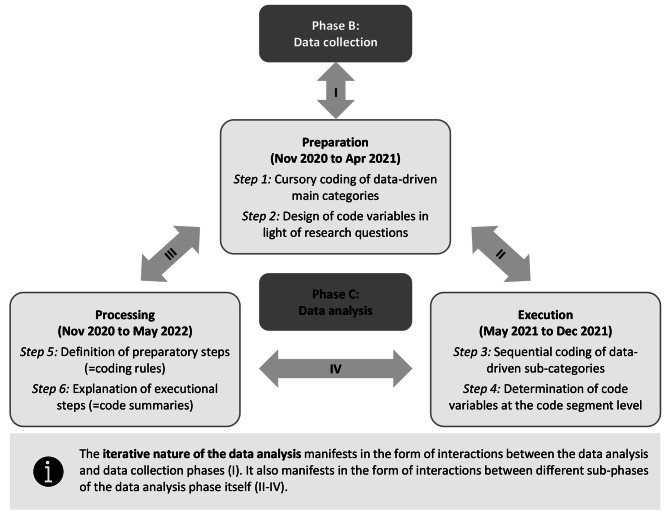
Iteration process

Based on Kuckartz’s model for an iterative thematic qualitative text analysis [33], the data analysis was subdivided in three sub-phases (see Fig. 2). The respondents themselves did not participate in these sub-phases.

The first sub-phase (*preparation*) began simultaneously with the data collection. In this sub-phase, data-driven main categories were developed for potential influencing factors by means of cursory coding of the evolving data corpus. This mode allowed for the uncovering of modifications required to the initial interview guide (i.e. redundancies or needs for specification). Furthermore, four code variables were designed as a preparatory step (see Table 1). The code variable design replicates the four guiding research questions.

**Table 1:** Code variables

Code variable	Code variable value	Aid for determination: based on the respective code segment …
*Normative influence*	Indeterminate	it remains *unclear* if guideline adherence is inhibited and/or facilitated.
	Undecided	it appears that the influence on guideline adherence is *ambivalent*.
	Contra	it appears that guideline adherence is *clearly inhibited*.
	Pro	it appears that guideline adherence is *clearly facilitated*.
*Processual influence*	Avoidance	it appears that *diagnostics* for patients with an anticipated *increased risk of CCS* are addressed.
	Clarification	it appears that *diagnostics* for patients with *suspected or known CCS* are addressed.
	Treatment	it appears that *therapy* for patients with *known CCS* is addressed.
*Thematic relevance*	Implicit	it appears that the respondent’s statement *rather implicitly refers to the topic of CPGs*. The respondent’s argumentations may extend over several paragraphs, for example, and start with an explicit reference to CPGs. However, they may go on or end with paragraphs that include no further explicit reference to CPGs.
	Explicit	it appears that the interviewer’s question and/or the respondent’s statement *rather explicitly refers to the topic of CPGs*. The respondent’s argumentations may extend over several paragraphs that recurrently include explicit reference to CPGs, for example.
*Practical relevance*	Not given	it appears that the respondent’s statement *does not reflect an inhibiting factor as a problem* in healthcare practice. This may become apparent through *relativising wording* with regard to the potential impact of an inhibiting factor (e.g. “However, this problem can be easily overcome”).
	Given	it appears that the respondent’s statement *does reflect an inhibiting factor as a problem* in healthcare practice. This may become apparent through *exaggerating wording* with regard to the potential impact of an inhibiting factor (e.g. “And the problem is, this cannot be overcome easily”).

In the second sub-phase (*execution*), data-driven sub-categories of potential influencing factors were developed by means of sequential coding of the completed data corpus. Based on the respondents’ statements, potential influencing factors were identified, designated, and specified using the designed code variables. Each of the influencing factors was then allocated to a main category. In order to foster intersubjective comprehensibility as a central quality maxim [33], this process was carried out by two coders. Initially, each coder created sub-categories and determined respective code variable values per code segment independently. The two coders then consented their findings. This collaborative coding process took place in small rounds of one to two interviews until inter-coder agreement was achieved across all the interviews. Finally, both coders checked the pre-final category system and code variable values for consistency and precision. This involved steps such as a review of the semantic exclusiveness of the sub-categories and a complete determination of the code variable values. At this point, a mediator was consulted in case the two coders encountered any problems reaching a decision.

As a central technique within qualitative research processes [36], memo writing was a crucial step in the third and final sub-phase (*processing*). In the form of coding rule memos, this was used to define the preparatory analysis steps. In addition to this, code summaries were written to explain the results of the executional analysis steps.

## Results

Based on the obtained data corpus, a multitude of diverse factors that influence adherence to NVL-CAD and ESC-CCS were identified.

### Description of findings

This section will begin with a description of the characteristics of the study sample. Thereafter, the central logic of the category system will be outlined, followed by a general description of tendencies with regard to the determined code variable values. Finally, patterns concerning coding relations will be depicted.

#### Study sample

In total, ten GPs and five CAs with different socio-demographic and professional characteristics were interviewed (see Table 2). There were no respondents who refused to participate or dropped out after giving formal consent to their participation in the study.

**Table 2:** Respondent characteristics

Gender	n	(%)
Male	10	(67%)
Female	5	(33%)
Other	0	(0%)
**Year of birth**	**Values**	
Mean value	1967	
Lowest value	1955	
Highest value	1983	
Standard deviation	8	
**Years of professional experience**	**Values**	
Mean value	23	
Lowest value	10	
Highest value	39	
Standard deviation	9	
**Organisation of own practice** ^a^	**n**	**(%)**
Individual practice	2	(13%)
Joint practice (same professions)	12	(80%)
Joint practice (different professions)	1	(7%)
Medical care centre	0	(0%)
**Cooperation(s) between own practice others (multiple choice possible)** ^a^	**n**	**(%)**
Cooperation with joint practice	2	(13%)
Cooperation within practice network	12	(80%)
Cooperation within apparatus/equipment community	4	(26%)
Cooperation within laboratory community	7	(47%)
Cooperation with outpatient surgery centre	1	(7%)
Cooperation with hospital	6	(40%)
Own practice has no cooperations	0	(0%)
**Number of residents at practice location** ^b^	**n**	**(%)**
Rural municipality (less than 5000)	2	(13%)
Small town (equal to or greater than 5000 but less than 19,999)	1	(7%)
Medium-sized city (equal to or greater than 20,000 but less than 99,999)	6	(40%)
Large city (equal to or greater than 100,000)	6	(40%)
**Scope of employment**	**n**	**(%)**
Full time	14	(93%)
Part time	1	(7%)

Legend: Data is derived from a short questionnaire regarding central socio-demographic and professional characteristics. All the respondents (N = 15) completed this questionnaire prior to their respective interviews; ^(a)^ Item is based on a questionnaire used within an annual survey by the German Federal Statistical Office. It is focused on cost structures in the ambulatory sector in Germany; ^(b)^ Item is based on a heuristic differentiation by the German Federal Office for Building and Regional Planning. It is focused on city and community types in Germany

Based on an initial interview guide, the first interview phase took place between November 2020 and January 2021. After this, the interview guide was adapted for a second interview phase, which took place between January 2021 and April 2021. See Additional File 2.

The fifteen interviews had a mean duration of 42 min (range: 29 to 47 min, standard deviation: 7 min). Each respondent was only interviewed once. One respondent made use of the option to review their interview transcript prior to its entry into the data corpus (no change requests were made).

#### Category system

**Table 3:** Category system (incl. coding frequencies)

Code categories		n (%) per interview		n (%) per code segment	
*Level 1: Patients*					
*Mode: Filters for action*					
*1*	Health literacy	5	(33%)	10	(1%)
*2*	Mobility	1	(7%)	1	(0%)
*Mode: Forms of action*					
*3*	Lifestyle	5	(33%)	10	(1%)
*4*	Medication intake	1	(7%)	2	(0%)
*Mode: Motives for action*					
*5*	Mentality	2	(13%)	5	(1%)
*6*	Self-interest	9	(60%)	26	(4%)
*Level 2: Healthcare providers*					
*Mode: Filters for action*					
*7*	Case-related time pressure	5	(33%)	8	(1%)
*8*	Constitution of patients	5	(33%)	7	(1%)
*9*	Personal situation of patients	1	(7%)	1	(0%)
*10*	Relationship with patients	5	(33%)	9	(1%)
*Mode: Forms of action*					
*11*	Integral healthcare	2	(13%)	3	(0%)
*12*	Interprofessional healthcare	13	(87%)	41	(6%)
*13*	Stratified healthcare	15	(100%)	153	(21%)
*Mode: Motives for action*					
*14*	Acceptance of CPGs	5	(33%)	8	(1%)
*15*	Evidence orientation	12	(80%)	57	(8%)
*16*	Explicit knowledge	10	(67%)	27	(4%)
*17*	Implicit knowledge	9	(60%)	20	(3%)
*18*	Proactivity	3	(20%)	9	(1%)
*19*	Professional responsibility	4	(27%)	6	(1%)
*20*	Profitability	11	(73%)	35	(5%)
*21*	Prudence	5	(33%)	13	(2%)
*Level 3: CPGs*					
*Mode: Application characteristics*					
*22*	Inconsistency	1	(7%)	3	(0%)
*23*	Inexpedience	5	(33%)	14	(2%)
*24*	Non-binding nature	1	(7%)	2	(0%)
*25*	Reliability	2	(13%)	2	(0%)
*Mode: Development characteristics*					
*26*	Abstract nature	2	(13%)	4	(1%)
*27*	Ambiguity	2	(13%)	2	(0%)
*28*	Incompleteness	2	(13%)	3	(0%)
*29*	Ostensible clarity	3	(20%)	4	(1%)
*Level 4: Healthcare system*					
*Mode: Healthcare processes*					
*30*	Effort (procedural)	10	(67%)	22	(3%)
*31*	Workload (administrative)	5	(33%)	11	(1%)
*Mode: Healthcare structures*					
*32*	Economic structures	13	(87%)	57	(8%)
*33*	Local structures	13	(87%)	47	(6%)
*34*	Stipulated structures	13	(87%)	46	(6%)
*35*	Temporal structures	12	(80%)	68	(9%)

Legend: Code categories are presented in alphabetic order. Coding frequencies are displayed per interview (N = 15) and per code segment (N = 736)

Overall, 35 factors that could potentially influence adherence to NVL-CAD and ESC-CCS were identified (see Table 3). Hereinafter, numbers in curly brackets shall be used to indicate which of the 35 influencing factors are being cited.

Four levels were defined as the main categories for potential influencing factors: ‘patients’ and ‘healthcare providers’ as social actors whose action (i.e. moment of behaviour) “is ‘social’ insofar as its subjective meaning takes account of the behaviour of others and is thereby oriented in its course” [37] {1–6; 7–21}; ‘CPGs’ as instruments {22–29}; and the ‘healthcare system’ as an institution {30–35}.

Within each of these levels, specific modes of manifestation were distinguished as first-order sub-categories. At the patient and provider levels, these were *filters for action*, which delineate horizons of individual action {1–2; 7–10}; *forms of action*, which depict individual practices of action {3–4; 11–13}; and *motives for action*, which drive individual action {5–6; 14–21}. At the CPG level, two modes were differentiated: *application characteristics*, which address practical recommendations for CPGs {22–25}; and *development characteristics*, which address rationales for CPGs {26–29}. Two modes were also distinguished at the system level: *healthcare processes* as comparatively rigid courses of action {30–31}; and *healthcare structures* as comparatively rigid scopes of action {32–35}.

Finally, specifications of these manifestation modes were coded as second-order sub-categories. These are the 35 factors identified as influencing adherence to NVL-CAD and ESC-CCS. In terms of coding frequencies, system factors were coded for all the interviews. CPG factors were coded for a small number of interviews.

For an overview of inter-coder agreement during the early development of this categorial logic, see Additional File 3. The central rules used as guidance for the coding process are summarised in Additional File 4.

#### Code variable values

The 35 identified influencing factors appear as code segments within the data corpus (N = 736). Each code segment was specified using four code variables: *normative influence*, *processual influence*, *thematic relevance*, and *practical relevance* (see Table 1). Based on the respective frequencies of the code variable values, each of the 35 influencing factors had a dominant value for each of the four code variables.

As shown in Table 4, most of the factors were perceived as barriers to guideline adherence (*normative influence*) in cases of clarification of suspected or known CCS (*processual influence*), particularly at the provider level {i.e. 30 and 32 − 25}. However, across all four levels, several factors were unequivocally described as facilitators of guideline adherence {e.g. 2, 11, or 25}, or at least as factors with ambivalent {e.g. 1, 8 or 35} or unclear {e.g. 7 or 16} influence on guideline adherence. Furthermore, there were several factors that were not only associated with clarification of CCS, but also related to prevention and/or treatment {e.g. 3, 18, 28, or 31}.

As shown in Table 5, CPGs was rarely an explicit topic (*thematic relevance*) in any of the 35 influencing factors except those at the CPG level, which referred to this topic inherently {22–29}. Moreover, the majority of influencing factors that were perceived as potential barriers to guideline adherence were not necessarily perceived as a practical problem (*practical relevance*). This means that some barriers were perceived as inhibiting factors that could be more or less managed by the respondents themselves {e.g. 9 or 24}. Perceptions regarding barriers at the system level differed from this, however. These were perceived as relatively rigid and inflexible factors that often resulted in practical problems for healthcare providers {30–34}.

**Table 4:** Normative and processual influence

Influencing factor		Normative influence^a^							Processual influence^b^			
		PR	CO		UN	IN		*Dominant* ^c^	AV	CL	TR	*Dominant* ^c^
*1*	*Health literacy*	47%	43%		10%			PR	10%	63%	27%	CL
*2*	*Mobility*	100%						PR		100%		CL
*3*	*Lifestyle*		100%					CO	44%	6%	50%	TR
*4*	*Medication intake*		50%		50%			CO/UN			100%	TR
*5*	*Mentality*	25%	75%					CO		67%	33%	CL
*6*	*Self-interest*	11%	89%					CO	11%	78%	11%	CL
*7*	*Case-related time pressure*					100%		IN		100%		CL
*8*	*Constitution of patients*		20%		80%			UN		100%		CL
*9*	*Personal situation of patients*		100%					CO		100%		CL
*10*	*Relationship with patients*					100%		IN		93%	7%	CL
*11*	*Integral healthcare*	100%						PR		100%		CL
*12*	*Interprofessional healthcare*	54%	46%					PR		93%	7%	CL
*13*	*Stratified healthcare*	100%						PR	3%	88%	9%	CL
*14*	*Acceptance of CPGs*	100%						PR		50%	50%	CL/TR
*15*	*Evidence orientation*	100%						PR	3%	86%	11%	CL
*16*	*Explicit knowledge*					100%		IN		87%	13%	CL
*17*	*Implicit knowledge*					100%		IN		100%		CL
*18*	*Proactivity*	33%	67%					CO	33%	67%		CL
*19*	*Professional responsibility*		100%					CO		100%		CL
*20*	*Profitability*					100%		IN	17%	77%	6%	CL
*21*	*Prudence*					100%		IN		100%		CL
*22*	*Inconsistency*		100%					CO			100%	TR
*23*	*Inexpedience*		100%					CO		96%	4%	CL
*24*	*Non-binding nature*		100%					CO		100%		CL
*25*	*Reliability*	100%						PR		100%		CL
*26*	*Abstract nature*		100%					CO		100%		CL
*27*	*Ambiguity*		100%					CO		100%		CL
*28*	*Incompleteness*		100%					CO		50%	50%	CL/TR
*29*	*Ostensible clarity*		100%					CO		100%		CL
*30*	*Effort (procedural)*	23%	77%					CO		100%		CL
*31*	*Workload (administrative)*		100%					CO	12%	28%	60%	TR
*32*	*Economic structures*	4%	96%		2%			CO	4,6%	90,5%	4,9%	CL
*33*	*Local structures*	29%	69%					CO	9%	89%	2%	CL
*34*	*Stipulated structures*	29%	71%					CO	40%	47%	13%	CL
*35*	*Temporal structures*	40%	60%					CO	2%	92%	6%	CL
**Example calculation**	**Health literacy**	**Normative influence**										
		PR		CO					UN		IN	
	Interview 2	1	1	0			0		0	0	0	0
	Interview 3	1	1/2	1			1/2		0	0	0	0
	Interview 5	1	1/3	2			2/3		0	0	0	0
	Interview 6	0	0	2			1		0	0	0	0
	Interview 11	1	1/2	0			0		1	1/2	0	0
	**TOTAL**	**4**	**2 1/3**	**5**			**2 1/6**		**1**	**1/2**	**0**	**0**

Legend: ^(a)^ Code variable with “Pro” (PR), “Contra” (CO), “Undecided” (UN), and “Indeterminate” (IN) as possible values; ^(b)^ Code variable with “Avoidance” (AV), “Clarification” (CL), and “Treatment” (TR) as possible values; ^(c)^ Equates the highest percentual relative frequency of a possible code variable value. Relative frequencies were calculated based on weighted absolute frequencies. For instance, “Health literacy” was coded 10 times across 5 interviews. Each of these 10 code segments was determined as “PR”, “CO”, “UN”, or “IN”. In the example calculation, this is depicted as unweighted absolute frequencies in the left column of each code variable value. However, code segments within an interview, might be coded more frequently due to stylistic repetition means, for example. As such, code segments must be weighted evenly for each respondent. In the example calculation, this is depicted as weighted absolute frequencies in the right column of each code variable value. In terms of “Health literacy”, “PR” subsequently became the dominant value instead of “CO” (ex-ante: 4 vs. 5; ex-post: 2 1/3 vs. 2 1/6)

**Table 5:** Thematic and practical relevance

Influencing factor		Thematic relevance^a^			Practical relevance^b^		
		IM	EX	*Dominant* ^c^	NO	GI	*Dominant* ^c^
*1*	*Health literacy*	60%	40%	IM	73%	27%	NO
*2*	*Mobility*	100%		IM	100%		NO
*3*	*Lifestyle*	100%		IM	27%	73%	GI
*4*	*Medication intake*	100%		IM	50%	50%	NO/GI
*5*	*Mentality*	75%	25%	IM	58%	42%	NO
*6*	*Self-interest*	79%	21%	IM	26%	74%	GI
*7*	*Case-related time pressure*	90%	10%	IM	100%		NO
*8*	*Constitution of patients*	53%	47%	IM	27%	73%	GI
*9*	*Personal situation of patients*		100%	EX	100%		NO
*10*	*Relationship with patients*	90%	10%	IM	90%	10%	NO
*11*	*Integral healthcare*		100%	EX	100%		NO
*12*	*Interprofessional healthcare*	77%	23%	IM	57%	43%	NO
*13*	*Stratified healthcare*	64%	36%	IM	100%		NO
*14*	*Acceptance of CPGs*		100%	EX	100%		NO
*15*	*Evidence orientation*	50%	50%	IM/EX	100%		NO
*16*	*Explicit knowledge*	55%	45%	IM	63%	37%	NO
*17*	*Implicit knowledge*	72%	28%	IM	76%	24%	NO
*18*	*Proactivity*	67%	33%	IM	33%	67%	GI
*19*	*Professional responsibility*	100%		IM	25%	75%	GI
*20*	*Profitability*	63%	37%	IM	100%		NO
*21*	*Prudence*	60%	40%	IM	83%	17%	NO
*22*	*Inconsistency*		100%	EX	100%		NO
*23*	*Inexpedience*		100%	EX	29%	71%	GI
*24*	*Non-binding nature*		100%	EX	100%		NO
*25*	*Reliability*		100%	EX	100%		NO
*26*	*Abstract nature*		100%	EX	83%	17%	NO
*27*	*Ambiguity*		100%	EX	50%	50%	NO/GI
*28*	*Incompleteness*		100%	EX		100%	GI
*29*	*Ostensible clarity*		100%	EX	33%	67%	GI
*30*	*Effort (procedural)*	51%	49%	IM	49%	51%	GI
*31*	*Workload (administrative)*	82%	18%	IM	8%	92%	GI
*32*	*Economic structures*	73%	27%	IM	40%	60%	GI
*33*	*Local structures*	71%	29%	IM	44%	56%	GI
*34*	*Stipulated structures*	76%	24%	IM	44%	56%	GI
*35*	*Temporal structures*	87%	13%	IM	58%	42%	NO

Legend: (a) Code variable with “Implicit” (IM) and “Explicit” (EX) as possible values; (b) Code variable with “Not given” (NO) and “Given” (GI) as possible values; (c) Equates the highest percentual relative frequency of a possible code variable value. Relative frequencies were calculated based on weighted absolute frequencies. For an example calculation, see Table 4 (the method of calculation was identical for all four code variables)

#### Coding relations

Coding relations can be visualised using co-occurrences of code segments. By limiting the counting of co-occurrences of code segments to a maximum distance of one paragraph, three key aspects could be observed. Firstly, co-occurrence of code segments was more frequent overall with regard to influencing factors at the same level. Secondly, this became especially apparent with regard to co-occurrence of code segments at the system level in the intra-level view {30–35}. Thirdly, code segments at the provider {7–21} and system levels {30–35} co-occurred most frequently in the inter-level view.

For an overview of the described coding relations, including frequency calculation procedures, see Additional File 5.

### Interpretation of findings

This sub-chapter is structured according to the defined four levels of influencing factors. Based on the findings described above, it provides interpretations of central argumentative logics stated by the respondents. It will also illustrate examples of identified influencing factors. For an overview of all the potentially influencing factors identified, see Additional File 6.

#### Level 1: patients

**Fig. 3:**
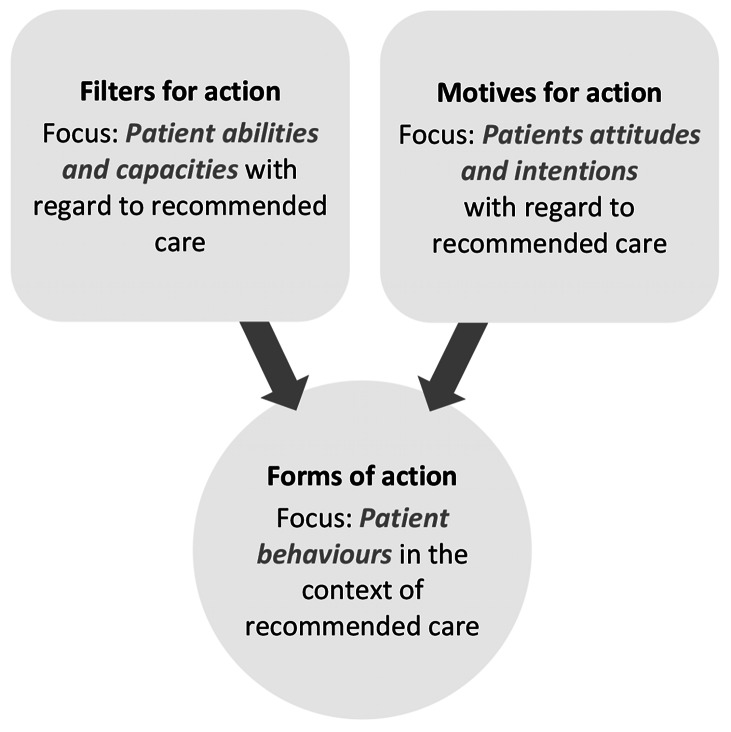
Influencing factors at the patient level

Factors at the patient level were coded occasionally throughout the interviews. Overall, the respondent perspectives on patient action and its consequences for guideline adherence varied. However, as Fig. 3 shows, a focus was placed on the conditional aspects of patient action with regard to recommended care: firstly, the abilities and capacities of patients as filters for their actions and, secondly, patient attitudes and intentions as motives for their actions.

For instance, the ability of patients to handle health-related information was generally viewed as a facilitator for guideline-adherent clarification of CCS from both the perspective of GPs and CAs {1}. In concrete terms, if patients *“can exactly tell you when and on what occasion they had what kind of complaints” (B11, CA)*, there may be a greater chance of making a correct suspected diagnosis based on the patient’s medical history. A sufficient level of disease awareness in patients with known CCS may further aid clarification, since *“they already know the symptoms. And have experienced a crisis situation already” (B05, GP)*. However, the respondents did not rule out the possibility of this factor becoming an inhibiting influence in cases where the patient lacks sufficient disease awareness. As the respondents indicated, a correct suspected diagnosis may be delayed or sometimes prevented if the patients *“have no previous experience of this [i.e. CCS] and then may also have linked the symptoms to other organs” (B05, GP)*.

In turn, the personal intentions of the patient were generally seen as a barrier to guideline-adherent clarification {6}, for example. On the one hand, this pertained to preferences for known and/or specialised physicians which were predominantly mentioned by GPs. For instance, *“many patients insist on seeing a CA in situations where I say: ‘actually, you don’t need to.’ But no, they do not let me talk them out of it and insist on it and so on, right? That is why CAs are so overrun: because everyone who has high blood pressure runs to a CA first. […] But primarily, a blood pressure adjustment, for example, is not a task for a CA. But you really cannot make a lot of people believe that, can you?” (B06, GP)*. On the other hand, preferences for diagnostic procedures were mentioned by both GPs and CAs. For example, the respondents noticed that patients may claim ICA for diagnostic confirmation. This would become apparent *“especially in light of the frequent question: can the person be managed at home? Yes, of course they would prefer that the person has CCS, gets a stent, and then bounces around again like they did when they were forty. But of course, that is not always the explanation. Perhaps there is also a sum of aging processes that simply goes hand in hand with certain impairments, right?” (B11, CA)*. From the respondent’s perspective, the problem in these cases is, that *“in the end, the patients are the ones who decide for themselves: where do they go with their chest pressure complaints? And because their aunt thinks it could be something for the CA, and it could be a heart attack. And their uncle is also/ And they used to/ And so on. Then you go directly to the CA – even believing that you are an urgent patient – and/ Yes, then all this misdirection starts” (B10, GP)*. However, where patients’ intentions fall in line with guideline recommendations, the respondents did, in fact, acknowledge potential for de-problematisation. In the end, *“the question is always: what is the patient’s wish? Or: what is the patient’s order?” (B12, GP)*.

In summary, factors at the patient level may be diametrically opposed to or in line with certain guideline recommendations: disease awareness in patients may fundamentally facilitate or inhibit guideline-adherent diagnostics. Furthermore, patients’ diagnostic preferences may steer functional differentiation between primary and secondary care as recommended by NVL-CAD and ESC-CCS. This also applies to the recommended use of NIT. For instance, a patient seeking diagnostic certainty may force mobilisation of ICA. In patients with intermediately presumed CCS, this would not correspond to the recommendations of the NVL-CAD and ESC-CCS. In this ambivalent sense, patient factors may aggravate or alleviate the influence of identified factors at other levels, particularly those at the system level (see sub-chapter “Level 4: Healthcare system”).

#### Level 2: healthcare providers

**Fig. 4:**
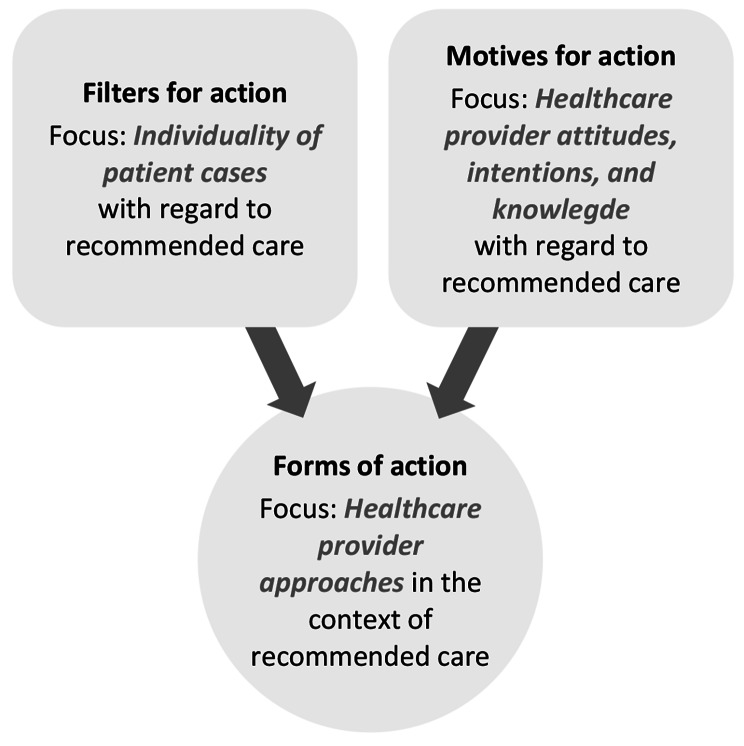
Influencing factors at the provider level

Factors at the provider level were recurrently coded throughout the interviews. The central argumentative logic stated by the respondents at this level is similar to that expressed at the patient level, i.e. while the respondents’ perspectives with regard to healthcare provider action and its consequences for guideline adherence varied, there was a focus on conditional aspects for said action. As Fig. 4 shows, this focus was placed on (i) the individuality of patient cases as a central filter for healthcare provider action and (ii) healthcare provider attitudes, intentions, and knowledge as the motives for their action.

As an example, especially CAs emphasised that, due to each patient’s individual mental and/or physical condition, it may not be possible to use certain diagnostic methods {8}. This may pose a barrier to guideline-adherent clarification. This was especially viewed as a difficulty with regard to stress echocardiography, since *“the patient must offer the prerequisites for it. So, it is different whether you examine someone who is athletic, wiry, and of normal weight, or whether you examine someone who is a heavy smoker with overinflated lungs, which then always push in front of the heart, or someone who is very overweight. Where you cannot even properly depict the heart with the ultrasound, right? And unfortunately, these patients who have this exact heart disease are often long-time smokers or very overweight. And to be honest, they often cannot be examined well using stress echocardiography, right? Although the procedure is very good, it has its limitations” (B08, CA)*. Referring to CMRI was likewise viewed as difficult, since patients may *“be claustrophobic or simply overweight” (B13, CA)*. However, the respondents also noted that, if patients meet the respective criteria for NIT procedures, this has a facilitating influence on guideline adherence. In the end, *“the patient must provide the conditions” (B11, CA)*.

Another example of this ambivalence became apparent when considering healthcare provider knowledge as a potential influencing factor. The respondents stressed that said knowledge plays a role in guideline-adherent diagnostic and therapeutic decision-making. However, they were generally reluctant to specify in detail how it may influence the guideline adherence of the respective decision. This may be because the respondents were referring to a conglomerate of forms of personal knowledge. In accordance with Polanyi [38], two subordinate forms thus became apparent from an analytical perspective. The first of these was explicit knowledge, which is understood as a form of knowledge that is generally manifest and can thus usually be externalised (e.g. in the form of scientific evidence). The second subordinate form of knowledge was implicit knowledge. This is regarded as a more latent form of knowledge, and thus as harder to externalise (i.e. in the form of subconscious ‘know-how’). With this distinction in mind, manifest professional expertise was stressed as a motivator for diagnostic decision making by both GPs and CAs {16}. This applies to the use of CMRI as NIT prior to ICA in patients with intermediately presumed CCS, for example: *“you just need trained personnel. In other words, a CA with additional CMRI training or […] a radiologist with additional cardiological training who can also operate such equipment” (B08, CA)*. By this logic, the (anticipated) extent of professional expertise may influence whether certain procedures would present as valid options for NIT: *“I used to live in a large city where MPS and stress echocardiography were also available. Here, in this medium-sized city, they are […] available with less expertise, right?” (B02, GP)*. However, the respondents also cross-professionally emphasised that latent personal experience can serve as a motivator for diagnostic decisions, too {17}: *“the medical profession is not only evidence and not only scores and not only CPGs but, basically, also experience, intuition, which cannot be measured” (B09, GP)*. Or “*something like a gut feeling. An assessment on my part: Do I have the impression that this is very likely CAD in need of intervention?*” (B11, CA). As a result, healthcare providers may be more guided by their experience than by evidence. This was indicated with regard to the calculation of pre-test probabilities, for example: *“so, I do not look at the tables now, I have to say, right? But I really do it more according to other criteria and then go according to my experience, according to my feeling, in which direction it goes. […] For me personally, I do not look at the numbers and see from a score of so-and-so percent: then I will go down the path to ICA, right? And I think that is what many of my colleagues do. That it is more of an aid, but not a fixed ritual that you have to follow” (B02, GP)*.

In summary, factors at the provider level may also be diametrically opposed to or in line with guideline recommendations. This may manifest in providers (not/conditionally) using aids such as the calculation of pre-test probabilities. Likewise, providers may (not/conditionally) initiate NIT prior to ICA in patients with intermediately presumed CCS. Due to the ambivalent nature of these viewpoints, provider factors may also aggravate or alleviate influencing factors at other levels. Once again, this may apply in particular to influencing factors at the system level (see sub-chapter “Level 4: Healthcare system”).

#### Level 3: CPGs

**Fig. 5:**
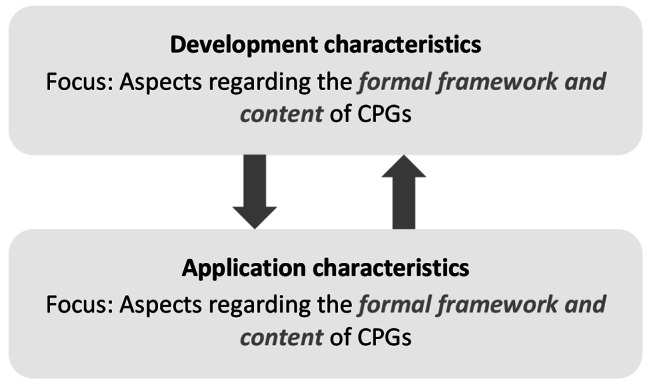
Influencing factors at the CPG level

Factors at the CPG level were rarely coded per interview. As indicated in Fig. 5, the formal and content-related aspects with regard to CPG development and CPG application, were two key reference points here.

With regard to CPG development, GPs and CAs emphasised that by aiming to standardise healthcare CPGs provide a tight formal framework {26}. However, generalisations in CPGs may not reflect the reality of healthcare practice. One respondent described this as a gap between *“what can be objectified” (B11, CA)* and *“what kind of relationship you subjectively built up” (B11, CA)*. This gap was seen as particularly prevalent when it came to the individuality of patient cases: *“CPGs are very popular and are also on the rise. But CPGs are also somehow, sometimes woodcut-like if they are applied too narrowly. […] They are used to try to establish rules for action, but the physician-patient reality is often different – not woodcut-like, but much more varied. […] Individual cases are very, very individual. The symptoms are also very individual in terms of their severity” (B09, GP)*. This may be one reason for formally regarding the application of CPGs as not always mandatory as one GP emphasised {24}. According to the German Medical Association, for example, CPGs “are to be understood as corridors for action and decision-making from which deviations may or even must be made in justified cases. The applicability of a CPG or individual guideline recommendations must be examined for each individual situation according to the principle of indication, consultation, preference determination, and participatory decision-making” [39].

As a result, these formal barriers shape certain content-related barriers at the CPG level. For example, the respondents cross-professionally stressed that CPG content was developed for the sake of formal generalisation but was still characterised by ambiguity {27}. They illustrated this using the example of calculations of pre-test probabilities, which they said included a *“certain grey area. That is, under 15% pre-test probability” (B01, CA)*. This grey area *“is very difficult, right. You already mention this range: that is where it starts, right? Five to fifteen is quite a lot, right? Range” (B02, GP)*. Consequently, it may be possible to apply the calculation of pre-test probabilities loosely. It *“can be put into practice, yes. But it certainly does not have to be. And I think that, in practice, there aren’t many colleagues who only look strictly at these numbers” (B02, GP)*. Furthermore, both GPs and CAs stressed that the application of certain guideline recommendations may be even inexpedient {23}; this was justified, inter alia, with reference to inappropriate consideration of access within the healthcare system, such as the recommendation of CCTA and CMRI as NIT: *“and then I think to myself: what kind of a weak CPG is that, right? Of course, those are great procedures, but they are not even available to me, right?” (B04, GP)*. This problem was attributed, for example, to the sectoral separation in the German healthcare system: *“And for a CA in ambulatory care – like me – who does conservative diagnostics: if I want to have CCTA or CMIR, I have to refer patients to the hospital [i.e. as inpatients]. I cannot just do that/ I cannot just make an outpatient referral.”* (B08, CA).

In summary, these formal and content-related barriers regarding the development and application of CPGs may foster (un)intended deviation from certain recommendations provided through NVL-CAD and ESC-CCS. This may relate to informative aids such as the calculation of pre-test probabilities, but also diagnostic recommendations such as NIT prior to ICA in patients with intermediately presumed CCS. However, the perception of formal and content-related barriers at the CPG level may depend to a large extent on factors at the patient level (see sub-chapter “Level 1: patients”) or the provider level (see sub-chapter “Level 2: healthcare providers”). Additionally, it may be shaped by existing regional healthcare structures (see sub-chapter “Level 4: healthcare system”).

#### Level 4: Healthcare system

**Fig. 6:**
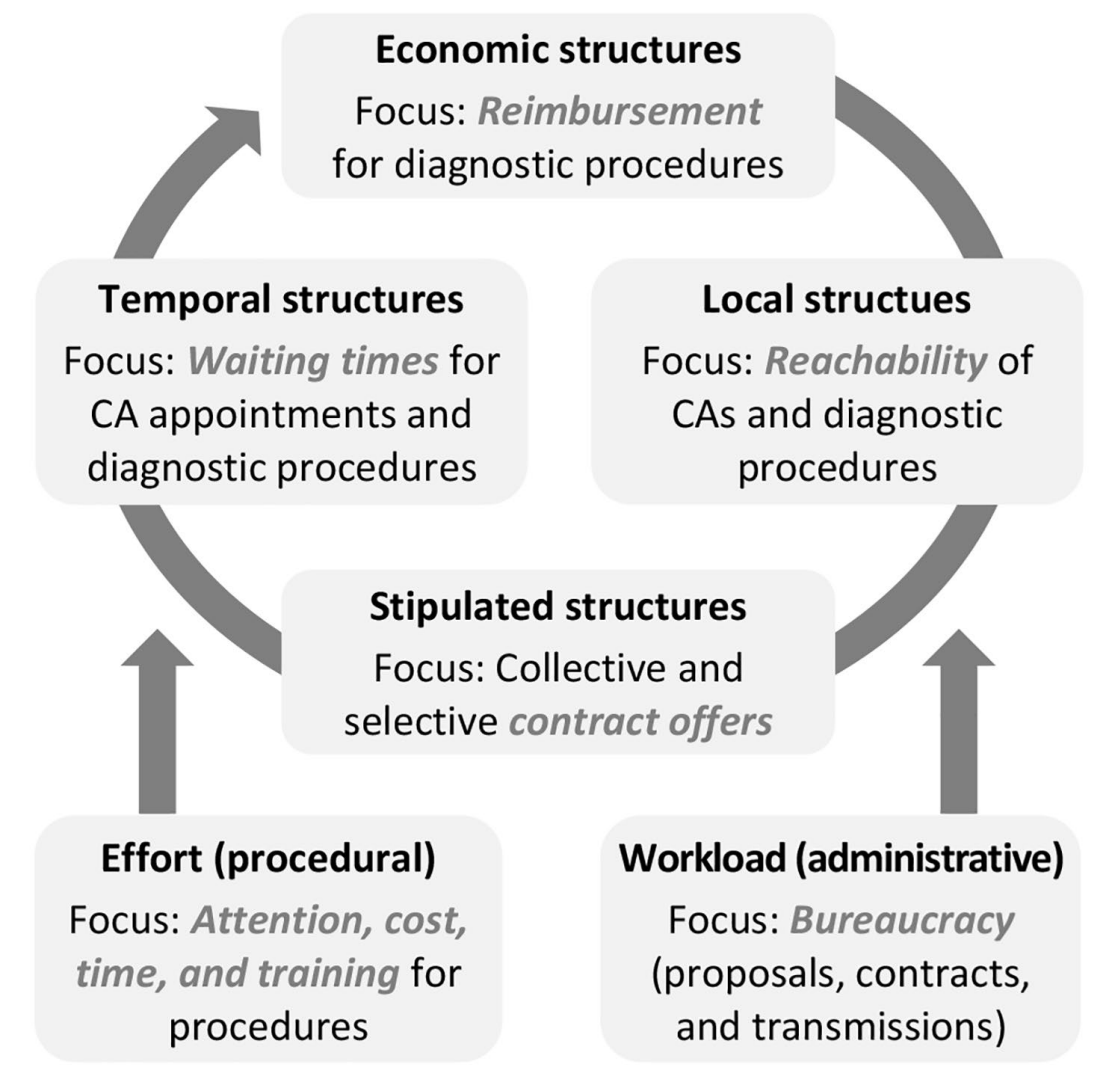
Influencing factors at the system level

Factors at the system level were most frequently coded per interview. According to the respondents’ statements, four intertwined structural aspects acted as pivot points: reachability of providers and services; waiting times; reimbursement through SHI providers; and contract offers. Finally, procedural efforts and administrative workload were expressed as complementing aspects from a processual perspective (see Fig. 6).

Poor reachability of CAs was mentioned from a local perspective by GPs {33}, for example: *“I have worked in several clinics in several towns now and we have the same problem everywhere: there are simply too few CAs. […] In the town where I am based now, for example, there is only one CA – if I recall correctly – with 50,000, 60,000 inhabitants in the town. And in the other towns […] the situation is no different. So basically, I think that is a deficit, right?” (B07, GP)*. As such, GPs perceived waiting times for CA appointments as being too long {35}. Here, *“poor coordination occurs: everyone with thoracic pain shows up at the CA office, without any pre-screening or pre-selection having taken place already” (B12, GP)*. While CAs considered cardiological practices as *“easily accessible and available to patients at every turn”* (B13, CA), the problem of long waiting times was indeed confirmed in CA view: *“The waiting times are more the problem with us, I think. As CAs in ambulatory care. […] And we actually have six months waiting time for routine things. If the GPs want it to go faster, we try. But I think we are often the eye of the needle.”* (B13, CA). As part of standard care, facultative health check-ups were mentioned as an example of a tool that could potentially help overcome this lack of coordination {34}. However, for many patients, these would be only available *“every third year now, right? […] But we are talking about CCS in particular now, right? And I think that more should be done on an outpatient basis” (B07, GP)*. In light of this, selective contracts were cross-professionally perceived as having limited potential as an instrument that could compensate for deficiencies in standard care {34}. They *“involve a certain amount of bureaucracy, which you always shy away from because you don’t have enough time anyway” (B02, GP)*. Despite this, the respondents emphasised that selective contracts may not improve healthcare by sustainable means: *“isolated contracts are made here and there. But that is not the big picture that we need. The big thing is the actual establishment of a primary physician system and the strengthening of the primary physician system” (B09, GP)*. In the end, there are *“many [selective contracts]. It would just be a matter of putting it all together. Then we would probably be dimensions ahead already.”* (B01, CA).

Furthermore, both GPs and CAs stressed a lack of sufficient structure regarding NIT. Stress echocardiography, for example, may not be *“widely available” (B05, GP)* {33}, which may have an impact on waiting times {35}: *“and, unfortunately, until we have free appointments for a stress echocardiography at the moment – especially if we have to do it with medicinal stress […] –/ Then I prefer to send them to CMRI or MPS because that is faster” (B13, CA)*. However, access to alternative procedures for NIT may not always be more sufficient. CCTA *“is still not even widespread” (B03, GP)*, CMRI *“is not even offered in every normal radiology department” (B04, GP)*, and MPS *“is hardly ever offered by nuclear medicine specialists” (B12, GP)* {33}. In addition to this, NIT procedures were characterised as diagnostics with a relatively high workload in terms of attention, training, cost, and/or time, especially from a CA view {30}. As such, one could *“put it this way: I am certainly more likely to be able to catheterise the patient in less time than it takes me to get imaging. Now, that is true for most areas of imaging. For CMRI and CCTA” (B15, CA) {35}*. As this quote illustrates, access to ICA was often described as more widespread and/or faster than NIT by GPs and CAs {33,35}: *“ICA is available everywhere. And it can be done quickly because it has been established for years. I think that is certainly one reason why ICA is done more quickly in hospitals. Because they have the catheter table, but they do not have CMRI and MPS. And then to send the patient home first, to lay it on the GP or to tell them to go to the CA, then they do not get an appointment for those of us in outpatient care. And that is all/ Yes, unpleasant for the patient. And then it is better to just do ICA quickly. Then you have dealt with it […] I think.” (B13, CA)*. However, this may lead to *“a lot of unnecessary invasive diagnostics.”* (B09, GP). From the GPs’ and CAs’ point of view, this combination of a lack of incentives for NIT and disincentives for ICA may also be made worse by economic factors {32}. For example, a lack of SHI reimbursement for NIT conducted using CCTA and CMRI was emphasised as a deficient structure: *“the problem with CMRI and CCTA is that both of these things are actually only offered through hospitals and are inadequately represented in outpatient care – and billing” (B12, GP)*. Furthermore, SHI reimbursement for outpatient stress echocardiography was perceived as inadequate. It would be *“poorly paid, right? And as it is always in medicine: everything that is poorly paid is also poorly done, because of course every physician’s practice is also an economic enterprise to some extent, right? […] And that is why, for example, certain procedures in conservative medicine that are very desirable are simply carried out too rarely because of their poor reimbursement, right?” (B08, CA)*. In turn, financial disincentives were attributed to ICA: one could say that *“too many unnecessary ICAs are being ordered because: these are patients who/ That is right. Now you could say: yes, because the DRGs show that better or show it better than the CPG says” (B12, GP)*.

In summary, these intertwined structures and processes may have two major consequences for guideline adherence. Firstly, they may impede the functional differentiation between primary and secondary care as recommended by NVL-CAD and ESC-CCS. Secondly, they may indicate a potential need to occasionally perform purely diagnostic ICAs without prior NIT, regardless of pre-test probability. In patients with intermediately presumed CCS, this would not correspond to the recommendations of the NVL-CAD and ESC-CCS. However, handling of structural and processual barriers may also depend on factors at the patient and provider levels (see sub-chapters “Level 1: patients” and “Level 2: healthcare providers”).

## Discussion

This interview-based study provides in-depth, context-specific evidence of factors that may potentially influence adherence to CPGs regarding CCS in the ambulatory care sector in Germany. The results can be used to explain observed (non-)adherence. Furthermore, our study could serve as a starting point for the consideration and potential development of measures aimed at optimising guideline adherence. However, the findings still need to be viewed in consideration of the study’s limitations and strengths. These are linked to the epistemological interest, empirical scope, and analytical approach of the study (i.e. internal characteristics), and become apparent when reflecting on its implications for research and practice (i.e. external characteristics).

### Epistemological interest

Regarding the investigation of implementation and uptake of innovations in healthcare practice (e.g. CPGs), Wensing and Grol noted that “most studies report on perceptions of the determinants […]. Research evidence on the actual impact of specific determinants is limited” [40]. However, we argue that both types of studies are needed in order to comprehensively investigate guideline adherence: by exploring factors that influence the uptake of NVL-CAD and ESC-CCS from the perspective of GPs and CAs in Germany, our study provides evidence on exemplary perceptions. In accordance with Weber’s methodological considerations for sociology [37], this illustrates a comprehensive interpretation of reasons that may serve as an example for explaining observed (non-)adherence to CPGs regarding CCS. However, the actual impact of the identified multitude of influencing factors does remain unclear.

Impact-related questions could be answered using studies that analyse the form and extent of (non-) adherence to relevant CPGs. Hereto, analyses conducted in the course of the observational study within ENLIGHT-KHK reveal that, in Germany, patients with intermediately presumed CCS often undergo ICA without first undergoing the recommended NIT (which may indicate a cost savings potential for SHI) [41, 42]. As spatial analyses within the KARDIO study show, there may be some regional variation regarding the use of ICA [12]. Taken together, the two studies indicate the potential impact of the influencing factors identified using the ENLIGHT-KHK interview study, e.g. a patients’ wish for diagnostic confirmation using ICA {6}, and (trans-)regional bottlenecks regarding NIT {32–35}.

However, non-adherence to recommendations provided through guidelines such as NVL-CAD and ESC-CCS can only be assessed to a limited extent: an upstream assumption of the study is that applying quality-assured CPGs (as correctly and comprehensively as possible in healthcare practice) holds the potential to encourage patient-centred and resource-efficient healthcare. This evidence-based understanding of good clinical practice is confronted with a fundamental operationalisation problem regarding guideline adherence as a construct, since there is no standardised approach that allows a reliable assessment of adherence to a respective CPGs (let alone a comparative assessment of adherence to multiple CPGs with potentially diverging recommendations) [43]. Although this problem was not the focus of this study, it should be noted that our findings regarding guideline adherence need to be interpreted with caution, given the potential methodological issues.

### Empirical scope

The respondents’ perceptions are related to their personal characteristics. There are a number of circumstances in which a provider may be more likely to place emphasis on deficiencies in healthcare structures as a barrier to guideline adherence {32–35}, e.g.:

- If the provider needs to arrange referrals within these structures (i.e. profession as a characteristic).
- If the provider’s practice is in a rural or small-town region (i.e. location as a characteristic).
- If the provider’s practice does not collaborate with other healthcare providers (i.e. cooperation as a characteristic).

In order to reflect a broad spectrum of perspectives, the study sample was compiled with a view to variation in profession, practice location, and cooperation. However, due to pragmatic recruitment hurdles (see sub-chapter “Phase A: recruitment” in the Methods chapter), stabilisation of the category system was chosen as a target criterion for the data collection (as opposed to a fully contrastive sample) [44]. This may have resulted in an overrepresentation or underrepresentation of specific perspectives. For example, it was not possible to recruit physicians who worked in a multi-professional medical care centre where there is a separation of practice ownership and healthcare provision activities at the organisational level. Since this allows physicians to work in an employment relationship that places them in direct contact with different medical professions, their views factors that influence guideline adherence may differ from those of self-employed physicians (e.g. with regard to professional collaborations {12} or processual and structural aspects of the healthcare system {30–35}). As such, the focus on self-employed GPs and CAs may have narrowed the empirical scope of the study. However, this remains the predominant form of practice in ambulatory care in Germany [45].

### Analytical approach

Our interpretation of the findings suggests a multitude of factors that influence adherence to NVL-CAD and ESC-CCS. Interdependencies between these factors emerged as a central aspect of this interpretation. For instance, poor availability of equipment in a particular healthcare facility may impede the use of NIT {33}. Consequently, guideline recommendations for the utilisation of NIT may appear inexpedient {23}. At the same time, (trans-)regional bottlenecks with regard to NIT {33} may be aggravated or alleviated by patients’ diagnostic preferences {6} or intra-sectoral and inter-sectoral collaborations {12}. Such interdependencies can occur between influencing factors with different coding frequencies. For instance, only one respondent stressed the ability of patients to travel to healthcare facilities independently as a potential influencing factor {2}, but this may have a crucial influence in terms of ICA in patients with highly presumed CCS: even if collaborative care were ensured {12} and waiting times were appropriate {35} (i.e. two influencing factors that were stressed rather frequently by the respondents), independent travel could prove a deciding factor for the implementation of ICA if the healthcare facilities in question are located too far away from the patient’s home {33}.

As these examples of factorial interdependencies illustrate, an appraisal of major and minor subjects may be biased if it is based solely on coding frequencies. In order to avoid unilateral presentation of frequently coded factors as major subjects, a frequency-based appraisal of code variable values and coding relations was thus integrated as a corrective. This allowed us to compile a profound analysis of the interdependencies between influencing factors. However, in some cases, the code variable values were only marginally dominant, and depictions of coding relations ultimately depend on coding-frequencies. As such, frequency-based appraisals in the course of qualitative analyses serve merely an interpretative crutch for sorting through a complex, ambivalent data corpus, i.e. achieving representativeness through statistical means is not the aim [33].

### Implications for research

According to Nilsen [46], there are three central frameworks that describe general types, classes, or domains of factors for guideline implementation and adherence:

- A theoretical synthesis by Cochrane et al. designed to “expand our understanding of how multiple factors pose barriers to clinical practice” [47].
- An interdisciplinary conceptual framework by Gurses et al. that aims to show “interrelationships among […] major categories of factors […] that influence guideline compliance” [48].
- A heuristic differentiation by Grol et al. that “aims to provide an impression of the range of factors that may be relevant” [49].

Also of note is meta-review by Correa et al., which aims to “explore barriers and facilitators for the implementation of CPGs in different clinical areas of health” [16]. This is based on existing frameworks, including the theoretical synthesis of Cochrane et al. [47].

Overall, the findings of our study are in line with these frameworks. However, our findings are contradicted by Gurses et al. [48], who identified implementation characteristics as a stand-alone main category for processual factors that influence guideline adherence. The authors, however, did not address potential influencing factors at the patient level [46]. Furthermore, none of the frameworks comprehensively explains the interdependencies between the determinants of respective domains, nor do they attempt to assess their relevance for healthcare practice. According to Bach-Mortensen and Verboom, these are central issues for frameworks that are designed to theoretically summarise barriers and facilitators as influencing factors for various outcomes [50]. The findings of our study, on the other hand, provide specific empirical insights into potential dynamics between factors that may influence adherence to NVL-CAD and ESC-CCS with differing thematic and practical relevance. In doing so, the study explains ”how and in what contexts certain factors influence outcomes of interest, and why these factors manifest as they do” [50] based on exemplary primary data.

### Implications for practice

Our findings indicate that GPs and CAs endorse the relevance of adherence to CPGs when caring for patients with suspected CCS but also emphasise the need for maintaining the physicians’ scope: in coherence with the conception provided by the German Association of the Scientific Medical Societies, CPGs were viewed as “possible corridors for action and decision-making” from which deviations may or even should be made in justified cases [51]. This implies that, in case non-adherence to CPGs is observed, measures have to leave room for flexibility in clinical decision making. If such measures are sufficiently sensitive for the clinical perspective, a set of strategically coordinated measures may be useful to effectively promote guideline adherence: Gurses et al. emphasise that, “complying with a particular guideline may require a considerable and unavoidable amount of effort from care providers […]. However, this barrier may be overcome if appropriate and well-designed tools and technologies are made available […] and if the organisational structure is modified […] to facilitate the task” [48]. Behavioural interventions at the system level, for example, aim to indirectly nudge healthcare providers towards the uptake of certain healthcare standards – such as CPGs [52–54]. Their effectiveness is, however, driven by interdependencies between factors at various levels (e.g. patients, healthcare providers, CPGs, and the healthcare system) [48, 50].

For example, it may be possible to alleviate mismatched financial incentivisation of NIT and ICA {32}, a structural barrier to guideline adherence, by revising the SHI reimbursement scheme. This system-level intervention could reduce the number of profitability-based decisions for ICA without prior NIT in patients with intermediately presumed CCS on behalf of healthcare providers {20}. Consequently, recommendations for the use of NIT and ICA in NVL-CAD and ESC-CCS may likewise appear more expedient {23}. However, such a revision of the SHI reimbursement scheme could also result in an unintended financial incentivisation of NIT in patients with low pre-test probabilities {20, 32}.

As a further structural barrier to guideline adherence, the low density of cardiology practices in certain supply regions {33} could be overcome by implementing revisions in demand planning for ambulatory care. If access were to be improved, it could defuse long waiting times for CA appointments {35}. In the long run, this could promote a differentiation between primary and secondary care as recommended in NVL-CAD and ESC-CCS, thus making it seem more expedient {23}. However, the de facto implementation of this functional differentiation may also depend on patients’ preferences in terms of physicians {6}. If patients were to continue to primarily visit CAs when seeking initial clarification, revised demand planning would not have the intended impact.

The applicability of the findings of our study should be investigated within a broader healthcare context, e.g. by conducting a supplementary statistical survey. Moreover, a deeper and more analytical understanding of the interdependencies between factors that influence guideline adherence should be aspired to; this could be achieved by carrying out additional qualitative interviews, for example. These two approaches may help us to further understand whether and to what extent measures are needed to promote adherence to NVL-CAD and ESC-CCS.

## Conclusion

Prima facie, eliminating barriers at the system level may aid with adherence to CPGs with regard to CCS. However, factors at the patient, provider, and CPG levels may foster or impede this process. As such, a set of strategically coordinated measures may be required in order to promote guideline adherence. These measures should take account of the interdependencies between a multitude of barriers and facilitators. However, it should be taken into consideration that, in healthcare practice, guideline recommendations may be deviated from in medically justified cases. The findings of this study provide a starting point for reflecting the necessity and potential form of respective measures.

## Electronic supplementary material

Below is the link to the electronic supplementary material.

Supplementary Material 1

Supplementary Material 2

Supplementary Material 3

Supplementary Material 4

Supplementary Material 5

Supplementary Material 6

## Data Availability

The original data underlying this article cannot be shared publicly due to German data protection regulations. Aggregated datasets used and analysed during the qualitative interview study are available from the corresponding author on reasonable request.

## List of abbreviations

CA: Cardiologist
CAD: Coronary artery disease
CCS: Chronic coronary syndrome
CCTA: Coronary computed tomography angiography
CMRI: Cardiac stress magnetic resonance imaging
CPG: Clinical practice guideline
ESC-CCS: European Society of Cardiology guidelines for the diagnosis and management of chronic coronary syndromes
GP: General practitioner
ICA: Invasive coronary angiography
MPS: Myocardial perfusion scintigraphy
NIT: Non-invasive ischemia testing
NVL-CAD: German national disease management guideline on chronic coronary disease (German title: Nationale VersorgungsLeitlinie “Chronische KHK”)
SHI: Statutory health insurance

## Declarations

### Authors information

**Marie Naumann** At the time of the study, MN was working as a junior research associate at the Cologne Institute for Health Economics and Clinical Epidemiology (University Hospital of Cologne). With a Masters’ degree in Sociology, MN possesses extensive professional expertise in qualitative social research. Furthermore, MN possesses fundamental practical experience of working on empirical projects relating to various societal topics within the field of qualitative social research.

**Simon Robin Scharfenberg** At the time of the study, SSC was working as a research assistant at the Cologne Institute for Health Economics and Clinical Epidemiology (University Hospital of Cologne).

**Yana Seleznova** At the time of the study, YS was working as a junior research associate at the Cologne Institute for Health Economics and Clinical Epidemiology (University Hospital of Cologne).

**Bastian Wein** At the time of the study, BW was working as a project manager at the Contilia Heart and Vascular Center (Elisabeth-Hospital Essen) and a senior physician at the Cardiovascular Center Oberallgaeu-Kempten.

**Oliver Bruder** At the time of the study, OB was working as a chief physician at the Contilia Heart and Vascular Center (Elisabeth-Hospital Essen) and a lecturer of medicine at the Ruhr University Bochum.

**Stephanie Stock** At the time of the study, SST was working as the Deputy Director of the Cologne Institute for Health Economics and Clinical Epidemiology (University Hospital of Cologne).

**Dusan Simic** At the time of the study, DS was working as a senior research associate at the Cologne Institute for Health Economics and Clinical Epidemiology (University Hospital of Cologne).

**Benjamin Scheckel** At the time of the study, BS was working as a junior research associate at the Cologne Institute for Health Economics and Clinical Epidemiology (University Hospital of Cologne).

**Dirk Müller** At the time of the study, DM was working as a senior research associate at the Cologne Institute for Health Economics and Clinical Epidemiology (University Hospital of Cologne). With a PhD in Health Economics, DM has led several research projects in the field of health services research. He possesses extensive professional expertise regarding methodologies from the quantitative and qualitative paradigm, and his comprehensive practical experience has been gained in the areas of both healthcare-related basic research and applied healthcare research.
