# Supplementary material for: Factors influencing adherence to clinical practice guidelines in patients with suspected chronic coronary syndrome: a qualitative interview study in the ambulatory care sector in Germany
Source: BMC Health Serv Res. 2023 Jun 20;23:655. doi: 10.1186/s12913-023-09587-1 (PMC10283181; doi:10.1186/s12913-023-09587-1)
Supplement: Supplementary file 1 — Supplementary Material 1 [file 12913_2023_9587_MOESM1_ESM.docx]

COREQ Checklist

Formal adaption from: Tong A, Sainsbury P, Craig J. Consolidated criteria for reporting qualitative research (COREQ): a 32-item checklist for interviews and focus groups. *Int J Qual Health Care*. 2007 Sep 16;19(6):349–57.

| **Topic** | **No.** | **Guide questions** | **Reported in the following (sub-)chapter:** |
| --- | --- | --- | --- |
| **Domain 1: Research team and reflexivity** | | | |
| *Personal characteristics* | | | |
| Interviewer/facilitator | 1 | Which author/s conducted the interview or focus group? | - Declarations 🡪 Author contribution |
| Credentials | 2 | What were the researcher’s credentials? E.g., PhD, MD | - Declarations 🡪 Author information (optional) |
| Occupation | 3 | What was their occupation at the time of the study? | - Declarations 🡪 Author information (optional) |
| Gender | 4 | Was the researcher male or female? | - N/A |
| Experience and training | 5 | What experience or training did the researcher have? | - Declarations 🡪 Author information (optional) |
| *Relationship with participants* | | | |
| Relationship established | 6 | Was a relationship established prior to study commencement? | - Methods 🡪 Design and setting 🡪 Phase B: Data collection |
| Participant knowledge of the interviewer | 7 | What did the participants know about the researcher? E.g., personal goals, reasons for doing the research | - Methods 🡪 Design and setting 🡪 Phase B: Data collection |
| Interviewer characteristics | 8 | What characteristics were reported about the interviewer/facilitator? E.g., Bias, assumptions, reasons, and interests in the research topic | - Methods 🡪 Design and setting 🡪 Phase B: Data collection |
| **Domain 2: Study design** | | | |
| *Theoretical framework* | | | |
| Methodological orientation and theory | 9 | What methodological orientation was stated to underpin the study? E.g., grounded theory, discourse analysis, ethnography, phenomenology, content analysis | - Methods 🡪 Design and setting 🡪 Phase C: Data analysis |
| *Participant selection* | | | |
| Sampling | 10 | How were participants selected? E.g., purposive, convenience, consecutive, snowball | - Methods 🡪 Design and setting 🡪 Phase A: Recruitment |
| Method of approach | 11 | How were participants approached? E.g., face-to-face, telephone, mail, email | - Methods 🡪 Design and setting 🡪 Phase B: Data collection |
| Sample size | 12 | How many participants were in the study? | - Results 🡪 Description of findings 🡪 Study sample |
| Non-participation | 13 | How many people refused to participate or dropped out? Reasons? | - Results 🡪 Description of findings 🡪 Study sample |
| *Setting* | | | |
| Setting of data collection | 14 | Where was the data collected? E.g., home, clinic, workplace | - Declarations 🡪 Author contribution |
| Presence of non-participants | 15 | Was anyone else present besides the participants and researchers? | - Declarations 🡪 Author contribution |
| Description of sample | 16 | What are the important characteristics of the sample? E.g., demographic data, date | - Results 🡪 Description of findings 🡪 Study sample |

| *Data collection* | | | |
| --- | --- | --- | --- |
| Interview guide | 17 | Were questions, prompts, guides provided by the authors? Was it pilot tested? | - Results 🡪 Description of findings 🡪 Study sample |
| Repeat interviews | 18 | Were repeat interviews carried out? If yes, how many? | - Results 🡪 Description of findings 🡪 Study sample |
| Audio/visual recording | 19 | Did the research use audio or visual recording to collect the data? | - Methods 🡪 Design and setting 🡪 Phase B: Data collection |
| Field notes | 20 | Were field notes made during and/or after the interview or focus group? | - Declarations 🡪 Author contribution |
| Duration | 21 | What was the duration of the interviews or focus group? | - Results 🡪 Description of findings 🡪 Study sample |
| Data saturation | 22 | Was data saturation discussed? | - Discussion 🡪 Empirical scope |
| Transcripts returned | 23 | Were transcripts returned to participants for comment and/or correction? | - Methods 🡪 Design and setting 🡪 Phase B: Data collection - Results 🡪 Description of findings 🡪 Study sample |
| **Domain 3: Analysis and findings** | | | |
| *Data analysis* | | | |
| Number of data coders | 24 | How many data coders coded the data? | - Declarations 🡪 Author contribution |
| Description of the coding tree | 25 | Did authors provide a description of the coding tree? | - Results 🡪 Description of findings 🡪 Category system |
| Derivation of themes | 26 | Were themes identified in advance or derived from the data? | - Methods 🡪 Design and setting 🡪 Phase C: Data analysis |
| Software | 27 | What software, if applicable, was used to manage the data? | - Methods 🡪 Design and setting 🡪 Phase C: Data analysis |
| Participant checking | 28 | Did participants provide feedback on the findings? | - Methods 🡪 Design and setting 🡪 Phase C: Data analysis |
| *Reporting* | | | |
| Quotations presented | 29 | Were participant quotations presented to illustrate the themes/findings? Was each quotation identified? E.g., participant number | - Results 🡪 Interpretation of findings 🡪 All sub-chapters |
| Data findings consistent | 30 | Was there consistency between the data presented and the findings? | - Results 🡪 All sub-chapters - Discussion 🡪 All sub-chapters |
| Clarity of major themes | 31 | Were major themes clearly presented in the findings? | - Results 🡪 Interpretation of findings 🡪 All sub-chapters - Discussion 🡪 Analytical approach |
| Clarity of minor themes | 32 | Is there a description of diverse cases or discussion of minor themes? | - Results 🡪 Interpretation of findings 🡪 All sub-chapters - Discussion 🡪 Analytical approach |
