# Supplementary material for: Factors influencing adherence to clinical practice guidelines in patients with suspected chronic coronary syndrome: a qualitative interview study in the ambulatory care sector in Germany
Source: BMC Health Serv Res. 2023 Jun 20;23:655. doi: 10.1186/s12913-023-09587-1 (PMC10283181; doi:10.1186/s12913-023-09587-1)
Supplement: Supplementary file 2 — Supplementary Material 2 [file 12913_2023_9587_MOESM2_ESM.docx]

Interview guide

In the course of the semi-structured interviews, a **profession-specific interview guide** was used. It consists mainly of **open-ended questions** that are occasionally paired with close-ended questions. The general structure of the interview guide is determined by consecutive topics. However, jumping from one topic to another due to respective interview dynamics was possible.

Based on preparatory steps within the data analysis phase, the **initial interview guide was slightly updated due to redundancies and specification needs** after the seventh interview (first interview phase). The updated interview guide was utilised for the remaining eight interviews (second interview phase).

**Functions of the interview guide** (grey box at the bottom of each topic) will be explained in the following. Furthermore, **reasons for respective modifications** of the interview guide will be described (red exclamation marks and additional red box at the bottom of each topic). **Profession-specific questions** will be highlighted as well.

# Interview guide for the first interview phase (n=7)

| **TOPIC 1: Typical healthcare process for suspected CCS** | |
| --- | --- |
| **Guiding question** | |
| To start with, could you describe how you typically tend to suspected CCS? | |
| **Explanation of guiding question** | |
| It would be interesting to look at individual sub-processes (e.g. history, physical examination, diagnostics, etc.) or specific sub-aspects (e.g. regarding symptoms, referral decisions, etc.). | |
| **Specifying questions** | |
| How does healthcare for de novo CCS differ from healthcare for known CCS? | |
| If you had to determine which points are of particular importance for your decisions: what would they be? | |
| When/with regard to which symptoms do you refer patients to cardiologists, hospitals, or clinics and why? (🡪 GPs!) | When/with regard to which symptoms do you refer patients to hospitals or clinics and why?  (🡪 CAs!) |
| Who are the central stakeholders in the healthcare process?/What is the role of ...?/What competence lies with ...? | |
| **Functional explanation:**   - TOPIC 1 has an orienting function for the entire interview, especially for TOPIC 2. - The guiding question, thereby, serves to draw a reference frame. This may already provide indications for healthcare challenges with regard to suspected CCS (TOPIC 2). - The explanation of the guiding question alleviates comprehension problems on behalf of the respondent considering the highly open-ended guiding question. - The specifying questions serve to (i) capture a problem in a decidedly clinical perspective (question regarding de novo CCS versus known CCS), (ii) force a statement on path-logical and time-logical aspects (questions regarding important decision points and referral logic, respectively), and (iii) identify and hierarchise responsibilities within healthcare for suspected CCS (questions regarding stakeholders, role, and competence). | |

| **TOPIC 2: Healthcare challenges with regard to suspected CCS** |
| --- |
| **Guiding question** |
| What challenges do you face during healthcare for suspected CCS? |
| **Explanation of guiding question** |
| You are welcome to refer to individual sub-processes and sub-aspects again. |
| **Specifying questions** |
| Is ... an individual or structural phenomenon? |
| Can you determine where the cause of ... lies?/What do you think is the cause? |
| What is your access to non-invasive and invasive testing? |
| What is your availability and waiting time for appointments for non-invasive and invasive testing? |
| **Functional explanation:**   - TOPIC 2 has a specifying function regarding problems during healthcare for suspected CCS. Redundancies after TOPIC 1 should be avoided. - The guiding question, thereby, serves to identify a perception on problems that have a central influence on physicians’ decision-making practice. - The explanation of the guiding question alleviates comprehension problems on behalf of the respondent considering the highly open-ended guiding question. - The specifying questions serve to (i) map the reach of a problem (question regarding individual versus structural phenomenon), (ii) allow for a pointed problem expression (question regarding cause), and (iii) especially learn about the personal healthcare situation of the respondent (questions regarding non-invasive versus invasive testing). |

| **TOPIC 3: Personal handling of CPGs** | |
| --- | --- |
| **Guiding question** | |
| What would you say: how do CPGs regarding CCS come into play in your daily work? | |
| **Explanation of guiding question** | |
| Central: NVL on CAD and ESC on CCS. | |
| **Specifying questions** | |
| How often and in what form do you use respective CPGs regarding healthcare for suspected CCS? | |
| In your opinion: what are the advantages/disadvantages of using respective CPGs regarding healthcare for suspected CCS? | |
| In your opinion: what role/relevance do aids such as the Marburg Heart Score (MHS) have for the healthcare process? (🡪GPs!) | In your opinion: what role/relevance do aids such as the calculation of the pre-test probability have for the healthcare process? (🡪 CAs!) |
| **Functional explanation:**   - TOPIC 3 has the lowest thematic relevance, but functions as a transition to TOPIC 4. Therefore: important but keep it short. - The guiding question, thereby, serves to encourage a comment on the specific role and relevance of respective CPGs. Prerequisite: CPGs must be known by the respondent. - The explanation of the guiding question can be used to draw the focus to relevant CPGs. - The specifying questions serve to (i) test if respondents know relevant CPGs which significantly determines the further course of the interview (question regarding form of use), and (ii) capture the assessment of functionality of CPGs regarding potentials, limitations, and optimisation (questions regarding (dis)advantages and aiding instruments, respectively). | |

| **TOPIC 4: Pathways towards guideline adherence** | |
| --- | --- |
| **Guiding question** | |
| In your opinion: how could guideline-orientated healthcare for patients with suspected CCS be implemented in Germany? | |
| **Explanation of guiding question** | |
| In other words: which healthcare processes and structures would have to change? | |
| **Specifying questions** | |
| What waiting times do you consider acceptable for an appointment for non-invasive or invasive testing in case of suspected CCS? (🡪 GPs!) | What waiting times do you consider practical for an appointment for non-invasive or invasive testing in case of suspected CCS? (🡪 CAs!) |
| How do you rate the usefulness of previous support services for healthcare for suspected CCS? | |
| What support services would you like to see for healthcare for suspected CCS? | |
| Have you or your practice ever participated in a special care contract with a statutory health insurance provider (so-called selective contracts)? | |
| In your opinion: what contribution could selective contracts provide with regard to healthcare for suspected CCS? | |
| In your opinion: which services should be covered by a selective contract focused on healthcare for suspected CCS? | |
| How do you estimate the effort with regard to enrolling patients in a selective contract? | |
| **Functional explanation:**   - TOPIC 4 has a specifying function regarding possibilities of improvement with regard to guideline-adherent healthcare. - The guiding question, thereby, serves to record (in)direct indications for the outline of a selective contract promoting guideline adherence. This is a working package in the course of ENLIGHT-KHK. Prerequisite: respondent has a positive-critical perspective on CPGs. - The explanation of the guiding question alleviates comprehension problems on behalf of the respondent considering the highly open-ended guiding question. - The specifying questions serve to (i) capture acceptable/practical waiting times for appointments for (non-)invasive testing (questions regarding non-invasive versus invasive testing), (ii) identify previous/needed aiding instruments for guideline-adherent healthcare for suspected CCS (questions regarding previous support services and future support services, respectively), (iii) grasp the respondents experience with selective contracts which significantly determines the further course of the interview (question regarding selective contract participation), (iv) grasp the respondents perspective on a CCS focused selective contract with regard to expected services (questions regarding contribution of selective contracts and services of selective contracts, respectively), and (v) estimate the bureaucratic/organisational workload with regard to enrolling patients in the corresponding selective contracts (question regarding effort of patient enrolment). | |

# Interview guide for the second interview phase (n=8)

| **TOPIC 1: Typical healthcare process for suspected CCS** | |
| --- | --- |
| **Guiding question** | |
| To start with, could you describe how you typically tend to suspected CCS? | |
| **Explanation of guiding question** | |
| It would be interesting to look at sub-processes (e.g. history, physical examination, diagnostics, etc.) or sub-aspects (e.g. regarding symptoms, referral decisions, etc.). | |
| **Specifying questions** | |
| How does healthcare in case of first-time symptoms (de novo CCS) differ from healthcare in case of progressing symptoms (known CCS)? **!** | |
| If you had to determine which points are of particular importance for your decisions: what would they be? | |
| When/with regard to which symptoms do you refer patients to cardiologists, hospitals, or clinics and why? (🡪 GPs!) | When/with regard to which symptoms do you refer patients to hospitals or clinics and why?  (🡪 CAs!) |
| Who are the central stakeholders in the healthcare process?/What is the role of ...?/What competence lies with ...? | |
| **Functional explanation:**   - TOPIC 1 has an orienting function for the entire interview, especially for TOPIC 2. - The guiding question, thereby, serves to draw a reference frame. This may already provide indications for healthcare challenges with regard to suspected CCS (TOPIC 2). - The explanation of the guiding question alleviates comprehension problems on behalf of the respondent considering the highly open-ended guiding question. - The specifying questions serve to (i) capture a problem in a decidedly clinical perspective (question regarding de novo CCS versus known CCS), (ii) force a statement on path-logical and time-logical aspects (questions regarding important decision points and referral logic, respectively), and (iii) identify and hierarchise responsibilities within healthcare for suspected CCS (questions regarding stakeholders, role, and competence). | |
| **Explanation regarding modifications:**   - The specifying question regarding de novo CCS versus known CCS was discarded in favour of a version that is closer to everyday language. | |

| **TOPIC 2: Healthcare challenges with regard to suspected CCS** | |
| --- | --- |
| **Guiding question** | |
| What challenges do you face during healthcare for suspected CCS? | |
| **Explanation of guiding question** | |
| You are welcome to refer to sub-processes and sub-aspects again. | |
| **Specifying questions** | |
| Is ... an individual or structural phenomenon? | |
| Can you determine where the cause of ... lies?/What do you think is the cause? | |
| What is your access to non-invasive and invasive testing? | |
| What is your availability and waiting time for appointments for non-invasive and invasive testing? | |
| What waiting times do you consider acceptable for an appointment for non-invasive or invasive testing in case of suspected CCS? (🡪 GPs!) **!** | What waiting times do you consider practical for an appointment for non-invasive or invasive testing in case of suspected CCS? (🡪 CAs!) **!** |
| **Functional explanation:**   - TOPIC 2 has a specifying function regarding problems during healthcare for suspected CCS. Redundancies after TOPIC 1 should be avoided. - The guiding question, thereby, serves to identify a perception on problems that have a central influence on physicians’ decision-making practice. - The explanation of the guiding question alleviates comprehension problems on behalf of the respondent considering the highly open-ended guiding question. - The specifying questions serve to (i) map the reach of a problem (question regarding individual versus structural phenomenon), (ii) allow for a pointed problem expression (question regarding cause), and (iii) especially learn about the personal healthcare situation of the respondent (questions regarding non-invasive versus invasive testing) | |
| **Explanation regarding modifications:**   - The specifying question regarding acceptable/practical waiting times for appointments for (non-)invasive testing was pulled forward. This was done to provide a direct connection between specifying questions regarding (non-)invasive testing. | |

| **TOPIC 3: Pathways towards guideline adherence** **!** |
| --- |
| **Guiding question** |
| Which healthcare processes and structures would have to change to support you in healthcare for suspected CCS? **!** |
| **Explanation of guiding question** |
| Here, changes with regard to the challenges you already mentioned are of special interest. **!** |
| **Specifying questions** |
| In your opinion: how could guideline-orientated healthcare for patients with suspected CCS be implemented in Germany? **!** |
| How do you rate the usefulness of previous support services for healthcare for suspected CCS? |
| What support services would you like to see for healthcare for suspected CCS? |
| In your opinion: which services should be covered by a selective contract focused on healthcare for suspected CCS? **!** |
| How do you estimate the effort with regard to enrolling patients in a selective contract? **!** |
| **Functional explanation:**   - TOPIC 3 has a specifying function regarding possibilities of improvement with regard to guideline-adherent healthcare. - The guiding question, thereby, serves to record (in)direct indications for the outline of a selective contract promoting guideline adherence. This was a working package in the course of ENLIGHT-KHK. Prerequisite: respondent has a positive-critical perspective on CPGs. - The explanation of the guiding question alleviates comprehension problems on behalf of the respondent considering the highly open-ended guiding question. - The specifying questions serve to (i) capture a decisive perspective on possibilities of improvement regarding guideline-adherent healthcare for suspected CCS, (ii) identify previous/needed aiding instruments for guideline-adherent healthcare for suspected CCS (questions regarding previous support services and future support services, respectively), (iii) grasp the respondents perspective on a CCS focused selective contract with regard to expected services and experienced efforts (question regarding selective contract). |
| **Explanation regarding modifications:**   - TOPIC 3 depicts TOPIC 4 in the interview guide of the first interview phase. TOPIC 3 of the initial version of the interview guide turned out to be obsolete. - Additionally, the guiding question was discarded in favour of a less open-ended one because the initial version did not lead to answers regarding concrete possibilities of improvement. Simultaneously, the original focus on guideline-adherent healthcare in patients with suspected CCS was converted into a specifying question. Thus, answers would not be initially drawn to a critical perspective on the notion of guideline adherence. Namely, this could lead away from a perspective on possibilities of improvement for healthcare in patients with suspected CCS. - Furthermore, the explanation of the guiding question was modified to match the new guiding question whilst also drawing a connection to TOPIC 2. - Eventually, some specifying questions regarding selective contracts turned out to be obsolete during the first interview phase. Thus, they were discarded. Finally, the remaining specifying questions were pulled together as they turned out to linked thematically. |

| **TOPIC 4: Assessment of the selective contract outline** **!** | |
| --- | --- |
| **Guiding question** | |
| What do you think of this approach: do you think it could support you in view of the challenges and possibilities of improvement mentioned regarding healthcare for suspected CCS? | |
| **Explanation of guiding question** | |
| Briefly explanation of the current start of the art of the outline for selective contract that is developed by stakeholders within ENLIGHT-KHK (incl. the notion of implementing an IT-tool that promotes guideline-adherent healthcare). | |
| **Specifying questions** | |
| How do you assess the role of IT-tools in your healthcare practice? | |
| Do you find IT-tools fundamentally useful as a companion in healthcare practice? | |
| Which functions would you like to have in an IT-tool to support you in a guideline-oriented healthcare for suspected CCS? | |
| How would a respective selective contract have to be designed to attract you to participate? | |
| How could the enrolment of patients in a respective selective contract go smoothly from your point of view (keyword: bureaucracy)? | |
| How should the cooperation with cardiologists look like from your point of view (keyword: allocation of appointments)?  (🡪 GPs!) | How should the cooperation with GPs look like from your point of view (keyword: allocation of appointments)?  (🡪 CAs!) |
| **Functional explanation:**   - TOPIC 4 has a specifying function regarding potential improvements for the selective contract outline as initially developed by stakeholders within ENLIGHT-KHK. This outline includes the idea of implementing an IT-tool that promotes guideline-adherent healthcare. - The guiding question, thereby, serves to record (in)direct indications for necessary changes of the developed selective contract outline as well as the contemplated IT-tool. - The explanation of the guiding question is mandatory. - Against the background of the developed selective contract outline, the specifying questions serve to (i) record the basic attitude towards IT-tools in assessment practice (questions regarding role of IT-tools and usefulness of IT-tools, respectively), (ii) focus on the needed functionalities of an IT-tool (question regarding functions of IT-tools), and (iii) focus on the needed formal framework (questions regarding selective contract design, enrolment of patients, and cooperation, respectively). | |
| **Explanation regarding modifications:**   - TOPIC 4 was introduced in the second interview phase due to further project developments of the outline of a selective contract within the context of ENLIGHT-KHK. | |
