# Supplementary material for: Factors influencing adherence to clinical practice guidelines in patients with suspected chronic coronary syndrome: a qualitative interview study in the ambulatory care sector in Germany
Source: BMC Health Serv Res. 2023 Jun 20;23:655. doi: 10.1186/s12913-023-09587-1 (PMC10283181; doi:10.1186/s12913-023-09587-1)
Supplement: Supplementary file 3 — Supplementary Material 3 [file 12913_2023_9587_MOESM3_ESM.docx]

Inter-coder agreement

The monitoring of inter-coder agreement made visible **to which extend the independently coded sub-categories of both coders overlap** in terms of main category allocation, i.e. the patient, provider, CPG, and system levels. Three cases were differentiated:

| **Case 1: Clear overlap**  Both coders created a semantically similar sub-category at the same level with code segments overlapping at least 90%. For example: “Patients’ mentality” (Coder 1) versus “Personality of patients” (Coder 2) as sub-categories under the main category “Patient level” with a 95% overlap of code segments. |
| --- |
| **Case 2: Conditional overlap**  Both coders created a semantically similar sub-category at the same level with code segments overlapping less than 90%. However, they were identified in the same paragraph of an interview. For example: “Patients’ mentality” (Coder 1) versus “Personality of patients” (Coder 2) as sub-categories under the main category “Patient level” with a 20% overlap of code segments. |
| **Case 3: No overlap**  One coder created a sub-category that was not created by the other coder at all or that was semantically different from a sub-category created by the other coder (regardless of possible overlapping of code segments or allocations at the same level). For example: “Patients’ mentality” (Coder 1) as a sub-category under the main category “Patient level” versus “Healthcare providers perception of patients” (Coder 2) as sub-category under the main category “Healthcare provider level” with 95% overlap of code segments. |

Since the data corpus consists of non-standardised data, inter-coder agreement was overall low and non-increasing. This is shown in an aggregated overview of the monitoring process (see section I on page 2 in this file). If only clear overlaps are counted (=matches in strict perspective), the percentual **mean inter-coder agreement amounts to 13%.** If conditional overlaps are counted as agreements, too, it amounts to **48%, respectively** (=matches in less strict perspective).

Based on moving means (period value two), a **trend towards increasing inter-coder agreement did not emerge**. Inter-coder agreement monitoring, however, did not have a verifying function for the creation of sub-categories in the first place. As a detailed overview of the results of the monitoring depicts, monitoring of inter-coder agreement rather enabled **mapping of difficulties** regarding the formulation of sub-categories (see section II on page 3 et seqq. in this file). Thus, it provided a **tangible starting point for a focused discussion** between the two coders that took place as a **repeated collaborative review of the evolving category system**.

# Aggregated overview

Mean value of percentual matching across interviews based on clear overlaps: **13%**

Mean value of percentual matching across interviews based on clear and conditional overlaps: **48%**

# Detailed overview

## Interview 1

| **Overlaps of code segments in sum** | | | | | | |
| --- | --- | --- | --- | --- | --- | --- |
| **Allocated main category** | **Matching**  **(strict)** | **Non-Matching**  **(strict)** | **Matching in % (strict)** | **Matching**  **(less strict)** | **Non-Matching**  **(less strict)** | **Matching in % (less strict)** |
| **Patient level** | 0 | 3 | 0% | 0 | 3 | 0% |
| **Healthcare provider level** | 0 | 5 | 0% | 2 | 3 | 40% |
| **CPG level** | 1 | 5 | 17% | 4 | 2 | 67% |
| **Healthcare system level** | 0 | 0 | - | 0 | 0 | - |
| **<Total>** | **1** | **13** | **7%** | **6** | **8** | **43%** |
| **Overlaps of code segments per paragraph** | | | | | | |
| **Allocated main category** | **CODER 1** | | **CODER 2** | | **Paragraph** | |
| **Healthcare provider level** | 0 | | 1 | | **2** | |
| **Healthcare provider level** | 1 | | 0 | | **4** | |
| **CPG level** | 1 | | 0 | | **4** | |
| **CPG level** | 0 | | 1 | | **4** | |
| **CPG level** | 0 | | 1 | | **4** | |
| **Healthcare provider level** | 0 | | 1 | | **10** | |
| **Healthcare provider level** | 1 | | 0 | | **10** | |
| **Patient level** | 0 | | 1 | | **12** | |
| **Patient level** | 0 | | 1 | | **16** | |
| **CPG level** | 1 | | 0 | | **16** | |
| **CPG level** | 1 | | 1 | | **18** | |
| **Patient level** | 0 | | 1 | | **20** | |
| **Healthcare provider level** | 0 | | 1 | | **22** | |
| **CPG level** | 0 | | 1 | | **22** | |

## Interview 2

| **Overlaps of code segments in sum** | | | | | | |
| --- | --- | --- | --- | --- | --- | --- |
| **Allocated main category** | **Matching**  **(strict)** | **Non-Matching**  **(strict)** | **Matching in % (strict)** | **Matching**  **(less strict)** | **Non-Matching**  **(less strict)** | **Matching in % (less strict)** |
| **Patient level** | 0 | 2 | 0% | 2 | 0 | 100% |
| **Healthcare provider level** | 0 | 9 | 0% | 7 | 2 | 78% |
| **CPG level** | 1 | 2 | 33% | 1 | 2 | 33% |
| **Healthcare system level** | 1 | 6 | 14% | 3 | 4 | 43% |
| **<Total>** | **2** | **19** | **10%** | **13** | **8** | **62%** |
| **Overlaps of code segments per paragraph** | | | | | | |
| **Allocated main category** | **CODER 1** | | **CODER 2** | | **Paragraph** | |
| **Healthcare provider level** | 0 | | 1 | | **2** | |
| **Healthcare provider level** | 1 | | 0 | | **2** | |
| **Healthcare provider level** | 1 | | 0 | | **6** | |
| **Healthcare provider level** | 0 | | 1 | | **6** | |
| **CPG level** | 1 | | 0 | | **6** | |
| **Healthcare provider level** | 0 | | 1 | | **10** | |
| **CPG level** | 1 | | 0 | | **10** | |
| **CPG level** | 1 | | 1 | | **16** | |
| **Healthcare provider level** | 0 | | 1 | | **18** | |
| **Healthcare provider level** | 0 | | 1 | | **18** | |
| **Healthcare provider level** | 1 | | 0 | | **18** | |
| **Healthcare system level** | 1 | | 1 | | **18** | |
| **Patient level** | 1 | | 0 | | **20** | |
| **Healthcare system level** | 1 | | 0 | | **20** | |
| **Patient level** | 0 | | 1 | | **20** | |
| **Healthcare system level** | 1 | | 0 | | **32** | |
| **Healthcare system level** | 1 | | 0 | | **32** | |
| **Healthcare system level** | 0 | | 1 | | **40** | |
| **Healthcare system level** | 1 | | 0 | | **40** | |
| **Healthcare system level** | 0 | | 1 | | **44** | |
| **Healthcare provider level** | 0 | | 1 | | **46** | |

## Interview 3

| **Overlaps of code segments in sum** | | | | | | |
| --- | --- | --- | --- | --- | --- | --- |
| **Allocated main category** | **Matching**  **(strict)** | **Non-Matching**  **(strict)** | **Matching in % (strict)** | **Matching**  **(less strict)** | **Non-Matching**  **(less strict)** | **Matching in % (less strict)** |
| **Patient level** | 0 | 4 | 0% | 0 | 4 | 0% |
| **Healthcare provider level** | 0 | 4 | 0% | 0 | 4 | 0% |
| **CPG level** | 1 | 1 | 50% | 1 | 1 | 50% |
| **Healthcare system level** | 1 | 1 | 25% | 3 | 3 | 75% |
| **<Total>** | **2** | **10** | **14%** | **4** | **12** | **29%** |
| **Overlaps of code segments per paragraph** | | | | | | |
| **Allocated main category** | **CODER 1** | | **CODER 2** | | **Paragraph** | |
| **Healthcare provider level** | 1 | | 0 | | **2** | |
| **Patient level** | 0 | | 1 | | **8** | |
| **Patient level** | 0 | | 1 | | **8** | |
| **Patient level** | 0 | | 1 | | **8** | |
| **CPG level** | 0 | | 1 | | **8** | |
| **Healthcare provider level** | 0 | | 1 | | **8** | |
| **Healthcare provider level** | 0 | | 1 | | **12** | |
| **Healthcare system level** | 1 | | 0 | | **12** | |
| **Healthcare system level** | 0 | | 1 | | **12** | |
| **CPG level** | 1 | | 1 | | **16** | |
| **Patient level** | 0 | | 1 | | **36** | |
| **Healthcare provider level** | 0 | | 1 | | **36** | |
| **Healthcare system level** | 1 | | 1 | | **40** | |
| **Healthcare system level** | 0 | | 1 | | **42** | |

## Interview 4

| **Overlaps of code segments in sum** | | | | | | |
| --- | --- | --- | --- | --- | --- | --- |
| **Allocated main category** | **Matching**  **(strict)** | **Non-Matching**  **(strict)** | **Matching in % (strict)** | **Matching**  **(less strict)** | **Non-Matching**  **(less strict)** | **Matching in % (less strict)** |
| **Patient level** | 0 | 0 | - | 0 | 0 | - |
| **Healthcare provider level** | 0 | 19 | 0% | 3 | 16 | 16% |
| **CPG level** | 0 | 1 | 0% | 0 | 1 | 0% |
| **Healthcare system level** | 1 | 27 | 4% | 9 | 19 | 32% |
| **<Total>** | **1** | **47** | **2%** | **12** | **36** | **25%** |
| **Overlaps of code segments per paragraph** | | | | | | |
| **Allocated main category** | **CODER 1** | | **CODER 2** | | **Paragraph** | |
| **Healthcare provider level** | 1 | | 0 | | **2** | |
| **Healthcare provider level** | 0 | | 1 | | **4** | |
| **Healthcare provider level** | 0 | | 1 | | **6** | |
| **Healthcare system level** | 0 | | 1 | | **8** | |
| **Healthcare provider level** | 1 | | 0 | | **8** | |
| **Healthcare system level** | 0 | | 1 | | **10** | |
| **Healthcare provider level** | 1 | | 0 | | **10** | |
| **Healthcare provider level** | 0 | | 1 | | **10** | |
| **Healthcare provider level** | 1 | | 0 | | **10** | |
| **Healthcare provider level** | 0 | | 1 | | **12** | |
| **Healthcare provider level** | 1 | | 0 | | **12** | |
| **Healthcare system level** | 0 | | 1 | | **16** | |
| **Healthcare provider level** | 0 | | 1 | | **16** | |
| **Healthcare provider level** | 0 | | 1 | | **18** | |
| **CPG level** | 1 | | 0 | | **22** | |
| **Healthcare system level** | 1 | | 0 | | **22** | |
| **Healthcare system level** | 0 | | 1 | | **22** | |
| **Healthcare system level** | 1 | | 0 | | **22** | |
| **Healthcare system level** | 1 | | 0 | | **22** | |
| **Healthcare system level** | 0 | | 1 | | **24** | |
| **Healthcare system level** | 1 | | 0 | | **26** | |
| **Healthcare system level** | 0 | | 1 | | **26** | |
| **Healthcare system level** | 1 | | 0 | | **26** | |
| **Healthcare system level** | 1 | | 0 | | **26** | |
| **Healthcare system level** | 1 | | 0 | | **28** | |
| **Healthcare system level** | 0 | | 1 | | **28** | |
| **Healthcare provider level** | 1 | | 0 | | **28** | |
| **Healthcare system level** | 1 | | 0 | | **28** | |
| **Healthcare system level** | 1 | | 1 | | **28** | |
| **Healthcare provider level** | 0 | | 1 | | **28** | |
| **Healthcare system level** | 1 | | 0 | | **28** | |
| **Healthcare system level** | 1 | | 0 | | **30** | |
| **Healthcare system level** | 1 | | 0 | | **30** | |
| **Healthcare provider level** | 1 | | 0 | | **30** | |
| **Healthcare system level** | 1 | | 0 | | **38** | |
| **Healthcare system level** | 1 | | 0 | | **40** | |
| **Healthcare system level** | 1 | | 0 | | **44** | |
| **Healthcare system level** | 1 | | 0 | | **46** | |
| **Healthcare system level** | 1 | | 0 | | **48** | |
| **Healthcare system level** | 1 | | 0 | | **48** | |
| **Healthcare system level** | 1 | | 0 | | **50** | |
| **Healthcare provider level** | 1 | | 0 | | **52** | |
| **Healthcare provider level** | 1 | | 0 | | **54** | |
| **Healthcare system level** | 1 | | 0 | | **54** | |
| **Healthcare system level** | 1 | | 0 | | **56** | |
| **Healthcare provider level** | 1 | | 0 | | **56** | |
| **Healthcare provider level** | 1 | | 0 | | **58** | |
| **Healthcare provider level** | 1 | | 0 | | **60** | |

## Interview 5

| **Overlaps of code segments in sum** | | | | | | |
| --- | --- | --- | --- | --- | --- | --- |
| **Allocated main category** | **Matching**  **(strict)** | **Non-Matching**  **(strict)** | **Matching in % (strict)** | **Matching**  **(less strict)** | **Non-Matching**  **(less strict)** | **Matching in % (less strict)** |
| **Patient level** | 2 | 14 | 13% | 6 | 10 | 38% |
| **Healthcare provider level** | 4 | 18 | 18% | 13 | 9 | 59% |
| **CPG level** | 0 | 2 | 0% | 0 | 2 | 0% |
| **Healthcare system level** | 3 | 4 | 43% | 5 | 2 | 71% |
| **<Total>** | **9** | **38** | **19%** | **24** | **23** | **51%** |
| **Overlaps of code segments per paragraph** | | | | | | |
| **Allocated main category** | **CODER 1** | | **CODER 2** | | **Paragraph** | |
| **Healthcare provider level** | 1 | | 0 | | **2** | |
| **Healthcare provider level** | 1 | | 1 | | **4** | |
| **Patient level** | 1 | | 1 | | **6** | |
| **Healthcare provider level** | 0 | | 1 | | **6** | |
| **Healthcare system level** | 1 | | 0 | | **6** | |
| **Healthcare provider level** | 1 | | 0 | | **6** | |
| **Healthcare system level** | 0 | | 1 | | **6** | |
| **Healthcare provider level** | 1 | | 0 | | **12** | |
| **Healthcare provider level** | 0 | | 1 | | **12** | |
| **Healthcare provider level** | 0 | | 1 | | **12** | |
| **Healthcare provider level** | 0 | | 1 | | **12** | |
| **Healthcare provider level** | 1 | | 0 | | **16** | |
| **Patient level** | 0 | | 1 | | **18** | |
| **Patient level** | 1 | | 0 | | **18** | |
| **Patient level** | 1 | | 0 | | **18** | |
| **Healthcare provider level** | 1 | | 1 | | **18** | |
| **Healthcare provider level** | 1 | | 0 | | **20** | |
| **Healthcare provider level** | 0 | | 1 | | **20** | |
| **Patient level** | 1 | | 0 | | **20** | |
| **Patient level** | 0 | | 1 | | **20** | |
| **Patient level** | 1 | | 0 | | **20** | |
| **Patient level** | 1 | | 0 | | **20** | |
| **Patient level** | 1 | | 0 | | **20** | |
| **Patient level** | 1 | | 0 | | **22** | |
| **Healthcare provider level** | 0 | | 1 | | **22** | |
| **Healthcare provider level** | 1 | | 0 | | **22** | |
| **Patient level** | 1 | | 0 | | **26** | |
| **Patient level** | 0 | | 1 | | **26** | |
| **Healthcare provider level** | 0 | | 1 | | **26** | |
| **Patient level** | 1 | | 0 | | **26** | |
| **Patient level** | 1 | | 0 | | **26** | |
| **Patient level** | 1 | | 0 | | **26** | |
| **Patient level** | 1 | | 1 | | **28** | |
| **Healthcare provider level** | 1 | | 0 | | **30** | |
| **Healthcare provider level** | 0 | | 1 | | **30** | |
| **Healthcare provider level** | 0 | | 1 | | **30** | |
| **Healthcare system level** | 1 | | 1 | | **34** | |
| **Healthcare system level** | 0 | | 1 | | **36** | |
| **Healthcare system level** | 1 | | 1 | | **36** | |
| **CPG level** | 1 | | 0 | | **36** | |
| **Healthcare system level** | 0 | | 1 | | **42** | |
| **Healthcare provider level** | 1 | | 1 | | **44** | |
| **Healthcare provider level** | 1 | | 1 | | **46** | |
| **Healthcare provider level** | 0 | | 1 | | **48** | |
| **Healthcare provider level** | 0 | | 1 | | **52** | |
| **CPG level** | 1 | | 0 | | **52** | |
| **Healthcare system level** | 1 | | 1 | | **54** | |

## Interview 6

| **Overlaps of code segments in sum** | | | | | | |
| --- | --- | --- | --- | --- | --- | --- |
| **Allocated main category** | **Matching**  **(strict)** | **Non-Matching**  **(strict)** | **Matching in % (strict)** | **Matching**  **(less strict)** | **Non-Matching**  **(less strict)** | **Matching in % (less strict)** |
| **Patient level** | 0 | 3 | 0% | 0 | 3 | 0% |
| **Healthcare provider level** | 0 | 20 | 0% | 13 | 7 | 65% |
| **CPG level** | 0 | 1 | 0% | 0 | 1 | 0% |
| **Healthcare system level** | 0 | 12 | 0% | 6 | 6 | 50% |
| **<Total>** | **0** | **36** | **0%** | **19** | **17** | **53%** |
| **Overlaps of code segments per paragraph** | | | | | | |
| **Allocated main category** | **CODER 1** | | **CODER 2** | | **Paragraph** | |
| **Healthcare provider level** | 1 | | 0 | | **2** | |
| **Healthcare provider level** | 0 | | 1 | | **2** | |
| **Healthcare provider level** | 0 | | 1 | | **2** | |
| **Healthcare system level** | 1 | | 0 | | **2** | |
| **Healthcare system level** | 0 | | 1 | | **2** | |
| **Healthcare provider level** | 1 | | 0 | | **4** | |
| **Healthcare provider level** | 0 | | 1 | | **4** | |
| **Healthcare provider level** | 0 | | 1 | | **4** | |
| **Healthcare provider level** | 0 | | 1 | | **6** | |
| **Healthcare system level** | 0 | | 1 | | **6** | |
| **Healthcare provider level** | 1 | | 0 | | **8** | |
| **Healthcare provider level** | 0 | | 1 | | **8** | |
| **Patient level** | 0 | | 1 | | **8** | |
| **Healthcare provider level** | 0 | | 1 | | **8** | |
| **Healthcare provider level** | 0 | | 1 | | **8** | |
| **Healthcare provider level** | 1 | | 0 | | **10** | |
| **Healthcare provider level** | 0 | | 1 | | **10** | |
| **Healthcare provider level** | 1 | | 0 | | **12** | |
| **Healthcare provider level** | 0 | | 1 | | **14** | |
| **Healthcare provider level** | 1 | | 0 | | **14** | |
| **CPG level** | 1 | | 0 | | **14** | |
| **Healthcare provider level** | 1 | | 0 | | **18** | |
| **Healthcare provider level** | 0 | | 1 | | **18** | |
| **Healthcare system level** | 0 | | 1 | | **26** | |
| **Patient level** | 1 | | 0 | | **32** | |
| **Healthcare provider level** | 0 | | 1 | | **34** | |
| **Healthcare system level** | 0 | | 1 | | **34** | |
| **Healthcare system level** | 0 | | 1 | | **34** | |
| **Healthcare provider level** | 1 | | 0 | | **36** | |
| **Healthcare system level** | 0 | | 1 | | **36** | |
| **Healthcare system level** | 0 | | 1 | | **36** | |
| **Healthcare system level** | 1 | | 0 | | **36** | |
| **Healthcare system level** | 1 | | 0 | | **38** | |
| **Healthcare system level** | 0 | | 1 | | **38** | |
| **Healthcare system level** | 0 | | 1 | | **38** | |
| **Patient level** | 1 | | 0 | | **44** | |

## Interview 7

| **Overlaps of code segments in sum** | | | | | | |
| --- | --- | --- | --- | --- | --- | --- |
| **Allocated main category** | **Matching**  **(strict)** | **Non-Matching**  **(strict)** | **Matching in % (strict)** | **Matching**  **(less strict)** | **Non-Matching**  **(less strict)** | **Matching in % (less strict)** |
| **Patient level** | 0 | 1 | 0% | 0 | 1 | 0% |
| **Healthcare provider level** | 7 | 16 | 30% | 15 | 8 | 65% |
| **CPG level** | 0 | 0 | - | 0 | 0 | - |
| **Healthcare system level** | 5 | 14 | 26% | 13 | 6 | 68% |
| **<Total>** | **12** | **31** | **28%** | **28** | **15** | **65%** |
| **Overlaps of code segments per paragraph** | | | | | | |
| **Allocated main category** | **CODER 1** | | **CODER 2** | | **Paragraph** | |
| **Healthcare provider level** | 1 | | 1 | | **2** | |
| **Healthcare provider level** | 1 | | 1 | | **4** | |
| **Healthcare provider level** | 1 | | 1 | | **6** | |
| **Patient level** | 0 | | 1 | | **8** | |
| **Healthcare provider level** | 1 | | 1 | | **8** | |
| **Healthcare provider level** | 1 | | 1 | | **10** | |
| **Healthcare system level** | 1 | | 1 | | **14** | |
| **Healthcare provider level** | 1 | | 1 | | **14** | |
| **Healthcare system level** | 0 | | 1 | | **20** | |
| **Healthcare system level** | 1 | | 1 | | **22** | |
| **Healthcare provider level** | 1 | | 0 | | **22** | |
| **Healthcare provider level** | 1 | | 0 | | **30** | |
| **Healthcare provider level** | 0 | | 1 | | **30** | |
| **Healthcare provider level** | 1 | | 0 | | **30** | |
| **Healthcare provider level** | 1 | | 0 | | **30** | |
| **Healthcare provider level** | 1 | | 0 | | **40** | |
| **Healthcare system level** | 1 | | 0 | | **48** | |
| **Healthcare system level** | 0 | | 1 | | **48** | |
| **Healthcare system level** | 1 | | 0 | | **48** | |
| **Healthcare provider level** | 1 | | 1 | | **50** | |
| **Healthcare provider level** | 1 | | 0 | | **52** | |
| **Healthcare provider level** | 0 | | 1 | | **52** | |
| **Healthcare system level** | 1 | | 1 | | **52** | |
| **Healthcare provider level** | 1 | | 0 | | **56** | |
| **Healthcare system level** | 1 | | 0 | | **56** | |
| **Healthcare system level** | 0 | | 1 | | **56** | |
| **Healthcare system level** | 0 | | 1 | | **56** | |
| **Healthcare provider level** | 1 | | 0 | | **60** | |
| **Healthcare provider level** | 0 | | 1 | | **60** | |
| **Healthcare system level** | 0 | | 1 | | **60** | |
| **Healthcare provider level** | 1 | | 0 | | **60** | |
| **Healthcare system level** | 1 | | 0 | | **60** | |
| **Healthcare provider level** | 1 | | 0 | | **66** | |
| **Healthcare provider level** | 0 | | 1 | | **66** | |
| **Healthcare provider level** | 1 | | 0 | | **70** | |
| **Healthcare system level** | 1 | | 1 | | **70** | |
| **Healthcare system level** | 1 | | 1 | | **72** | |
| **Healthcare system level** | 1 | | 0 | | **74** | |
| **Healthcare system level** | 0 | | 1 | | **74** | |
| **Healthcare system level** | 1 | | 0 | | **76** | |
| **Healthcare provider level** | 0 | | 1 | | **76** | |
| **Healthcare system level** | 0 | | 1 | | **76** | |
| **Healthcare system level** | 1 | | 0 | | **76** | |

## Interview 8

| **Overlaps of code segments in sum** | | | | | | |
| --- | --- | --- | --- | --- | --- | --- |
| **Allocated main category** | **Matching**  **(strict)** | **Non-Matching**  **(strict)** | **Matching in % (strict)** | **Matching**  **(less strict)** | **Non-Matching**  **(less strict)** | **Matching in % (less strict)** |
| **Patient level** | 1 | 4 | 20% | 1 | 4 | 20% |
| **Healthcare provider level** | 4 | 33 | 11% | 25 | 12 | 68% |
| **CPG level** | 1 | 1 | 50% | 1 | 1 | 50% |
| **Healthcare system level** | 3 | 12 | 20% | 10 | 5 | 67% |
| **<Total>** | **9** | **50** | **15%** | **37** | **22** | **63%** |
| **Overlaps of code segments per paragraph** | | | | | | |
| **Allocated main category** | **CODER 1** | | **CODER 2** | | **Paragraph** | |
| **Healthcare provider level** | 1 | | 1 | | **2** | |
| **Healthcare provider level** | 1 | | 0 | | **4** | |
| **Healthcare provider level** | 0 | | 1 | | **4** | |
| **Healthcare provider level** | 1 | | 0 | | **4** | |
| **Healthcare provider level** | 1 | | 0 | | **4** | |
| **Healthcare provider level** | 1 | | 0 | | **6** | |
| **Healthcare provider level** | 0 | | 1 | | **6** | |
| **Healthcare provider level** | 0 | | 1 | | **6** | |
| **Healthcare provider level** | 1 | | 0 | | **8** | |
| **Healthcare provider level** | 0 | | 1 | | **8** | |
| **Healthcare provider level** | 1 | | 0 | | **10** | |
| **Healthcare provider level** | 0 | | 1 | | **10** | |
| **Healthcare provider level** | 1 | | 0 | | **12** | |
| **Healthcare provider level** | 0 | | 1 | | **12** | |
| **Healthcare system level** | 0 | | 1 | | **12** | |
| **Healthcare provider level** | 1 | | 0 | | **12** | |
| **Healthcare provider level** | 1 | | 0 | | **12** | |
| **Healthcare provider level** | 0 | | 1 | | **12** | |
| **Healthcare provider level** | 1 | | 0 | | **12** | |
| **Healthcare provider level** | 1 | | 0 | | **12** | |
| **Healthcare provider level** | 1 | | 0 | | **12** | |
| **Healthcare provider level** | 1 | | 1 | | **12** | |
| **Healthcare system level** | 1 | | 0 | | **14** | |
| **Healthcare provider level** | 1 | | 0 | | **16** | |
| **Healthcare provider level** | 0 | | 1 | | **16** | |
| **Healthcare provider level** | 0 | | 1 | | **16** | |
| **Healthcare provider level** | 0 | | 1 | | **16** | |
| **Healthcare provider level** | 0 | | 1 | | **16** | |
| **Healthcare system level** | 1 | | 0 | | **16** | |
| **Patient level** | 1 | | 1 | | **16** | |
| **Healthcare system level** | 1 | | 1 | | **16** | |
| **Healthcare system level** | 1 | | 1 | | **16** | |
| **Healthcare provider level** | 1 | | 1 | | **16** | |
| **Healthcare system level** | 1 | | 0 | | **16** | |
| **Healthcare system level** | 0 | | 1 | | **16** | |
| **Healthcare system level** | 1 | | 0 | | **16** | |
| **Healthcare system level** | 0 | | 1 | | **16** | |
| **Healthcare provider level** | 0 | | 1 | | **16** | |
| **Healthcare system level** | 1 | | 0 | | **16** | |
| **Healthcare system level** | 0 | | 1 | | **16** | |
| **Healthcare system level** | 0 | | 1 | | **16** | |
| **Healthcare provider level** | 1 | | 0 | | **16** | |
| **Healthcare provider level** | 0 | | 1 | | **16** | |
| **Healthcare provider level** | 1 | | 0 | | **16** | |
| **Healthcare provider level** | 0 | | 1 | | **16** | |
| **Healthcare provider level** | 1 | | 0 | | **18** | |
| **CPG level** | 1 | | 1 | | **18** | |
| **Healthcare system level** | 1 | | 1 | | **18** | |
| **Patient level** | 1 | | 0 | | **18** | |
| **Patient level** | 1 | | 0 | | **18** | |
| **Patient level** | 1 | | 0 | | **18** | |
| **Patient level** | 1 | | 0 | | **20** | |
| **CPG level** | 1 | | 0 | | **20** | |
| **Healthcare provider level** | 1 | | 1 | | **22** | |
| **Healthcare provider level** | 0 | | 1 | | **30** | |
| **Healthcare provider level** | 1 | | 0 | | **30** | |
| **Healthcare system level** | 1 | | 0 | | **30** | |
| **Healthcare system level** | 1 | | 0 | | **32** | |
| **Healthcare provider level** | 1 | | 0 | | **38** | |

## Interview 9

| **Overlaps of code segments in sum** | | | | | | |
| --- | --- | --- | --- | --- | --- | --- |
| **Allocated main category** | **Matching**  **(strict)** | **Non-Matching**  **(strict)** | **Matching in % (strict)** | **Matching**  **(less strict)** | **Non-Matching**  **(less strict)** | **Matching in % (less strict)** |
| **Patient level** | 0 | 1 | 0% | 0 | 1 | 0% |
| **Healthcare provider level** | 9 | 38 | 19% | 33 | 14 | 70% |
| **CPG level** | 0 | 7 | 0% | 6 | 1 | 86% |
| **Healthcare system level** | 1 | 11 | 8% | 3 | 9 | 25% |
| **<Total>** | **10** | **57** | **15%** | **42** | **25** | **63%** |
| **Overlaps of code segments per paragraph** | | | | | | |
| **Allocated main category** | **CODER 1** | | **CODER 2** | | **Paragraph** | |
| **Healthcare provider level** | 1 | | 1 | | **2** | |
| **Healthcare provider level** | 1 | | 1 | | **4** | |
| **Healthcare provider level** | 0 | | 1 | | **6** | |
| **Healthcare provider level** | 0 | | 1 | | **6** | |
| **Healthcare provider level** | 1 | | 0 | | **8** | |
| **Healthcare provider level** | 0 | | 1 | | **8** | |
| **Healthcare provider level** | 1 | | 0 | | **8** | |
| **Healthcare provider level** | 1 | | 0 | | **8** | |
| **Healthcare provider level** | 1 | | 1 | | **8** | |
| **Healthcare provider level** | 1 | | 1 | | **10** | |
| **Healthcare provider level** | 1 | | 1 | | **10** | |
| **Healthcare provider level** | 1 | | 0 | | **10** | |
| **Healthcare provider level** | 0 | | 1 | | **10** | |
| **Healthcare provider level** | 1 | | 0 | | **12** | |
| **CPG level** | 1 | | 0 | | **12** | |
| **CPG level** | 0 | | 1 | | **12** | |
| **Healthcare provider level** | 1 | | 1 | | **12** | |
| **Healthcare provider level** | 1 | | 0 | | **12** | |
| **Healthcare provider level** | 1 | | 0 | | **12** | |
| **Healthcare provider level** | 0 | | 1 | | **12** | |
| **Healthcare system level** | 1 | | 0 | | **12** | |
| **Healthcare system level** | 0 | | 1 | | **12** | |
| **Healthcare system level** | 1 | | 0 | | **12** | |
| **CPG level** | 1 | | 0 | | **12** | |
| **Healthcare system level** | 1 | | 0 | | **12** | |
| **CPG level** | 0 | | 1 | | **12** | |
| **Healthcare system level** | 0 | | 1 | | **12** | |
| **Healthcare provider level** | 1 | | 0 | | **14** | |
| **Healthcare system level** | 1 | | 0 | | **14** | |
| **Healthcare system level** | 0 | | 1 | | **14** | |
| **Healthcare provider level** | 1 | | 1 | | **14** | |
| **Healthcare system level** | 1 | | 0 | | **14** | |
| **Healthcare provider level** | 1 | | 0 | | **14** | |
| **Healthcare provider level** | 0 | | 1 | | **14** | |
| **Healthcare provider level** | 1 | | 0 | | **14** | |
| **Healthcare system level** | 1 | | 0 | | **14** | |
| **Healthcare provider level** | 1 | | 0 | | **18** | |
| **Healthcare provider level** | 0 | | 1 | | **18** | |
| **Healthcare provider level** | 1 | | 0 | | **18** | |
| **Healthcare provider level** | 0 | | 1 | | **18** | |
| **Healthcare system level** | 1 | | 0 | | **18** | |
| **Healthcare system level** | 0 | | 1 | | **18** | |
| **Healthcare provider level** | 1 | | 0 | | **18** | |
| **Healthcare provider level** | 0 | | 1 | | **18** | |
| **Healthcare provider level** | 1 | | 0 | | **20** | |
| **Healthcare provider level** | 0 | | 1 | | **20** | |
| **Healthcare provider level** | 1 | | 0 | | **20** | |
| **Healthcare provider level** | 1 | | 0 | | **20** | |
| **Healthcare provider level** | 1 | | 0 | | **20** | |
| **Healthcare provider level** | 0 | | 1 | | **20** | |
| **Healthcare provider level** | 1 | | 1 | | **20** | |
| **CPG level** | 1 | | 0 | | **20** | |
| **Healthcare provider level** | 1 | | 0 | | **22** | |
| **Healthcare provider level** | 0 | | 1 | | **22** | |
| **Healthcare system level** | 1 | | 1 | | **22** | |
| **Healthcare provider level** | 1 | | 0 | | **22** | |
| **Healthcare provider level** | 1 | | 0 | | **22** | |
| **Patient level** | 1 | | 0 | | **26** | |
| **Healthcare provider level** | 1 | | 0 | | **26** | |
| **Healthcare provider level** | 0 | | 1 | | **26** | |
| **Healthcare provider level** | 1 | | 0 | | **30** | |
| **Healthcare provider level** | 0 | | 1 | | **30** | |
| **Healthcare provider level** | 1 | | 0 | | **34** | |
| **Healthcare provider level** | 0 | | 1 | | **34** | |
| **Healthcare provider level** | 1 | | 1 | | **40** | |
| **CPG level** | 1 | | 0 | | **40** | |
| **CPG level** | 0 | | 1 | | **40** | |

## Interview 10

| **Overlaps of code segments in sum** | | | | | | |
| --- | --- | --- | --- | --- | --- | --- |
| **Allocated main category** | **Matching**  **(strict)** | **Non-Matching**  **(strict)** | **Matching in % (strict)** | **Matching**  **(less strict)** | **Non-Matching**  **(less strict)** | **Matching in % (less strict)** |
| **Patient level** | 1 | 9 | 10% | 1 | 9 | 10% |
| **Healthcare provider level** | 2 | 34 | 6% | 17 | 19 | 47% |
| **CPG level** | 0 | 0 | - | 0 | 0 | - |
| **Healthcare system level** | 1 | 9 | 10% | 1 | 9 | 10% |
| **<Total>** | **4** | **52** | **7%** | **19** | **37** | **34%** |
| **Overlaps of code segments per paragraph** | | | | | | |
| **Allocated main category** | **CODER 1** | | **CODER 2** | | **Paragraph** | |
| **Healthcare provider level** | 0 | | 1 | | **2** | |
| **Healthcare provider level** | 1 | | 0 | | **2** | |
| **Healthcare provider level** | 1 | | 0 | | **2** | |
| **Healthcare system level** | 1 | | 0 | | **2** | |
| **Healthcare system level** | 0 | | 1 | | **2** | |
| **Patient level** | 0 | | 1 | | **4** | |
| **Healthcare provider level** | 1 | | 0 | | **4** | |
| **Healthcare provider level** | 1 | | 1 | | **6** | |
| **Healthcare provider level** | 1 | | 0 | | **8** | |
| **Healthcare provider level** | 0 | | 1 | | **8** | |
| **Healthcare provider level** | 1 | | 1 | | **12** | |
| **Healthcare provider level** | 1 | | 0 | | **14** | |
| **Healthcare provider level** | 1 | | 0 | | **14** | |
| **Healthcare system level** | 0 | | 1 | | **16** | |
| **Healthcare system level** | 1 | | 0 | | **16** | |
| **Healthcare provider level** | 1 | | 0 | | **20** | |
| **Healthcare provider level** | 0 | | 1 | | **20** | |
| **Healthcare provider level** | 0 | | 1 | | **20** | |
| **Healthcare provider level** | 0 | | 1 | | **20** | |
| **Healthcare provider level** | 1 | | 0 | | **24** | |
| **Healthcare provider level** | 0 | | 1 | | **24** | |
| **Healthcare system level** | 0 | | 1 | | **24** | |
| **Healthcare provider level** | 1 | | 0 | | **24** | |
| **Patient level** | 1 | | 0 | | **28** | |
| **Healthcare provider level** | 1 | | 0 | | **28** | |
| **Healthcare provider level** | 1 | | 0 | | **28** | |
| **Healthcare provider level** | 0 | | 1 | | **28** | |
| **Healthcare system level** | 1 | | 1 | | **28** | |
| **Healthcare provider level** | 1 | | 0 | | **28** | |
| **Healthcare system level** | 0 | | 1 | | **28** | |
| **Healthcare provider level** | 0 | | 1 | | **28** | |
| **Patient level** | 1 | | 1 | | **30** | |
| **Healthcare provider level** | 1 | | 0 | | **32** | |
| **Healthcare system level** | 1 | | 0 | | **32** | |
| **Healthcare provider level** | 0 | | 1 | | **32** | |
| **Patient level** | 1 | | 0 | | **36** | |
| **Healthcare system level** | 0 | | 1 | | **38** | |
| **Healthcare provider level** | 1 | | 0 | | **44** | |
| **Healthcare provider level** | 0 | | 1 | | **44** | |
| **Healthcare provider level** | 0 | | 1 | | **46** | |
| **Healthcare provider level** | 1 | | 0 | | **46** | |
| **Patient level** | 1 | | 0 | | **48** | |
| **Healthcare provider level** | 1 | | 0 | | **48** | |
| **Healthcare provider level** | 0 | | 1 | | **48** | |
| **Patient level** | 0 | | 1 | | **48** | |
| **Healthcare provider level** | 1 | | 0 | | **50** | |
| **Healthcare provider level** | 0 | | 1 | | **50** | |
| **Healthcare provider level** | 0 | | 1 | | **50** | |
| **Healthcare provider level** | 1 | | 0 | | **52** | |
| **Healthcare provider level** | 0 | | 1 | | **52** | |
| **Patient level** | 1 | | 0 | | **52** | |
| **Healthcare provider level** | 1 | | 0 | | **54** | |
| **Patient level** | 0 | | 1 | | **56** | |
| **Patient level** | 0 | | 1 | | **58** | |
| **Healthcare system level** | 0 | | 1 | | **60** | |
| **Patient level** | 0 | | 1 | | **62** | |

## Interview 11

| **Overlaps of code segments in sum** | | | | | | |
| --- | --- | --- | --- | --- | --- | --- |
| **Allocated main category** | **Matching**  **(strict)** | **Non-Matching**  **(strict)** | **Matching in % (strict)** | **Matching**  **(less strict)** | **Non-Matching**  **(less strict)** | **Matching in % (less strict)** |
| **Patient level** | 0 | 6 | 0% | 2 | 4 | 33% |
| **Healthcare provider level** | 6 | 28 | 18% | 18 | 16 | 53% |
| **CPG level** | 0 | 3 | 0% | 0 | 3 | 0% |
| **Healthcare system level** | 1 | 7 | 13% | 3 | 5 | 38% |
| **<Total>** | **7** | **44** | **14%** | **23** | **28** | **45%** |
| **Overlaps of code segments per paragraph** | | | | | | |
| **Allocated main category** | **CODER 1** | | **CODER 2** | | **Paragraph** | |
| **Healthcare provider level** | 1 | | 0 | | **2** | |
| **Healthcare provider level** | 0 | | 1 | | **2** | |
| **Healthcare provider level** | 0 | | 1 | | **2** | |
| **Healthcare provider level** | 0 | | 1 | | **2** | |
| **Healthcare provider level** | 1 | | 1 | | **4** | |
| **Healthcare provider level** | 1 | | 1 | | **4** | |
| **Patient level** | 0 | | 1 | | **4** | |
| **Healthcare provider level** | 1 | | 0 | | **4** | |
| **Healthcare provider level** | 0 | | 1 | | **4** | |
| **Healthcare system level** | 1 | | 0 | | **4** | |
| **Healthcare system level** | 0 | | 1 | | **4** | |
| **Healthcare provider level** | 0 | | 1 | | **4** | |
| **Patient level** | 1 | | 0 | | **6** | |
| **Healthcare provider level** | 1 | | 0 | | **6** | |
| **Healthcare provider level** | 0 | | 1 | | **6** | |
| **CPG level** | 1 | | 0 | | **6** | |
| **Healthcare provider level** | 0 | | 1 | | **6** | |
| **Healthcare provider level** | 1 | | 0 | | **8** | |
| **CPG level** | 0 | | 1 | | **8** | |
| **Healthcare provider level** | 0 | | 1 | | **8** | |
| **Patient level** | 1 | | 0 | | **8** | |
| **Healthcare provider level** | 1 | | 0 | | **8** | |
| **Patient level** | 0 | | 1 | | **8** | |
| **Healthcare provider level** | 0 | | 1 | | **8** | |
| **Healthcare provider level** | 0 | | 1 | | **8** | |
| **Healthcare provider level** | 1 | | 0 | | **8** | |
| **Healthcare provider level** | 1 | | 1 | | **10** | |
| **CPG level** | 1 | | 0 | | **10** | |
| **Healthcare provider level** | 1 | | 1 | | **10** | |
| **Healthcare provider level** | 0 | | 1 | | **12** | |
| **Healthcare provider level** | 1 | | 0 | | **12** | |
| **Healthcare provider level** | 1 | | 0 | | **12** | |
| **Healthcare system level** | 0 | | 1 | | **12** | |
| **Healthcare provider level** | 0 | | 1 | | **12** | |
| **Healthcare system level** | 1 | | 0 | | **12** | |
| **Healthcare provider level** | 1 | | 0 | | **14** | |
| **Healthcare provider level** | 0 | | 1 | | **14** | |
| **Healthcare provider level** | 0 | | 1 | | **14** | |
| **Healthcare provider level** | 1 | | 1 | | **14** | |
| **Healthcare system level** | 1 | | 1 | | **14** | |
| **Healthcare provider level** | 0 | | 1 | | **24** | |
| **Healthcare system level** | 0 | | 1 | | **26** | |
| **Healthcare system level** | 0 | | 1 | | **28** | |
| **Healthcare provider level** | 0 | | 1 | | **32** | |
| **Healthcare provider level** | 1 | | 0 | | **32** | |
| **Healthcare provider level** | 0 | | 1 | | **32** | |
| **Healthcare provider level** | 1 | | 0 | | **34** | |
| **Patient level** | 0 | | 1 | | **34** | |
| **Patient level** | 1 | | 0 | | **34** | |
| **Healthcare provider level** | 1 | | 1 | | **36** | |
| **Healthcare system level** | 1 | | 0 | | **36** | |

## Interview 12

| **Overlaps of code segments in sum** | | | | | | |
| --- | --- | --- | --- | --- | --- | --- |
| **Allocated main category** | **Matching**  **(strict)** | **Non-Matching**  **(strict)** | **Matching in % (strict)** | **Matching**  **(less strict)** | **Non-Matching**  **(less strict)** | **Matching in % (less strict)** |
| **Patient level** | 3 | 4 | 43% | 5 | 2 | 71% |
| **Healthcare provider level** | 12 | 21 | 36% | 18 | 15 | 55% |
| **CPG level** | 1 | 2 | 33% | 1 | 2 | 33% |
| **Healthcare system level** | 4 | 22 | 15% | 8 | 18 | 31% |
| **<Total>** | **20** | **49** | **29%** | **32** | **37** | **46%** |
| **Overlaps of code segments per paragraph** | | | | | | |
| **Allocated main category** | **CODER 1** | | **CODER 2** | | **Paragraph** | |
| **Healthcare provider level** | 1 | | 1 | | **4** | |
| **Healthcare provider level** | 1 | | 1 | | **4** | |
| **Healthcare provider level** | 1 | | 1 | | **4** | |
| **Healthcare system level** | 1 | | 0 | | **4** | |
| **Healthcare system level** | 0 | | 1 | | **4** | |
| **Healthcare provider level** | 1 | | 1 | | **4** | |
| **Healthcare system level** | 1 | | 0 | | **4** | |
| **Patient level** | 0 | | 1 | | **4** | |
| **Patient level** | 1 | | 0 | | **4** | |
| **Healthcare system level** | 0 | | 1 | | **4** | |
| **Healthcare system level** | 1 | | 0 | | **4** | |
| **Healthcare provider level** | 1 | | 1 | | **8** | |
| **Healthcare provider level** | 1 | | 1 | | **10** | |
| **CPG level** | 1 | | 0 | | **10** | |
| **Healthcare system level** | 1 | | 0 | | **10** | |
| **Healthcare provider level** | 1 | | 0 | | **10** | |
| **Healthcare provider level** | 0 | | 1 | | **10** | |
| **CPG level** | 1 | | 0 | | **10** | |
| **Healthcare provider level** | 0 | | 1 | | **10** | |
| **Healthcare provider level** | 1 | | 1 | | **12** | |
| **Healthcare provider level** | 1 | | 0 | | **12** | |
| **Healthcare system level** | 0 | | 1 | | **12** | |
| **Healthcare provider level** | 1 | | 1 | | **14** | |
| **Healthcare provider level** | 1 | | 0 | | **20** | |
| **Healthcare provider level** | 0 | | 1 | | **20** | |
| **Healthcare provider level** | 1 | | 0 | | **22** | |
| **Healthcare provider level** | 0 | | 1 | | **22** | |
| **Healthcare system level** | 1 | | 1 | | **22** | |
| **Healthcare system level** | 1 | | 0 | | **26** | |
| **Healthcare provider level** | 1 | | 0 | | **28** | |
| **Healthcare system level** | 1 | | 0 | | **30** | |
| **Healthcare provider level** | 1 | | 0 | | **32** | |
| **Healthcare system level** | 1 | | 1 | | **32** | |
| **Healthcare system level** | 1 | | 0 | | **36** | |
| **Healthcare system level** | 1 | | 0 | | **38** | |
| **Healthcare system level** | 0 | | 1 | | **38** | |
| **Healthcare provider level** | 1 | | 0 | | **38** | |
| **Healthcare system level** | 0 | | 1 | | **40** | |
| **Healthcare system level** | 1 | | 0 | | **40** | |
| **Healthcare provider level** | 0 | | 1 | | **40** | |
| **Healthcare system level** | 1 | | 0 | | **40** | |
| **Healthcare system level** | 1 | | 0 | | **40** | |
| **Healthcare provider level** | 1 | | 0 | | **40** | |
| **Healthcare system level** | 1 | | 0 | | **40** | |
| **Healthcare provider level** | 1 | | 1 | | **46** | |
| **Healthcare provider level** | 1 | | 0 | | **48** | |
| **Healthcare provider level** | 1 | | 1 | | **48** | |
| **Patient level** | 1 | | 1 | | **50** | |
| **Healthcare provider level** | 0 | | 1 | | **50** | |
| **Patient level** | 1 | | 1 | | **52** | |
| **Healthcare provider level** | 1 | | 1 | | **52** | |
| **Patient level** | 1 | | 0 | | **54** | |
| **Patient level** | 0 | | 1 | | **54** | |
| **CPG level** | 1 | | 1 | | **58** | |
| **Patient level** | 1 | | 1 | | **58** | |
| **Healthcare provider level** | 1 | | 1 | | **58** | |
| **Healthcare system level** | 1 | | 1 | | **58** | |
| **Healthcare system level** | 1 | | 1 | | **64** | |
| **Healthcare provider level** | 1 | | 0 | | **66** | |
| **Healthcare provider level** | 0 | | 1 | | **66** | |
| **Healthcare system level** | 0 | | 1 | | **66** | |
| **Healthcare system level** | 1 | | 0 | | **66** | |
| **Healthcare provider level** | 1 | | 0 | | **66** | |
| **Healthcare provider level** | 1 | | 0 | | **68** | |
| **Healthcare system level** | 0 | | 1 | | **68** | |
| **Healthcare provider level** | 1 | | 0 | | **70** | |
| **Healthcare provider level** | 0 | | 1 | | **70** | |
| **Healthcare system level** | 0 | | 1 | | **70** | |
| **Healthcare system level** | 0 | | 1 | | **70** | |

## Interview 13

| **Overlaps of code segments in sum** | | | | | | |
| --- | --- | --- | --- | --- | --- | --- |
| **Allocated main category** | **Matching**  **(strict)** | **Non-Matching**  **(strict)** | **Matching in % (strict)** | **Matching**  **(less strict)** | **Non-Matching**  **(less strict)** | **Matching in % (less strict)** |
| **Patient level** | 0 | 9 | 0% | 4 | 5 | 44% |
| **Healthcare provider level** | 3 | 26 | 10% | 10 | 19 | 34% |
| **CPG level** | 0 | 0 | - | 0 | 0 | - |
| **Healthcare system level** | 2 | 28 | 7% | 10 | 20 | 33% |
| **<Total>** | **5** | **63** | **7%** | **24** | **44** | **35%** |
| **Overlaps of code segments per paragraph** | | | | | | |
| **Allocated main category** | **CODER 1** | | **CODER 2** | | **Paragraph** | |
| **Healthcare provider level** | 1 | | 0 | | **2** | |
| **Healthcare provider level** | 1 | | 1 | | **6** | |
| **Healthcare provider level** | 1 | | 1 | | **6** | |
| **Healthcare provider level** | 1 | | 1 | | **6** | |
| **Patient level** | 1 | | 0 | | **6** | |
| **Healthcare provider level** | 1 | | 0 | | **6** | |
| **Healthcare provider level** | 0 | | 1 | | **6** | |
| **Healthcare system level** | 1 | | 0 | | **6** | |
| **Patient level** | 0 | | 1 | | **6** | |
| **Healthcare provider level** | 0 | | 1 | | **6** | |
| **Healthcare provider level** | 1 | | 0 | | **6** | |
| **Healthcare provider level** | 0 | | 1 | | **8** | |
| **Healthcare provider level** | 1 | | 0 | | **8** | |
| **Patient level** | 0 | | 1 | | **8** | |
| **Healthcare provider level** | 0 | | 1 | | **8** | |
| **Healthcare system level** | 1 | | 0 | | **8** | |
| **Healthcare system level** | 0 | | 1 | | **8** | |
| **Patient level** | 1 | | 0 | | **8** | |
| **Healthcare system level** | 1 | | 1 | | **8** | |
| **Healthcare provider level** | 1 | | 0 | | **8** | |
| **Patient level** | 0 | | 1 | | **10** | |
| **Patient level** | 1 | | 0 | | **10** | |
| **Patient level** | 0 | | 1 | | **10** | |
| **Healthcare system level** | 1 | | 0 | | **10** | |
| **Healthcare system level** | 0 | | 1 | | **10** | |
| **Patient level** | 1 | | 0 | | **12** | |
| **Healthcare provider level** | 1 | | 0 | | **12** | |
| **Healthcare provider level** | 0 | | 1 | | **12** | |
| **Healthcare system level** | 1 | | 0 | | **12** | |
| **Healthcare provider level** | 1 | | 0 | | **12** | |
| **Healthcare provider level** | 0 | | 1 | | **12** | |
| **Healthcare system level** | 1 | | 0 | | **12** | |
| **Healthcare system level** | 1 | | 0 | | **14** | |
| **Healthcare system level** | 0 | | 1 | | **14** | |
| **Healthcare provider level** | 0 | | 1 | | **14** | |
| **Healthcare system level** | 0 | | 1 | | **14** | |
| **Healthcare system level** | 1 | | 0 | | **14** | |
| **Healthcare system level** | 1 | | 0 | | **14** | |
| **Healthcare system level** | 1 | | 0 | | **14** | |
| **Patient level** | 1 | | 0 | | **14** | |
| **Healthcare system level** | 1 | | 0 | | **14** | |
| **Healthcare system level** | 0 | | 1 | | **14** | |
| **Healthcare system level** | 1 | | 0 | | **14** | |
| **Healthcare system level** | 1 | | 0 | | **16** | |
| **Healthcare system level** | 0 | | 1 | | **16** | |
| **Healthcare provider level** | 1 | | 0 | | **16** | |
| **Healthcare system level** | 1 | | 0 | | **16** | |
| **Healthcare provider level** | 1 | | 0 | | **16** | |
| **Healthcare provider level** | 0 | | 1 | | **16** | |
| **Healthcare system level** | 1 | | 0 | | **16** | |
| **Healthcare provider level** | 0 | | 1 | | **18** | |
| **Healthcare provider level** | 1 | | 0 | | **18** | |
| **Healthcare provider level** | 1 | | 0 | | **18** | |
| **Healthcare provider level** | 0 | | 1 | | **18** | |
| **Healthcare system level** | 1 | | 0 | | **18** | |
| **Healthcare system level** | 0 | | 1 | | **18** | |
| **Healthcare provider level** | 1 | | 0 | | **18** | |
| **Healthcare provider level** | 1 | | 0 | | **18** | |
| **Healthcare system level** | 1 | | 0 | | **18** | |
| **Healthcare provider level** | 0 | | 1 | | **20** | |
| **Healthcare system level** | 0 | | 1 | | **20** | |
| **Healthcare system level** | 1 | | 1 | | **20** | |
| **Healthcare provider level** | 1 | | 0 | | **26** | |
| **Healthcare provider level** | 1 | | 0 | | **28** | |
| **Healthcare system level** | 0 | | 1 | | **36** | |
| **Healthcare system level** | 1 | | 0 | | **36** | |
| **Healthcare system level** | 1 | | 0 | | **36** | |
| **Healthcare system level** | 1 | | 0 | | **38** | |

## Interview 14

| **Overlaps of code segments in sum** | | | | | | |
| --- | --- | --- | --- | --- | --- | --- |
| **Allocated main category** | **Matching**  **(strict)** | **Non-Matching**  **(strict)** | **Matching in % (strict)** | **Matching**  **(less strict)** | **Non-Matching**  **(less strict)** | **Matching in % (less strict)** |
| **Patient level** | 0 | 1 | 0% | 0 | 1 | 0% |
| **Healthcare provider level** | 3 | 26 | 10% | 17 | 12 | 59% |
| **CPG level** | 0 | 1 | 0% | 0 | 1 | 0% |
| **Healthcare system level** | 2 | 13 | 13% | 4 | 11 | 27% |
| **<Total>** | **5** | **41** | **11%** | **21** | **25** | **46%** |
| **Overlaps of code segments per paragraph** | | | | | | |
| **Allocated main category** | **CODER 1** | | **CODER 2** | | **Paragraph** | |
| **Healthcare provider level** | 1 | | 0 | | **2** | |
| **Healthcare provider level** | 0 | | 1 | | **2** | |
| **Healthcare provider level** | 1 | | 0 | | **2** | |
| **Healthcare provider level** | 1 | | 0 | | **2** | |
| **Healthcare provider level** | 1 | | 0 | | **4** | |
| **Healthcare provider level** | 0 | | 1 | | **4** | |
| **Healthcare provider level** | 1 | | 0 | | **4** | |
| **Patient level** | 0 | | 1 | | **4** | |
| **Healthcare provider level** | 0 | | 1 | | **4** | |
| **Healthcare provider level** | 1 | | 0 | | **4** | |
| **Healthcare provider level** | 1 | | 0 | | **4** | |
| **Healthcare provider level** | 1 | | 0 | | **4** | |
| **Healthcare provider level** | 0 | | 1 | | **6** | |
| **Healthcare provider level** | 1 | | 0 | | **6** | |
| **Healthcare provider level** | 0 | | 1 | | **6** | |
| **Healthcare provider level** | 0 | | 1 | | **6** | |
| **Healthcare provider level** | 0 | | 1 | | **8** | |
| **Healthcare provider level** | 1 | | 0 | | **10** | |
| **Healthcare provider level** | 0 | | 1 | | **10** | |
| **Healthcare system level** | 0 | | 1 | | **10** | |
| **Healthcare system level** | 1 | | 1 | | **10** | |
| **Healthcare system level** | 1 | | 1 | | **10** | |
| **Healthcare provider level** | 0 | | 1 | | **10** | |
| **Healthcare provider level** | 1 | | 0 | | **5** | |
| **Healthcare provider level** | 0 | | 1 | | **10** | |
| **Healthcare provider level** | 1 | | 0 | | **10** | |
| **Healthcare system level** | 0 | | 1 | | **10** | |
| **Healthcare system level** | 1 | | 0 | | **10** | |
| **Healthcare provider level** | 1 | | 1 | | **12** | |
| **Healthcare provider level** | 0 | | 1 | | **12** | |
| **Healthcare system level** | 1 | | 0 | | **12** | |
| **Healthcare provider level** | 1 | | 1 | | **12** | |
| **Healthcare system level** | 1 | | 0 | | **16** | |
| **Healthcare system level** | 1 | | 0 | | **16** | |
| **Healthcare system level** | 0 | | 1 | | **16** | |
| **Healthcare system level** | 0 | | 1 | | **18** | |
| **Healthcare system level** | 0 | | 1 | | **18** | |
| **Healthcare system level** | 0 | | 1 | | **18** | |
| **CPG level** | 1 | | 0 | | **20** | |
| **Healthcare provider level** | 1 | | 1 | | **20** | |
| **Healthcare provider level** | 0 | | 1 | | **20** | |
| **Healthcare system level** | 1 | | 0 | | **20** | |
| **Healthcare system level** | 1 | | 0 | | **20** | |
| **Healthcare provider level** | 1 | | 0 | | **24** | |
| **Healthcare system level** | 0 | | 1 | | **24** | |
| **Healthcare provider level** | 1 | | 0 | | **32** | |

## Interview 15

| **Overlaps of code segments in sum** | | | | | | |
| --- | --- | --- | --- | --- | --- | --- |
| **Allocated main category** | **Matching**  **(strict)** | **Non-Matching**  **(strict)** | **Matching in % (strict)** | **Matching**  **(less strict)** | **Non-Matching**  **(less strict)** | **Matching in % (less strict)** |
| **Patient level** | 0 | 2 | 0% | 2 | 0 | 100% |
| **Healthcare provider level** | 4 | 9 | 31% | 10 | 3 | 77% |
| **CPG level** | 0 | 0 | - | 0 | 0 | - |
| **Healthcare system level** | 1 | 15 | 6% | 5 | 11 | 31% |
| **<Total>** | **5** | **26** | **16%** | **17** | **14** | **55%** |
| **Overlaps of code segments per paragraph** | | | | | | |
| **Allocated main category** | **CODER 1** | | **CODER 2** | | **Paragraph** | |
| **Healthcare provider level** | 1 | | 1 | | **2** | |
| **Patient level** | 0 | | 1 | | **2** | |
| **Patient level** | 1 | | 0 | | **2** | |
| **Healthcare provider level** | 1 | | 1 | | **4** | |
| **Healthcare provider level** | 1 | | 1 | | **8** | |
| **Healthcare provider level** | 1 | | 1 | | **12** | |
| **Healthcare provider level** | 1 | | 0 | | **12** | |
| **Healthcare provider level** | 0 | | 1 | | **12** | |
| **Healthcare provider level** | 1 | | 0 | | **12** | |
| **Healthcare provider level** | 0 | | 1 | | **12** | |
| **Healthcare system level** | 1 | | 0 | | **14** | |
| **Healthcare system level** | 0 | | 1 | | **14** | |
| **Healthcare provider level** | 0 | | 1 | | **14** | |
| **Healthcare system level** | 1 | | 0 | | **14** | |
| **Healthcare system level** | 0 | | 1 | | **14** | |
| **Healthcare system level** | 1 | | 0 | | **14** | |
| **Healthcare system level** | 0 | | 1 | | **14** | |
| **Healthcare system level** | 1 | | 0 | | **14** | |
| **Healthcare system level** | 1 | | 0 | | **16** | |
| **Healthcare system level** | 1 | | 0 | | **16** | |
| **Healthcare system level** | 0 | | 1 | | **20** | |
| **Healthcare system level** | 1 | | 0 | | **20** | |
| **Healthcare system level** | 0 | | 1 | | **24** | |
| **Healthcare provider level** | 0 | | 1 | | **26** | |
| **Healthcare system level** | 1 | | 0 | | **26** | |
| **Healthcare provider level** | 1 | | 0 | | **28** | |
| **Healthcare provider level** | 0 | | 1 | | **28** | |
| **Healthcare provider level** | 1 | | 0 | | **32** | |
| **Healthcare system level** | 0 | | 1 | | **32** | |
| **Healthcare system level** | 1 | | 0 | | **32** | |
| **Healthcare system level** | 1 | | 1 | | **32** | |
