# Supplementary material for: Factors influencing adherence to clinical practice guidelines in patients with suspected chronic coronary syndrome: a qualitative interview study in the ambulatory care sector in Germany
Source: BMC Health Serv Res. 2023 Jun 20;23:655. doi: 10.1186/s12913-023-09587-1 (PMC10283181; doi:10.1186/s12913-023-09587-1)
Supplement: Supplementary file 4 — Supplementary Material 4 [file 12913_2023_9587_MOESM4_ESM.docx]

Coding rules

| **Rule 1: Allocation of sub-categories** | Sub-categories of potential influencing factors will be **allocated to (not coded with!) the following four main categories**:   1. **Level 1: Patients** as in persons with suspected CCS. 2. **Level 2: Healthcare providers** as in professionals who provide healthcare services for persons with suspected CCS. 3. **Level 3: CPGs** as in systematically developed decision-making aids for healthcare providers. 4. **Level 4: Healthcare system** as in the institution that maintains, promotes, and restores the health of the population as well as prevents diseases. |
| --- | --- |
| **Rule 2: Creation of work-around codes** | For the comparison of sub-categories that are initially created independent by different coders (see Additional file 3 “Inter-coder agreement”) work-around coding is mandatory. This means that during the independent coding process, each coder will not only create sub-category suggestion. They will also **use segment-identical work-around codes that indicate to which main category the specific sub-category suggestions should be allocated**.  In order to determine code variables values for each code segment individually (see Rule 5), work-around coding is needed, too. This means that **each code segment that is finally coded with a certain sub-category needs a segment-identical dummy-code**. Otherwise, determining code variables values is only possible at the level of sub-categories, not at the level of code segments. |
| **Rule 3: Consecutive creation sub-categories** | During the consolidation of sub-categories it became clear that certain aspects trigger more than one sub-category due to similar reference points. Hence, a consecutive logic regarding the coding of specific sub-categories was established. Namely, **if a certain code segment of a sub-category is commented with a certain term it triggers coding of another sub-category**:   - Economic structures {32} with “disincentives” or “missing incentives” as trigger terms 🡪 Profitability {20}. - Effort (procedural) {30} with “cost expenditure” as trigger term 🡪 Profitability {20}. - Workload (administration) {31} with “transmission” as trigger term 🡪 Interprofessional healthcare {12}. - Inexpedience {23} with “agreement” as trigger term 🡪 Interprofessional healthcare {12}. - Local structures {33} with “cooperation” as trigger term 🡪 Interprofessional healthcare {12}. - Stipulated structures {34} with “agreement” as trigger term 🡪 Interprofessional healthcare {12}. - Temporal structures {35} with “cooperation” as trigger term 🡪 Interprofessional healthcare {12}. - Temporal structures {35} with “disincentives” or “missing incentives” as trigger terms 🡪 Case-related time pressure {7}. |
| **Rule 4: Usage of signal words** | To identify sub-categories of influencing factors, the following **German signal words can be used to search** for relevant code segments:   - *Herausforderung*/*herausfordernd* - *Hilfe/*Hilfs*/*hilft/*helf* - *Leitlinie*/*leitlinien* - *Lösung*/*lös* - *Optimierung*/*optimier* - *Problem*/*problematisch* - *Schwierigkeit*/*schwierig* - *Verbesserung*/*verbesser* |
| **Rule 5: Determination of code variable values** | The value of the designed four code variables must be determined **for each code segment individually** (see Table 1 “Code variables”). |
