# Supplementary material for: Factors influencing adherence to clinical practice guidelines in patients with suspected chronic coronary syndrome: a qualitative interview study in the ambulatory care sector in Germany
Source: BMC Health Serv Res. 2023 Jun 20;23:655. doi: 10.1186/s12913-023-09587-1 (PMC10283181; doi:10.1186/s12913-023-09587-1)
Supplement: Supplementary file 5 — Supplementary Material 5 [file 12913_2023_9587_MOESM5_ESM.docx]

**Coding relations**

The following tables display coding relations that are calculated based on the distance of code segments. Table 1 provides an overview, that compares (un)weighted relative frequencies of coding relations between different influencing factors at the same level (“inter-level view”) and across levels “(inter-level view”). These relative frequencies were calculated based on absolute frequencies of coding relations between different influencing factors counted per interview (Table 2) and per paragraph (Table 3), respectively.

Within the data corpus, stylistic characteristics are possible (repetitions or the like). Furthermore, relations may be more or less likely due to similar reference points; for instance, reimbursement as a reference point for "Profitability" {20} and "Economic structure" {32}. Thus, one may assume that, when calculating relative frequencies of coding relations without appropriate weighting, certain influencing factors might be under- or overrepresented.

The here presented approach for weighting is not an appropriate approach in descriptive-statistical sense. It does not take the above-mentioned semantical aspects systematically into account. It is merely an experimental approach in order to check for tendencies regarding argumentative relations.

Table 1: Overview on coding relations

|  |  | | **Number of nodes* (distance = max. one paragraph)***** | | | | **Number of cases per interview; N=15** (distance = max. one paragraph)***** | | | | | **Number of cases per paragraph; N=401** (per paragraph; N=401)***** | | | | |
| --- | --- | --- | --- | --- | --- | --- | --- | --- | --- | --- | --- | --- | --- | --- | --- | --- |
|  |  | | **Absolute frequencies****** | | **Relative frequencies******* | | **Absolute frequencies****** | | | **Relative frequencies******* | | **Absolute frequencies****** | | | **Relative frequencies******* | |
|  |  | | **Possible** | **Factual** | **Unweighted** | **Weighted** | **Possible** | **Factual** | | **Unweighted** | **Weighted** | **Possible** | **Factual** | | **Unweighted** | **Weighted** |
| **Intra-level view** | **Level 1 x Level 1** | | 15 | 3 | 20% | 6% | 225 | 3 | | 1% | 2% | 6015 | 10 | | 0% | 2% |
|  | **Level 2 x Level 2** | | 105 | 51 | 49% | 15% | 1575 | 133 | | 8% | 15% | 42105 | 488 | | 1% | 16% |
|  | **Level 3 x Level 3** | | 28 | 2 | 7% | 2% | 420 | 2 | | 0% | 1% | 11228 | 5 | | 0% | 1% |
|  | **Level 4 x Level 4** | | 15 | 12 | 80% | **25%** | 225 | 47 | | 21% | **37%** | 6015 | 152 | | 3% | **35%** |
| **Inter-level view** | **Level 1 x Level 2** | | 90 | 26 | 29% | 9% | 1350 | 39 | | 3% | 5% | 36090 | 114 | | 0% | 4% |
|  | **Level 1 x Level 3** | | 48 | 2 | 4% | 1% | 720 | 3 | | 0% | 1% | 19248 | 12 | | 0% | 1% |
|  | **Level 1 x Level 4** | | 36 | 11 | 31% | 9% | 540 | 17 | | 3% | 6% | 14436 | 55 | | 0% | 5% |
|  | **Level 2 x Level 3** | | 120 | 28 | 23% | 7% | 1800 | 40 | | 2% | 4% | 48120 | 110 | | 0% | 3% |
|  | **Level 2 x Level 4** | | 90 | 50 | 56% | **17%** | 1350 | 186 | | 14% | **24%** | 36090 | 679 | | 2% | **26%** |
|  | **Level 3 x Level 4** | | 48 | 13 | 27% | 8% | 720 | 23 | | 3% | 6% | 19248 | 74 | | 0% | 5% |
| **Total (intra-level view)** | | | 163 | 68 | 42% | **58%** | 2445 | 185 | | 8% | **61%** | 65363 | 655 | | 1% | **62%** |
| **Total (inter-level view)** | | | 432 | 130 | 30% | 42% | 6480 | 308 | | 5% | 39% | 173232 | 1044 | | 1% | 38% |
| **Total (both views)** | | | 595 | 198 | 33% | 100% | 8925 | | 493 | 6% | 100% | 238595 | | 1699 | 1% | 100% |
| ***** | | A node marks the **point of linkage between two different influencing factors** on the basis of respective code segments. | | | | | | | | | | | | | | |
| ****** | | A case marks the **extent of linkage between two different influencing factors** on the basis of respective code segments. | | | | | | | | | | | | | | |
| ******* | | This distance maximum ensures that only **immediate, thus, strong argumentative relations** are counted. | | | | | | | | | | | | | | |
| ******** | | In terms of **absolute frequencies**, the **number of nodes** can be counted **in intra-level perspective** (Level 1 x Level 1, Level 2 x Level 2, etc.) **versus inter-level perspective** (Level 1 x Level 2, Level 1 x Level 3, etc.) whereby, | | | | | | | | | | | | | | |
|  |  | - the number of **possible nodes** displays the potential linkage, e.g. | | | | | | | | | | | | | | |
|  |  | - - (6*6-6)/2 = 15 possible linkages with regard two intra-level linkages at the patient level, | | | | | | | | | | | | | | |
|  |  | - - 15*6 = 90 possible linkages with regard to inter-level linkages at the patient and provider level, | | | | | | | | | | | | | | |
|  |  | - and the number of **factual nodes** displays the actual linkage, e.g. | | | | | | | | | | | | | | |
|  |  | - - 3 out of 15 possible linkages with regard to intra-level linkages at the patient level were realized, | | | | | | | | | | | | | | |
|  |  | - - 26 out of 90 possible linkages with regard to inter-level linkages at the patient and provider level were realized. | | | | | | | | | | | | | | |
|  |  | Similarly, the **number of cases** can be counted a) **in intra-level perspective** (Level 1 x Level 1, Level 2 x Level 2, etc.) **versus inter-level perspective** (Level 1 x Level 2, Level 1 x Level 3, etc.) and b) **per interview** (15 interviews) **versus per paragraph** (401 paragraphs) whereby, | | | | | | | | | | | | | | |
|  |  | - the number of **possible cases** displays the potential extent, e.g. | | | | | | | | | | | | | | |
|  |  | - - since the data corpus consist of 15 interviews, the potential extent of possible linkages with regard two intra-level linkages at the patient level estimates 15*15 = 225, | | | | | | | | | | | | | | |
|  |  | - - since the data corpus consist of 401 paragraphs, the potential extent of possible linkages with regard two intra-level linkages at the patient level estimates 401*15 = 6015, | | | | | | | | | | | | | | |
|  |  | - and the number of **factual cases** displays the actual extent, e.g. | | | | | | | | | | | | | | |
|  |  | - - 3 out of 225 possible cases with regard to intra-level linkages at the patient level per interview were realized, | | | | | | | | | | | | | | |
|  |  | - - 10 out of 6051 possible linkages with regard to inter-level linkages at the patient and provider level per paragraph were realized. | | | | | | | | | | | | | | |
| ********* | | Based on those absolute frequencies regarding the number of nodes and the number of cases, **relative frequencies** can be calculated. Relative frequencies, thereby, | | | | | | | | | | | | | | |
|  |  | - can be **ranked** in in order to compare the points and extent of linkage in intra-level and inter-level view (see colour highlights in blue), | | | | | | | | | | | | | | |
|  |  | - and can be **weighted**, whereby, | | | | | | | | | | | | | | |
|  |  | - - unweighted relative frequencies depict **ratios of the corresponding absolute frequencies** (i.e. *factual absolute frequency* dived by *possible absolute frequency*), | | | | | | | | | | | | | | |
|  |  | - - and weighted relative frequencies depict **ratios of the corresponding (sum of) unweighted relative frequencies** (i.e. *unweighted relative frequency* as the value to be weighted divided by the *sum of unweighted relative frequencies* as the value for weighting). | | | | | | | | | | | | | | |

Table 2: Absolute frequencies of coding relations per interview

|  | | | **Level 1: Patients** | | | | | | **Level 2: Healthcare providers** | | | | | | | | | | | | | | | **Level 3: CPGs** | | | | | | | | **Level 4: Healthcare system** | | | | | |
| --- | --- | --- | --- | --- | --- | --- | --- | --- | --- | --- | --- | --- | --- | --- | --- | --- | --- | --- | --- | --- | --- | --- | --- | --- | --- | --- | --- | --- | --- | --- | --- | --- | --- | --- | --- | --- | --- |
|  |  |  | **Health literacy** | **Mobility** | **Lifestyle** | **Medication intake** | **Mentality** | **Self-interest** | **Case-related time pressure** | **Constitution of patients** | **Personal situation of patients** | **Relationship with patients** | **Integral healthcare** | **Interprofessional healthcare** | **Stratified healthcare** | **Acceptance of CPGs** | **Evidence orientation** | **Explicit knowledge** | **Implicit knowledge** | **Proactivity** | **Professional responsibility** | **Profitability** | **Prudence** | **Inconsistency** | **Inexpedience** | **Non-binding nature** | **Reliability** | **Abstract nature** | **Ambiguity** | **Incompleteness** | **Ostensible clarity** | **Effort (procedural)** | **Workload (administrative)** | **Economic structures** | **Local structures** | **Stipulated structures** | **Temporal structures** |
|  |  |  | **1** | **2** | **3** | **4** | **5** | **6** | **7** | **8** | **9** | **10** | **11** | **12** | **13** | **14** | **15** | **16** | **17** | **18** | **19** | **20** | **21** | **22** | **23** | **24** | **25** | **26** | **27** | **28** | **29** | **30** | **31** | **32** | **33** | **34** | **35** |
| **Level 1:  Patients** | **1** | **Health literacy** |  |  |  |  |  |  |  |  |  |  |  |  |  |  |  |  |  |  |  |  |  |  |  |  |  |  |  |  |  |  |  |  |  |  |  |
|  | **2** | **Mobility** | 0 |  |  |  |  |  |  |  |  |  |  |  |  |  |  |  |  |  |  |  |  |  |  |  |  |  |  |  |  |  |  |  |  |  |  |
|  | **3** | **Lifestyle** | 1 | 0 |  |  |  |  |  |  |  |  |  |  |  |  |  |  |  |  |  |  |  |  |  |  |  |  |  |  |  |  |  |  |  |  |  |
|  | **4** | **Medication intake** | 0 | 0 | 0 |  |  |  |  |  |  |  |  |  |  |  |  |  |  |  |  |  |  |  |  |  |  |  |  |  |  |  |  |  |  |  |  |
|  | **5** | **Mentality** | 1 | 0 | 0 | 0 |  |  |  |  |  |  |  |  |  |  |  |  |  |  |  |  |  |  |  |  |  |  |  |  |  |  |  |  |  |  |  |
|  | **6** | **Self-interest** | 0 | 0 | 0 | 1 | 0 |  |  |  |  |  |  |  |  |  |  |  |  |  |  |  |  |  |  |  |  |  |  |  |  |  |  |  |  |  |  |
| **Level 2: Healthcare providers** | **7** | **Case-related time pressure** | 0 | 0 | 0 | 0 | 0 | 1 |  |  |  |  |  |  |  |  |  |  |  |  |  |  |  |  |  |  |  |  |  |  |  |  |  |  |  |  |  |
|  | **8** | **Constitution of patients** | 0 | 0 | 0 | 0 | 1 | 0 | 0 |  |  |  |  |  |  |  |  |  |  |  |  |  |  |  |  |  |  |  |  |  |  |  |  |  |  |  |  |
|  | **9** | **Personal situation of patients** | 0 | 0 | 0 | 0 | 0 | 0 | 0 | 0 |  |  |  |  |  |  |  |  |  |  |  |  |  |  |  |  |  |  |  |  |  |  |  |  |  |  |  |
|  | **10** | **Relationship with patients** | 1 | 0 | 1 | 1 | 0 | 1 | 0 | 0 | 0 |  |  |  |  |  |  |  |  |  |  |  |  |  |  |  |  |  |  |  |  |  |  |  |  |  |  |
|  | **11** | **Integral healthcare** | 0 | 0 | 0 | 0 | 0 | 0 | 0 | 0 | 0 | 0 |  |  |  |  |  |  |  |  |  |  |  |  |  |  |  |  |  |  |  |  |  |  |  |  |  |
|  | **12** | **Interprofessional healthcare** | 1 | 1 | 0 | 0 | 0 | 3 | 2 | 0 | 0 | 1 | 1 |  |  |  |  |  |  |  |  |  |  |  |  |  |  |  |  |  |  |  |  |  |  |  |  |
|  | **13** | **Stratified healthcare** | 5 | 0 | 1 | 1 | 2 | 4 | 2 | 5 | 0 | 2 | 2 | 8 |  |  |  |  |  |  |  |  |  |  |  |  |  |  |  |  |  |  |  |  |  |  |  |
|  | **14** | **Acceptance of CPGs** | 0 | 0 | 0 | 0 | 0 | 0 | 0 | 0 | 1 | 0 | 0 | 0 | 1 |  |  |  |  |  |  |  |  |  |  |  |  |  |  |  |  |  |  |  |  |  |  |
|  | **15** | **Evidence orientation** | 3 | 0 | 0 | 0 | 0 | 1 | 1 | 3 | 0 | 2 | 2 | 3 | 10 | 0 |  |  |  |  |  |  |  |  |  |  |  |  |  |  |  |  |  |  |  |  |  |
|  | **16** | **Explicit knowledge** | 1 | 0 | 0 | 0 | 0 | 1 | 3 | 2 | 0 | 1 | 0 | 4 | 6 | 1 | 4 |  |  |  |  |  |  |  |  |  |  |  |  |  |  |  |  |  |  |  |  |
|  | **17** | **Implicit knowledge** | 2 | 0 | 1 | 0 | 0 | 1 | 0 | 1 | 0 | 2 | 1 | 1 | 7 | 0 | 3 | 1 |  |  |  |  |  |  |  |  |  |  |  |  |  |  |  |  |  |  |  |
|  | **18** | **Proactivity** | 0 | 0 | 0 | 0 | 0 | 0 | 0 | 1 | 0 | 0 | 0 | 0 | 2 | 0 | 1 | 1 | 0 |  |  |  |  |  |  |  |  |  |  |  |  |  |  |  |  |  |  |
|  | **19** | **Professional responsibility** | 0 | 0 | 0 | 0 | 0 | 1 | 1 | 0 | 0 | 0 | 0 | 0 | 1 | 0 | 0 | 1 | 0 | 0 |  |  |  |  |  |  |  |  |  |  |  |  |  |  |  |  |  |
|  | **20** | **Profitability** | 0 | 0 | 1 | 0 | 0 | 1 | 1 | 1 | 0 | 0 | 0 | 6 | 7 | 0 | 6 | 5 | 0 | 1 | 1 |  |  |  |  |  |  |  |  |  |  |  |  |  |  |  |  |
|  | **21** | **Prudence** | 1 | 0 | 0 | 0 | 0 | 1 | 0 | 0 | 0 | 1 | 2 | 2 | 5 | 0 | 3 | 0 | 1 | 0 | 0 | 0 |  |  |  |  |  |  |  |  |  |  |  |  |  |  |  |
| **Level 3: CPGs** | **22** | **Inconsistency** | 0 | 0 | 0 | 0 | 0 | 0 | 0 | 0 | 0 | 0 | 0 | 0 | 0 | 1 | 1 | 0 | 0 | 0 | 0 | 0 | 0 |  |  |  |  |  |  |  |  |  |  |  |  |  |  |
|  | **23** | **Inexpedience** | 0 | 0 | 0 | 0 | 0 | 2 | 0 | 1 | 0 | 0 | 0 | 2 | 3 | 0 | 2 | 2 | 0 | 1 | 0 | 5 | 1 | 0 |  |  |  |  |  |  |  |  |  |  |  |  |  |
|  | **24** | **Non-binding nature** | 0 | 0 | 0 | 0 | 0 | 0 | 0 | 0 | 0 | 0 | 0 | 0 | 0 | 0 | 0 | 0 | 1 | 0 | 0 | 0 | 0 | 0 | 0 |  |  |  |  |  |  |  |  |  |  |  |  |
|  | **25** | **Reliability** | 0 | 0 | 0 | 0 | 0 | 0 | 0 | 0 | 0 | 0 | 0 | 0 | 1 | 2 | 0 | 0 | 0 | 0 | 0 | 0 | 0 | 0 | 0 | 0 |  |  |  |  |  |  |  |  |  |  |  |
|  | **26** | **Abstract nature** | 1 | 0 | 0 | 0 | 0 | 0 | 0 | 0 | 0 | 1 | 1 | 1 | 1 | 0 | 2 | 0 | 1 | 0 | 0 | 1 | 2 | 0 | 1 | 0 | 0 |  |  |  |  |  |  |  |  |  |  |
|  | **27** | **Ambiguity** | 0 | 0 | 0 | 0 | 0 | 0 | 0 | 0 | 0 | 0 | 0 | 0 | 1 | 0 | 0 | 0 | 1 | 0 | 0 | 0 | 0 | 0 | 0 | 0 | 0 | 0 |  |  |  |  |  |  |  |  |  |
|  | **28** | **Incompleteness** | 0 | 0 | 0 | 0 | 0 | 0 | 0 | 0 | 0 | 0 | 0 | 0 | 0 | 0 | 0 | 0 | 0 | 0 | 0 | 1 | 0 | 0 | 0 | 0 | 0 | 0 | 0 |  |  |  |  |  |  |  |  |
|  | **29** | **Ostensible clarity** | 0 | 0 | 0 | 0 | 0 | 0 | 0 | 0 | 0 | 0 | 1 | 0 | 1 | 0 | 1 | 0 | 0 | 0 | 0 | 1 | 0 | 0 | 1 | 0 | 0 | 0 | 0 | 0 |  |  |  |  |  |  |  |
| **Level 4: Healthcare system** | **30** | **Effort (procedural)** | 0 | 0 | 0 | 0 | 1 | 0 | 1 | 3 | 0 | 0 | 0 | 3 | 7 | 0 | 8 | 4 | 0 | 2 | 0 | 8 | 0 | 0 | 4 | 0 | 0 | 1 | 0 | 0 | 0 |  |  |  |  |  |  |
|  | **31** | **Workload (administrative)** | 0 | 0 | 0 | 0 | 0 | 0 | 0 | 0 | 0 | 0 | 0 | 2 | 1 | 0 | 0 | 0 | 0 | 0 | 0 | 0 | 0 | 0 | 1 | 0 | 0 | 0 | 0 | 0 | 0 | 0 |  |  |  |  |  |
|  | **32** | **Economic structures** | 0 | 0 | 0 | 0 | 0 | 2 | 0 | 2 | 0 | 0 | 0 | 6 | 7 | 1 | 5 | 5 | 0 | 1 | 2 | 10 | 0 | 0 | 5 | 0 | 0 | 1 | 0 | 0 | 1 | 8 | 2 |  |  |  |  |
|  | **33** | **Local structures** | 2 | 0 | 1 | 0 | 1 | 2 | 1 | 1 | 0 | 0 | 0 | 5 | 9 | 0 | 3 | 5 | 1 | 0 | 2 | 5 | 2 | 0 | 2 | 0 | 0 | 0 | 0 | 1 | 0 | 3 | 0 | 4 |  |  |  |
|  | **34** | **Stipulated structures** | 0 | 0 | 2 | 0 | 0 | 0 | 0 | 1 | 0 | 0 | 0 | 4 | 5 | 3 | 2 | 2 | 0 | 2 | 1 | 6 | 1 | 0 | 1 | 0 | 2 | 0 | 0 | 1 | 0 | 0 | 1 | 3 | 5 |  |  |
|  | **35** | **Temporal structures** | 0 | 1 | 1 | 0 | 1 | 3 | 5 | 1 | 0 | 2 | 0 | 10 | 10 | 0 | 4 | 5 | 1 | 0 | 2 | 5 | 2 | 0 | 2 | 0 | 0 | 1 | 0 | 0 | 0 | 4 | 2 | 5 | 6 | 4 |  |

Table 3: Absolute frequencies of coding relations per paragraph

|  | | | **Level 1: Patients** | | | | | | **Level 2: Healthcare providers** | | | | | | | | | | | | | | | **Level 3: CPGs** | | | | | | | | **Level 4: Healthcare system** | | | | | |
| --- | --- | --- | --- | --- | --- | --- | --- | --- | --- | --- | --- | --- | --- | --- | --- | --- | --- | --- | --- | --- | --- | --- | --- | --- | --- | --- | --- | --- | --- | --- | --- | --- | --- | --- | --- | --- | --- |
|  |  |  | **Health literacy** | **Mobility** | **Lifestyle** | **Medication intake** | **Mentality** | **Self-interest** | **Case-related time pressure** | **Constitution of patients** | **Personal situation of patients** | **Relationship with patients** | **Integral healthcare** | **Interprofessional healthcare** | **Stratified healthcare** | **Acceptance of CPGs** | **Evidence orientation** | **Explicit knowledge** | **Implicit knowledge** | **Proactivity** | **Professional responsibility** | **Profitability** | **Prudence** | **Inconsistency** | **Inexpedience** | **Non-binding nature** | **Reliability** | **Abstract nature** | **Ambiguity** | **Incompleteness** | **Ostensible clarity** | **Effort (procedural)** | **Workload (administrative)** | **Economic structures** | **Local structures** | **Stipulated structures** | **Temporal structures** |
|  |  |  | **1** | **2** | **3** | **4** | **5** | **6** | **7** | **8** | **9** | **10** | **11** | **12** | **13** | **14** | **15** | **16** | **17** | **18** | **19** | **20** | **21** | **22** | **23** | **24** | **25** | **26** | **27** | **28** | **29** | **30** | **31** | **32** | **33** | **34** | **35** |
| **Level 1:  Patients** | **1** | **Health literacy** |  |  |  |  |  |  |  |  |  |  |  |  |  |  |  |  |  |  |  |  |  |  |  |  |  |  |  |  |  |  |  |  |  |  |  |
|  | **2** | **Mobility** | 0 |  |  |  |  |  |  |  |  |  |  |  |  |  |  |  |  |  |  |  |  |  |  |  |  |  |  |  |  |  |  |  |  |  |  |
|  | **3** | **Lifestyle** | 2 | 0 |  |  |  |  |  |  |  |  |  |  |  |  |  |  |  |  |  |  |  |  |  |  |  |  |  |  |  |  |  |  |  |  |  |
|  | **4** | **Medication intake** | 0 | 0 | 0 |  |  |  |  |  |  |  |  |  |  |  |  |  |  |  |  |  |  |  |  |  |  |  |  |  |  |  |  |  |  |  |  |
|  | **5** | **Mentality** | 6 | 0 | 0 | 0 |  |  |  |  |  |  |  |  |  |  |  |  |  |  |  |  |  |  |  |  |  |  |  |  |  |  |  |  |  |  |  |
|  | **6** | **Self-interest** | 0 | 0 | 0 | 2 | 0 |  |  |  |  |  |  |  |  |  |  |  |  |  |  |  |  |  |  |  |  |  |  |  |  |  |  |  |  |  |  |
| **Level 2: Healthcare providers** | **7** | **Case-related time pressure** | 0 | 0 | 0 | 0 | 0 | 2 |  |  |  |  |  |  |  |  |  |  |  |  |  |  |  |  |  |  |  |  |  |  |  |  |  |  |  |  |  |
|  | **8** | **Constitution of patients** | 0 | 0 | 0 | 0 | 2 | 0 | 0 |  |  |  |  |  |  |  |  |  |  |  |  |  |  |  |  |  |  |  |  |  |  |  |  |  |  |  |  |
|  | **9** | **Personal situation of patients** | 0 | 0 | 0 | 0 | 0 | 0 | 0 | 0 |  |  |  |  |  |  |  |  |  |  |  |  |  |  |  |  |  |  |  |  |  |  |  |  |  |  |  |
|  | **10** | **Relationship with patients** | 2 | 0 | 3 | 2 | 0 | 2 | 0 | 0 | 0 |  |  |  |  |  |  |  |  |  |  |  |  |  |  |  |  |  |  |  |  |  |  |  |  |  |  |
|  | **11** | **Integral healthcare** | 0 | 0 | 0 | 0 | 0 | 0 | 0 | 0 | 0 | 0 |  |  |  |  |  |  |  |  |  |  |  |  |  |  |  |  |  |  |  |  |  |  |  |  |  |
|  | **12** | **Interprofessional healthcare** | 2 | 2 | 0 | 0 | 0 | 7 | 4 | 0 | 0 | 2 | 2 |  |  |  |  |  |  |  |  |  |  |  |  |  |  |  |  |  |  |  |  |  |  |  |  |
|  | **13** | **Stratified healthcare** | 23 | 0 | 3 | 2 | 9 | 14 | 6 | 18 | 0 | 10 | 4 | 30 |  |  |  |  |  |  |  |  |  |  |  |  |  |  |  |  |  |  |  |  |  |  |  |
|  | **14** | **Acceptance of CPGs** | 0 | 0 | 0 | 0 | 0 | 0 | 0 | 0 | 2 | 0 | 0 | 0 | 2 |  |  |  |  |  |  |  |  |  |  |  |  |  |  |  |  |  |  |  |  |  |  |
|  | **15** | **Evidence orientation** | 8 | 0 | 0 | 0 | 0 | 2 | 4 | 12 | 0 | 4 | 4 | 10 | 87 | 0 |  |  |  |  |  |  |  |  |  |  |  |  |  |  |  |  |  |  |  |  |  |
|  | **16** | **Explicit knowledge** | 2 | 0 | 0 | 0 | 0 | 6 | 6 | 5 | 0 | 2 | 0 | 8 | 22 | 2 | 20 |  |  |  |  |  |  |  |  |  |  |  |  |  |  |  |  |  |  |  |  |
|  | **17** | **Implicit knowledge** | 5 | 0 | 2 | 0 | 0 | 2 | 0 | 2 | 0 | 5 | 6 | 6 | 35 | 0 | 12 | 2 |  |  |  |  |  |  |  |  |  |  |  |  |  |  |  |  |  |  |  |
|  | **18** | **Proactivity** | 0 | 0 | 0 | 0 | 0 | 0 | 0 | 2 | 0 | 0 | 0 | 0 | 5 | 0 | 5 | 3 | 0 |  |  |  |  |  |  |  |  |  |  |  |  |  |  |  |  |  |  |
|  | **19** | **Professional responsibility** | 0 | 0 | 0 | 0 | 0 | 2 | 2 | 0 | 0 | 0 | 0 | 0 | 2 | 0 | 0 | 2 | 0 | 0 |  |  |  |  |  |  |  |  |  |  |  |  |  |  |  |  |  |
|  | **20** | **Profitability** | 0 | 0 | 2 | 0 | 0 | 2 | 2 | 3 | 0 | 0 | 0 | 15 | 24 | 0 | 31 | 14 | 0 | 3 | 2 |  |  |  |  |  |  |  |  |  |  |  |  |  |  |  |  |
|  | **21** | **Prudence** | 2 | 0 | 0 | 0 | 0 | 4 | 0 | 0 | 0 | 2 | 4 | 4 | 13 | 0 | 10 | 0 | 6 | 0 | 0 | 0 |  |  |  |  |  |  |  |  |  |  |  |  |  |  |  |
| **Level 3: CPGs** | **22** | **Inconsistency** | 0 | 0 | 0 | 0 | 0 | 0 | 0 | 0 | 0 | 0 | 0 | 0 | 0 | 2 | 2 | 0 | 0 | 0 | 0 | 0 | 0 |  |  |  |  |  |  |  |  |  |  |  |  |  |  |
|  | **23** | **Inexpedience** | 0 | 0 | 0 | 0 | 0 | 10 | 0 | 2 | 0 | 0 | 0 | 6 | 7 | 0 | 8 | 5 | 0 | 2 | 0 | 11 | 2 | 0 |  |  |  |  |  |  |  |  |  |  |  |  |  |
|  | **24** | **Non-binding nature** | 0 | 0 | 0 | 0 | 0 | 0 | 0 | 0 | 0 | 0 | 0 | 0 | 0 | 0 | 0 | 0 | 4 | 0 | 0 | 0 | 0 | 0 | 0 |  |  |  |  |  |  |  |  |  |  |  |  |
|  | **25** | **Reliability** | 0 | 0 | 0 | 0 | 0 | 0 | 0 | 0 | 0 | 0 | 0 | 0 | 2 | 4 | 0 | 0 | 0 | 0 | 0 | 0 | 0 | 0 | 0 | 0 |  |  |  |  |  |  |  |  |  |  |  |
|  | **26** | **Abstract nature** | 2 | 0 | 0 | 0 | 0 | 0 | 0 | 0 | 0 | 2 | 2 | 2 | 6 | 0 | 7 | 0 | 9 | 0 | 0 | 2 | 4 | 0 | 2 | 0 | 0 |  |  |  |  |  |  |  |  |  |  |
|  | **27** | **Ambiguity** | 0 | 0 | 0 | 0 | 0 | 0 | 0 | 0 | 0 | 0 | 0 | 0 | 4 | 0 | 0 | 0 | 2 | 0 | 0 | 0 | 0 | 0 | 0 | 0 | 0 | 0 |  |  |  |  |  |  |  |  |  |
|  | **28** | **Incompleteness** | 0 | 0 | 0 | 0 | 0 | 0 | 0 | 0 | 0 | 0 | 0 | 0 | 0 | 0 | 0 | 0 | 0 | 0 | 0 | 3 | 0 | 0 | 0 | 0 | 0 | 0 | 0 |  |  |  |  |  |  |  |  |
|  | **29** | **Ostensible clarity** | 0 | 0 | 0 | 0 | 0 | 0 | 0 | 0 | 0 | 0 | 2 | 0 | 3 | 0 | 2 | 0 | 0 | 0 | 0 | 3 | 0 | 0 | 3 | 0 | 0 | 0 | 0 | 0 |  |  |  |  |  |  |  |
| **Level 4: Healthcare system** | **30** | **Effort (procedural)** | 0 | 0 | 0 | 0 | 3 | 0 | 2 | 11 | 0 | 0 | 0 | 6 | 23 | 0 | 30 | 11 | 0 | 6 | 0 | 26 | 0 | 0 | 10 | 0 | 0 | 2 | 0 | 0 | 0 |  |  |  |  |  |  |
|  | **31** | **Workload (administrative)** | 0 | 0 | 0 | 0 | 0 | 0 | 0 | 0 | 0 | 0 | 0 | 7 | 3 | 0 | 0 | 0 | 0 | 0 | 0 | 0 | 0 | 0 | 4 | 0 | 0 | 0 | 0 | 0 | 0 | 0 |  |  |  |  |  |
|  | **32** | **Economic structures** | 0 | 0 | 0 | 0 | 0 | 8 | 0 | 8 | 0 | 0 | 0 | 19 | 32 | 2 | 29 | 18 | 0 | 6 | 4 | 47 | 0 | 0 | 27 | 0 | 0 | 2 | 0 | 0 | 3 | 23 | 7 |  |  |  |  |
|  | **33** | **Local structures** | 4 | 0 | 6 | 0 | 2 | 5 | 5 | 2 | 0 | 0 | 0 | 11 | 49 | 0 | 9 | 11 | 2 | 0 | 4 | 15 | 8 | 0 | 5 | 0 | 0 | 0 | 0 | 3 | 0 | 9 | 0 | 16 |  |  |  |
|  | **34** | **Stipulated structures** | 0 | 0 | 7 | 0 | 0 | 0 | 0 | 2 | 0 | 0 | 0 | 10 | 21 | 7 | 5 | 5 | 0 | 4 | 2 | 24 | 2 | 0 | 2 | 0 | 5 | 0 | 0 | 3 | 0 | 0 | 2 | 10 | 18 |  |  |
|  | **35** | **Temporal structures** | 0 | 2 | 2 | 0 | 2 | 14 | 21 | 3 | 0 | 4 | 0 | 61 | 49 | 0 | 16 | 12 | 2 | 0 | 4 | 13 | 6 | 0 | 6 | 0 | 0 | 2 | 0 | 0 | 0 | 12 | 4 | 13 | 26 | 12 |  |
