# Supplementary material for: Factors influencing adherence to clinical practice guidelines in patients with suspected chronic coronary syndrome: a qualitative interview study in the ambulatory care sector in Germany
Source: BMC Health Serv Res. 2023 Jun 20;23:655. doi: 10.1186/s12913-023-09587-1 (PMC10283181; doi:10.1186/s12913-023-09587-1)
Supplement: Supplementary file 6 — Supplementary Material 6 [file 12913_2023_9587_MOESM6_ESM.docx]

Code summaries

This Additional File provides code summaries of the identified 35 factors that may influence adherence to NVL-CAD and ESC-CCS are provided. These consist of

- the **original categorial term** in German,
- the **code-alias** that was created for a common categorial understanding between the two coders during the coding process,
- a list of **focus themes** that were brought up by the respondents in the context of the identified influencing factor,
- and **an exemplary original code segment** that illustrates the identified influencing factor.

# Health Literacy (in German: *Gesundheitskompetenz*)

*Code-alias: Patient ability to handle health-related information*

## Focus topics

- Expression
- Health awareness
- Disease awareness

## Example quote

**If patients have sufficient awareness of their disease, it could facilitate adherence to CPGs regarding the use of ICA in patients with highly presumed CCS:** "I would tend to say: if someone has already had a history of CCS that was symptomatic for them, then I would naturally try to find out whether the symptoms that now bring them back to me are comparable with the complaints they have already had. And if I know that they have already had similar complaints or have already had a stent or something, then I am of course quicker and more generous in clarifying them invasively." (B11, CA)

# Mobility (in German: *Mobilität*)

*Code-alias: Patient capacity to be mobile*

## Focus topics

- Capacity to travel to healthcare facilities

## Example quote

**If patients have the capacity to travel to healthcare facilities, it could facilitate adherence to CPGs regarding the use of ICA in patients with highly presumed CCS:** "Well, as so often: it depends. So, I cannot say in general/ somehow give a general time frame. But related to me now, for example: in the end, I work together with two ICA laboratories in this medium-sized city. One is directly on site in the clinic. The waiting times are a bit longer there. And there is a clinic, specialised cardiac clinic, half an hour’s drive away, right? It is very fast there, right? Within days. So, depending on my feeling regarding how urgently this examination should take place, I send the patients there or there. A certain mobility of course presupposed, if they were to go to this clinic that is half an hour away." (B02, GP)

# Lifestyle (in German: *Lebensstil*)

*Code-alias: Lifestyle-related behaviours of patients*

## Focus topics

- Lifestyle non-compliance

## Example quote

**Lifestyle non-compliance on behalf of patients could inhibit adherence to CPGs regarding lifestyle changes:** "Those are the ones who then have a reinfarction and are operated on *again* and get another stent and so on and so forth. And then you really ask yourself: what is the point? It is a bit like Loriot. With asthma it would be catastrophic, right? First, I take the spray so that I can breathe. And then, when I can breathe, I smoke my cigar because it tastes so good. Great. But it is no different with CCS: with its nicotine content, a cigarette makes the blood vessels narrow for six hours and promotes what we are otherwise trying to stop." (B10, GP)

# Medication intake (in German: *Medikamenteneinnahme*)

*Code-alias: Medication-related behaviours of patients*

## Focus topics

- Medication (non-)compliance

## Example quote

**Medication non-compliance on behalf of patients could inhibit adherence to CPGs regarding medication:** "Yes, physicians who have been involved for a little longer know that compliance varies greatly […]. And it takes a lot of talking and a lot of convincing to keep the patients in line. *Particularly* regarding the anticoagulant drugs after the final treatment that are extremely important. And that means a lot of work. […] Knowing full well, however, that it is often forgotten or not done for a variety of reasons. And the compliance reasons range from such banal things as ‘I do not have any money for the co-payment’ – that actually *still* exists – to ‘I do not take more than two tablets’ to ‘I simply refuse it because it does not do me any good or I have side effects.’ Or also fears that something will happen with it, with the medication in the body." (B05, GP)

# Mentality (in German: *Mentalität*)

*Code-alias: Patients’ attitudes towards healthcare*

## Focus topics

- Anxiousness
- Open-mindedness
- Dissimulation
- Impatience

## Example quote

**If patients are impatient, it could inhibit adherence to CPGs regarding the use of stress echocardiography as NIT in patients with intermediately presumed CCS:** "And if they are young, for example, and you say you are going to do it on an ergometer, then you cannot *position* them properly, then you really have to concentrate a *lot.* The patients are sitting next to you and want to know immediately what is going on. I believe that a radiologist or the colleagues from the CMRI have an advantage if they can look at the images again without the patient sitting next to them. And/ Yes, these are the limitations kind of." (B13, CA)

# Self-interest (in German: *Eigeninteresse*)

*Code-alias: Patients’ intentions regarding healthcare*

## Focus topics

- Preferences regarding physicians
- Preferences regarding diagnostic confirmation
- Preferences regarding self-financing
- Preferences regarding social law aspects

## Example quote

**If patients have preferences regarding diagnostic confirmation, it could inhibit adherence to CPGs regarding the use of ICA in patients with intermediately presumed CCS:** "Compliance or also: what does the patient want? They ask me very *specifically*. Or when I say to them: ‘Well, it could well be that what you have there is CCS. Of course, you should stop smoking and this and that.’ But they will also ask me, ‘Yes, is there any other way/ What do I have to do to clarify this further?’ or ‘How much am I at risk of a heart attack?’ And then, at some point, you get to the point where you do decide to do a diagnostic/ Or: for ICA to confirm the diagnosis." (B12, GP)

# Case-related time pressure (in German: Fallbezogener *Zeitdruck*)

*Code-alias: Healthcare providers’ consideration of the urgency of individual patients*

## Focus topics

- Healthcare providers’ consideration of ICA due to time pressure
- Healthcare providers’ consideration of inpatient admission due to time pressure

## Example quote

**If healthcare providers considerate inpatient admission due to time pressure, it could influence adherence to CPGs regarding basic diagnostic decisions:** "And on the one hand we are told, ‘Do not always send patients to the hospital.’ Okay, I understand that. But on the other hand, you have three, four months of waiting time for an appointment with a CA. And something does not fit there, right? There is a big shortcoming already. First of all, I am not supposed to admit the patient as an inpatient, okay. But on the other hand, I cannot leave the patient lying around for three months just to wait for the CA." (B07, GP)

# Constitution of patients (in German: *Konstitution von Patient:innen*)

*Code-alias: Healthcare providers’ consideration of the mental and physical condition of individual patients*

## Focus topics

- Consideration of the mental condition of patients
- Consideration of the physical condition of patients

## Example quote

**If healthcare providers considerate the physical condition of patients, it could complicate or facilitate adherence to CPGs regarding the use of stress echocardiography as NIT in patients with intermediately presumed CCS:** "The patient must offer the prerequisites for it. So, it is different whether you examine someone who is athletic, wiry, and of normal weight, or whether you examine someone who is a heavy smoker with overinflated lungs, which then always push in front of the heart, or someone who is very overweight. Where you cannot even properly depict the heart with the ultrasound, right? And unfortunately, these patients who have this exact heart disease are often long-time smokers or very overweight. And to be honest, they often cannot be examined well using stress echocardiography, right? Although the procedure is very good, it has its limitations" (B08, CA)

# Personal situation of patients (in German: *Persönliche Situation von Patient:innen*)

*Code-alias: Healthcare providers’ consideration of the social context of individual patients*

## Focus topics

- Considerations against the background of patients’ professional background

## Example quote

**If healthcare providers considerate patients’ professional background, it could inhibit adherence to CPGs regarding basic diagnostic decisions:** "But on the other hand, I think that being a physician also means knowing the range of possibilities and, in the end, breaking it a bit down to the person in front of you. And I will put it this way: if someone is a bus driver in public transport, it is *very* important for me to have this clarified. It is, perhaps, different from someone who is only moving in their domesticity at an already significantly reduced level or something, right? So, I think such […] secondary information plays a role in the end, too, right?" (B11, CA)

# Relationship with patients (in German: *Beziehung zu Patient:innen)*

*Code-alias: Healthcare providers’ consideration of relations with individual patients*

## Focus topics

- Considerations against the background of long-term relationships with patients
- Considerations against the background of personal relationships with patients

## Example quote

**If healthcare providers considerate personal relationships with patients, it could influence adherence to CPGs regarding basic diagnostic decisions:** "So, it plays/ Yes, I think. So, I do not want to argue against CPGs like that. That would be a/ Then I would not have expressed myself correctly. It is more a matter of: so, you/ […] Yes, how should I put it? It is always a mixture of what can be objectified and what kind of relationship you subjectively built up." (B11, CA)

# Integral healthcare (in German: *Integrale Versorgung*)

*Code-alias: Psychosomatic-oriented approaches used by healthcare providers*

## Focus topics

- Weighing of psychogenic symptom causes

## Example quote

**If healthcare providers weigh psychogenic symptom causes, it stands in line with guideline recommendations regarding a differentiated medical history:** "Or one says: ‘So, this is now rather/’ For example: ‘The whole thing has more psychosomatic origins.’" (B09, GP)

# Interprofessional healthcare (in German: *Interprofessionelle Versorgung*)

*Code-alias: Cooperative approaches used by healthcare providers*

## Focus topics

- Cooperation at the level of administration
- Cooperation at the level of communication
- Cooperation at the level of CPG development
- Cooperation at the level of contracts
- Cooperation in local perspective
- Cooperation in temporal perspective

## Example quote

**If healthcare providers cooperate in temporal perspective, it could facilitate adherence to CPGs regarding the use of MPS as NIT in patients with intermediately presumed CCS:** "And it is still important that you simply have a good network, that you know: okay, if this and that is the case, then I can call there quickly. And if I have announced the patient, then they will be triaged at the central emergency room, will be seen quickly, and will be treated quickly. And if in a radiology department, which – at least here in my area – are also affiliated with hospitals/ If there was something highly conspicuous, for example, in case of a MPS, then they would immediately pass them on to the hospital, right? And I know that that is how it works. And it is also good that I always have diagnostics quite quickly from my side. I cannot complain now, right?" (B04, GP)

# Stratified healthcare (in German: *Stratifizierende Versorgung*)

*Code-alias: Schematic approaches used by healthcare providers*

## Focus topics

- Estimation of procedures’ significance
- Estimation of pre-test probabilities
- Differentiation between de novo CCS versus known CCS
- Differentiation regarding urgency of CCS
- Proceeding in recommended path logics

## Example quote

**If healthcare providers’ estimate pre-test probabilities and proceed in recommended path logics, it stands in line with guideline recommendations regarding a differentiated medical history:** "Classic example: the twenty-year-old woman who says, ‘I once had a stitch in my breast.’ They do not have CCS. They have less than a five percent chance of having CCS. You do not need to do any further diagnostics. You should just do an echocardiography and say goodbye. And now I am doing the opposite: the eighty-year-old smoker and diabetic who says, ‘I have such a heavy stone on my chest when I exert myself.’ You do not need to do any more ergometry, you do not need to do any more stress electrocardiography. You can send them straight to extended diagnostics, in the case I just mentioned: ICA." (B01, CA)

# Acceptance of CPGs (in German: *Akzeptanz von Leitlinien*)

*Code-alias: Healthcare providers’ attitudes towards CPGs*

## Focus topics

- Endorsement of CPG content
- Endorsement of CPGs as maxims of action

## Example quote

**If healthcare providers endorse CPGs as maxims of action, it could facilitate adherence to CPGs regarding basic diagnostic decisions:** "No, no. But as a physician, you have to adhere to CPGs, right? But as a physician, you have to follow CPGs, right? So, CPGs are the standard for doctors’ actions, right? That is indeed so, right?" (B08, CA)

# Evidence orientation (in German: *Evidenzorientierung*)

*Code-alias: Healthcare providers’ attitudes towards scientific evidence*

## Focus topics

- Recognition of the informative value of procedures
- Recognition of the potential outcomes of procedures
- Recognition of the potential risks of procedures

## Example quote

**If healthcare providers recognise the informative value of procedures, it could facilitate adherence to CPGs regarding the use of exercise electrocardiographies as a differential diagnostic instrument:** "Exactly. And in prevention or in diagnostic clarification, ergometry still has a great significance. It is just that the predictive probability – and we know this – is ultimately not as (sighs) specific and accurate as we would like." (B05, GP)

# Explicit knowledge (in German: *Explizites Wissen*)

*Code-alias: Healthcare providers’ manifest knowledge*

## Focus topics

- Professional knowledge as physicians
- Professional knowledge regarding the German DMP on CHD
- Professional knowledge of relevant CPGs on CCS
- Professional knowledge to lead coronary exercise groups
- Professional knowledge of diagnostic procedures

## Example quote

**If healthcare providers decide due to professional knowledge of diagnostic procedures, it could influence adherence to CPGs regarding the use of (stress) electrocardiographies and echocardiographies as differential diagnostic tools:** "Now I am an internist who can also do a bit of echocardiography and is perhaps also a bit more confident in some things: in the evaluation of electrocardiographies or also ergometry. However, there is exactly this problem in the field of family medicine, that there are colleagues who perform a resting electrocardiography, a troponin rapid test – and do not even do stress electrocardiography, let alone echocardiography or anything else – and then become *uncertain* in such a situation regarding (laughs) missing or the question: have I possibly missed a preventable course here? In terms of: sudden cardiac death or acute coronary syndrome." (B12, GP).

# Implicit knowledge (in German: *Implizites Wissen*)

*Code-alias: Healthcare providers’ latent knowledge*

## Focus topics

- Knowledge based on experience
- Knowledge based on discretion
- Knowledge based on expertise
- Knowledge based on intuition
- Knowledge based on (gut) feeling
- Knowledge based on a ‘tingling in the neck’
- Knowledge based on a ‘sixth sense’

## Example quote

**If healthcare providers’ decide due to knowledge based on experience, it could influence adherence to CPGs regarding basic diagnostic decisions:** "Whereby I have to say for myself now: Sure, there are the most different scores that have been initiated as an aid by *national* societies, by *European* societies. If you have been at it for a while/ So, I do not look at the tables now, I have to say, right? But I really do it more according to other criteria and then go according to my experience, according to my feeling, in which direction it goes. […] For me personally, I do not look at the numbers and see from a score of so-and-so percent: then I will go down the path to ICA, right? And I think that is what many of my colleagues do. That it is more of an aid, but not a fixed ritual that you have to follow." (B02, GP)

# Proactivity (in German: *Proaktivität*)

*Code-alias: Healthcare providers’ attitudes towards mobilisation of services*

## Focus topics

- (Non)-readiness for procedure mobilisation

## Example quote

**If healthcare providers show non-readiness for procedure mobilisation, it could inhibit adherence to CPGs regarding the use of (stress) electrocardiographies and echocardiographies as differential diagnostic tools:** "As I said, if you could implement this on your own, it would be worth its weight in gold, right? So, it really does not take much to do basic cardiac diagnostics or basic diagnostics. So, that is/ As I said: the electrocardiography, the echocardiography and the stress electrocardiography. If you go through these examinations, then you really have a lot in hand. So that you can decide then, right? If you *could* already implement this – which is actually not much, right? –, then you would already be a few steps further." (B07, GP)

# Professional responsibility (in German: *Professionelle Verantwortung*)

*Code-alias: Healthcare providers’ intentions regarding conflicting interests*

## Focus topics

- Conflicting of interest in economic terms
- Conflicting of interest in organisational terms
- Conflicting of interest in role-specific terms
- Conflicting of interest in contractual terms

## Example quote

**If healthcare providers have conflicting interests in role-specific terms, it could inhibit adherence to CPGs regarding basic diagnostic decisions:** "In the clinic, people are annoyed that too many patients are admitted, and then they say, ‘We did not need to clarify them as an inpatient at all.’ However, the GP then again is under pressure: ‘Hmm <pondering>. Could there be a significant CCS behind this after all? What do I do if something happens now?’ On the health insurers’ side, in turn, outpatient physicians get the pressure: ‘Just do not admit too many.’" (B07, GP)

# Profitability (in German: *Rentabilität*)

*Code-alias: Healthcare providers’ intentions regarding economic balancing*

## Focus topics

- Consideration of cost expenditures
- Consideration of revenue

## Example quote

**If healthcare providers considerate cost expenditures and revenue, it could influence adherence to CPGs regarding the use of stress echocardiography as NIT in patients with intermediately presumed CCS:** "And/ Well, and if specialists then purchase expensive devices, then they must also somehow pay for themselves again. (Laughs). Therefore, I think it is sometimes not so easy to determine whether the indication for the examination was purely medical or whether there are other reasons, right? And/ Yes, so I *hardly* ever see, for example, that a stress echocardiography is somehow done. With private patients: yes, there it is done quickly." (B14, GP)

# Prudence (in German: *Vorsicht*)

*Code-alias: Healthcare providers’ attitudes towards safe decision-making*

## Focus topics

- Decisions due to caution

## Example quote

**If healthcare providers decide due to caution, it could influence adherence to CPGs regarding the use of ICA in patients with intermediately presumed CCS:** "I think we do too much safeguarding invasive diagnostics in practice: partly out of fear of complication." (B12, GP)

# Inconsistency (in German: *Inkonsistenz*)

*Code-alias: Contradictory recommendations in CPGs (content-related characteristic)*

## Focus topics

- Contradictory recommendations regarding care coordination

## Example quote

**Contradictory recommendations may be complicated to adhere to:** "Okay, now I am going to say jokingly: do you know page 94 on the bottom right? It is not page 94, I think it is the bottom of page 94 in the patient edition. There it says the following sentence: the symptom-free patient should *not* receive regular special examinations. Somehow three pages before it says: for the patient with myocardial infarction, previous myocardial infarction, or diabetes mellitus, the further procedure should be planned in consultation with the GP and the CA." (B01, CA)

# Inexpedience (in German: *Nicht-Realisierbarkeit*)

*Code-alias: Non-transferable recommendations in CPGs (content-related characteristic)*

## Focus topics

- Non-transferable recommendations due to origin versus application of CPGs
- Non-transferable recommendations due to creation versus application of CPGs

## Example quote

**Non-transferable recommendations may be complicated to adhere to:** "The problem of CPGs is sometimes that it somehow takes years to create them. And in this moment – when they are finished – they are already outdated again, right? So, that is unfortunately the case with many things." (B14, GP)

# Non-binding nature (in German: *Nicht-Verbindlichkeit*)

*Code-alias: Non-/Limited-jurisdictional recommendations in CPGs (formal characteristic)*

## Focus topics

- Non-/Limited-jurisdictional recommendations in the form of non-binding pre-test-probabilities

## Example quote

**Non-/Limited-jurisdictional recommendations may only be loosely adhered to:** "It can be implemented that way, yes. But certainly, it does not have to. And I think in practice, least colleagues now just look strictly at these numbers." (B02, GP)

# Reliability (in German: *Verlässlichkeit*)

*Code-alias: Reliable recommendations in CPGs (content-related characteristic)*

## Focus topics

- Reliable recommendations in the form of checklists
- Reliable recommendations in the form of assessment aids

## Example quote

**Reliable recommendations may be easily adhered to:** "Okay, yes, because it sometimes also helps if you can check off such a CPG for yourself: ‘I diagnosed them according to the recommendations and/’ Yes, or: ‘With this laboratory constellation and so on, they simply have a low CCS probability’ or so, right?" (B11, CA)

# Abstract nature (in German: *Abstraktheit*)

*Code-alias: Generalisations in CPGs (formal characteristic)*

## Focus topics

- Generalisations regarding symptoms of complaint

## Example quote

**Generalisations regarding symptoms of complaints could inhibit adherence to CPGs regarding basic diagnostic decisions:** "Absolutely, absolutely. You can also take that with you for the CPG writers. CPGs are very popular and are also on the rise. But CPGs are also somehow, sometimes woodcut-like if they are applied too narrowly. That is my opinion. I am already an older colleague, and I am not completely opposed to these CPGs, but I am quite *critical*, because the CPGs/ Just basically regarding CPGs: They are used to try to establish rules for action, but the physician-patient reality is often different – not woodcut-like, but much more varied. Indeed, not woodcut-like, but it needs to be seen in a much more differentiated way. And this is not fulfilled by a CPG or care instructions, because the individual cases are very, very different: Individual cases are very, very individual. The symptoms are also very individual in terms of their severity." (B09, GP)

# Ambiguity (in German: *Ambiguität*)

*Code-alias: Gray areas in CPGs (content-related characteristics)*

## Focus topics

- Gray areas regarding pre-test probabilities

## Example quote

**Gray areas regarding pre-test probabilities could inhibit adherence to CPGs regarding basic diagnostic decisions:** "So, and in between there is a certain gray area. That is, under fifteen percent pre-test probability. And I honestly estimate that, I do not always look at the chart. It is a physician’s discretion. So, between five and fifteen percent it is a kind of medical judgment, ‘What are you going to do?’" (B01, CA)

# Incompleteness (in German: *Unvollständigkeit*)

*Code-alias: Gaps in CPGs (content-related characteristics)*

## Focus topics

- Gaps regarding gender specific aspects
- Gaps regarding socio-medical aspects

## Example quote

**Gaps regarding gender-specific aspects could inhibit adherence to CPGs regarding basic diagnostic decisions:** "There are major differences between male and female CCS patients. Male patients tend to have the classic chest pain, pain in the left arm. This is something that has now actually penetrated far into the population. And there are also studies about it, and this also corresponds to my own experience that women often have more unspecific symptoms. Sometimes they only have shortness of breath during exertion or some kind of malaise, nausea. Men can also have upper abdominal pain in case that the right coronary artery is affected. Overall, women do not have these very, very classic CCS symptoms which you now somehow know from every health program. And you have to listen a little bit more carefully. If they say, ‘I am no longer so efficient,’ then you really have to think about it. And there really are also studies on this. And they should certainly also be expanded, that it is somehow gender-specific for CCS, perhaps that is also considered in the CPGs. That CCS is really often overlooked in women. Or every now and then, let us say so, right?" (B06, GP)

# Ostensible clarity (in German: *Eindeutigkeit*)

*Code-alias: Definitions in CPGs (content-related characteristics)*

## Focus topics

- Definitions regarding CCS as a diagnosis
- Definitions regarding the diagnostic process of CCS

## Example quote

**Definitions regarding CCS diagnosis could inhibit adherence to CPGs regarding basic diagnostic decisions:** "Hmm <mumbling>. So, the stupid thing about the CPGs is: the CPGs assume a diagnosis. The moment someone tells me, ‘The patient has CCS,’ I can arrange the next steps half asleep. The problem is that people do not come in and say, ‘Doctor, I have CCS,’ or ‘I have acute coronary syndrome,’ or whatever, but they come in with very unspecific things. We have people who have *jaw pain* when they have ‘angina pectoris.’ We have people who have *throat pain* when they have ‘angina pectoris.’ We have people who just somehow have trouble breathing, for which there can be a thousand reasons. And they have/ So, when you say, ‘Do you have chest pain or chest pressure?’ ‘No. Not at all’ [imitates a fictitious dialogue between GP and patient]. […] Yes, if someone has chest pain, then I also immediately think of CCS. But it comes extremely unspecific in part." (B03, GP)

# Effort (procedural) (in German: *Verfahrensaufwand*)

*Code-alias: Procedure mobilisation*

## Focus topics

- Attentional efforts in the use of procedures
- Training efforts in the use of procedures
- Cost efforts in the use of procedures
- Time efforts in the use of procedures

## Example quote

**Intense training efforts could inhibit adherence to CPGs regarding the use of CMRI as NIT in patients with intermediately presumed CCS:** "In general, the problem with CMRI examinations is actually that/ That this is not possible at all – as far as stress CMRI or adenosine or dobutamine examinations are concerned – in the outpatient sector – at least in this area here with us – because very few have an outpatient authorisation for stress CMRI examinations. That is indeed/ There is a relatively high *hurdle* to get the corresponding approval. And this is basically already the challenge/ So, in the outpatient radiology area, it is rather not possible to get such an examination." (B15, CA)

# Workload (administrative) (in German: *Verwaltungsaufwand*)

*Code-alias: Bureaucracy*

## Focus topics

- Applications for SHI cost absorption/reimbursement as bureaucratic processes
- Procurement of medical findings as a bureaucratic process
- Processes within outpatient care as bureaucratic processes
- Inclusion of patients care contracts as a bureaucratic process
- Transmission of medication schedules and laboratory values as a bureaucratic process

## Example quote

**Lavish transmission of medication schedules could inhibit adherence to CPGs regarding medication:** "For example, also about decisions regarding medication. That I as a CA, for example, often do not have any laboratory values available for the patients, right? […] So, there I see the/ So, also again an improvement suggestion in it or an/ Yes, an improvement, if one is well networked and, accordingly, I would say, get the data." (B15, CA)

# Economic structure (in German: *Ökonomische Struktur*)

*Code-alias: Financial accesses*

## Focus topics

- Cost absorption of healthcare services within the healthcare system
- Reimbursement for healthcare providers within the healthcare system

## Example quote

**Insufficient SHI reimbursement for healthcare providers could inhibit adherence to CPGs regarding the use of CCTAs and CMRIs as NITs in patients with intermediately presumed CCS:** "But this is recommended in the *golden* CPG and no health insurance company pays for it, right? So, you can just forget about it. That really annoyed me because that is a kind of privileged medicine. And *this* is then the CPG, right? CMRI or CCTA? And that is then only available to a small number of private patients, because there is no prior cost coverage check. And for ninety percent to ninety-two percent of patients not, right? And then I think to myself: what kind of *weak* CPG is that? Of course, those are great procedures, but it is not available to me at all, right?" (B04, GP)

# Local structure (in German: *Lokale Struktur*)

*Code-alias: Spatial accesses*

## Focus topics

- Reachability of healthcare services within the healthcare system
- Reachability of healthcare providers within the healthcare system

## Example quote

**Insufficient reachability of healthcare services could inhibit adherence to CPGs regarding the use of CCTA as NIT in patients with intermediately presumed CCS:** "Yes, CCTA is actually not offered here in my region. Yes, so you have to be very clear about that. I have had maybe two patients in the last fifteen years where that was done for various reasons – which would now be too complicated to explain, I think. But that/ In the CCS consideration, it plays no role in fact." (B05, GP)

# Stipulated structure (in German: *Vertragliche Struktur*)

*Code-alias: Contractual accesses*

## Focus topics

- Collective contract offers within the healthcare system
- Selective contract offers within the healthcare system

## Example quote

**Insufficient selective contract offers could inhibit adherence to CPGs regarding the use of CCTAs and MPSs as NITs in patients with intermediately presumed CCS:** "That is how it is. That is the very big/ And I do not know of any integrated care contract here now, either, in which/ Just a moment, got to go to the other line [B12 is on another line for about half a minute, music on hold]. So, here I am again. No, the problem is that I do not know of any integrated care contracts that really reflect this in a reasonable way. That nuclear physicians or radiologists are involved, that innovative procedures such as CCTA as well as/ Well, now MPS is not quite as innovative (laughs), but at least can then also be used somehow as it is written in the CPG, right?" (B12, GP)

# Temporal structure (in German: *Zeitliche Struktur*)

*Code-alias: Time-related accesses*

## Focus topics

- Waiting times for healthcare services within the healthcare system
- Waiting times for healthcare providers within the healthcare system

## Example quote

**Insufficient waiting times to healthcare providers could inhibit adherence to CPGs regarding outpatient referral to CAs as secondary care providers:** "If you do not refer them to the hospital now, but say, ‘I think you need to see a CA,’ the […] logistical difficulty is that we do not get any appointments. The normal CA appointments/ If we get one four months later, we are already lucky. So, I do not know if that is what you mean and want to hear, but those are just the things where you say, ‘It could be that easy, but it is not.’" (B03, GP)
